# A Systematic Review of Outcome Measures Use, Analytical Approaches, Reporting Methods, and Publication Volume by Year in Low Back Pain Trials Published between 1980 and 2012: *Respice, adspice, et prospice*

**DOI:** 10.1371/journal.pone.0164573

**Published:** 2016-10-24

**Authors:** Robert Froud, Shilpa Patel, Dévan Rajendran, Philip Bright, Tom Bjørkli, Rachelle Buchbinder, Sandra Eldridge, Martin Underwood

**Affiliations:** 1 Department of Health Sciences, Kristiania University College, Oslo, Norway; 2 Warwick Medical School, University of Warwick, Coventry, United Kingdom; 3 European School of Osteopathy, Maidstone, Kent, United Kingdom; 4 Monash Department of Clinical Epidemiology, Cabrini Institute and Department of Epidemiology and Preventive Medicine, School of Public Health and Preventative Medicine, Monash University, Malvern, Victoria, Australia; 5 Barts and the London School of Medicine and Dentistry, Queen Mary University of London, London, United Kingdom; Centre Hospitalier Universitaire Vaudois, SWITZERLAND

## Abstract

**Background:**

Increasing patient-reported outcome measures in the 1980s and 1990s led to the development of recommendations at the turn of the millennium for standardising outcome measures in non-specific low back pain (LBP) trials. Whether these recommendations impacted use is unclear. Previous work has examined citation counts, but actual use and change over time, has not been explored. Since 2011, there has been some consensus on the optimal methods for reporting back pain trial outcomes. We explored reporting practice, outcome measure use, and publications over time.

**Methods:**

We performed a systematic review of LBP trials, searching the European Guidelines for the management of LBP, extending the search to 2012. We abstracted data on publications by year, outcome measure use, analytical approach, and approaches taken to reporting trials outcomes. Data were analysed using descriptive statistics and regression analyses.

**Results:**

We included 401 trials. The number of published trials per year has increased by a factor of 4.5 from 5.4 (1980–1999) to 24.4 (2000–2012). The most commonly used outcome measures have been the Visual Analogue Scale for pain intensity, which has slowly increased in use since 1980/81 from 20% to 60% of trials by 2012, and the Roland-Morris Disability Questionnaire, which rose to 55% in 2002/2003, and then fell back to 28% by 2012. Most trialists (85%) report between-group mean differences. Few (8%) report individual improvements, and some (4%) report only within-group analyses. Student’s *t* test, ANOVA, and ANCOVA regression, or mixed models, were the most common approaches to analysis.

**Conclusions:**

Recommendations for standardising outcomes may have had a limited or inconsistent effect on practice. Since the research community is again considering outcome measures and modifying recommendations, groups offering recommendations should be cognisant that better ways of generating trialist buy-in may be required in order for their recommendations to have impact.

## Introduction

Patient-reported outcome measures (PROMs) are outcomes that are reported by patients, rather than being objectively assessed or involving third-party (*e.g.* clinician) judgement. Throughout the 1980s and 1990s, multiple back-specific PROMs were developed and began to dominate as outcome measures used in non-specific low back pain (nsLBP) trials. Between 1998 and 2000 recommendations were made to standardise outcome measure use to facilitate cross-trial comparisons, pooling of data, and encourage scale familiarity. [1–3] More recently, researchers and clinicians have again begun to question whether the right things are being measured and there have been calls to review the outcome measures used in trials. [4–8] It is not clear whether the millennial recommendations for standardisation had an effect on practice. Previous studies have explored the number of times back-specific measures have been cited, but not actual use; also, trends of use over time have not hitherto been explored. [9, 10]

Results from trials using PROMs can be reported differently, and this is known to affect clinicians’ interpretations of effectiveness and subsequent decision-making. [11, 12] With this in mind, recommendations for reporting outcomes in back pain trials were made in 2011 and 2014. [13, 14]

We aimed to explore actual use of outcome measures in nsLBP trials, between 1980 and 2012, spanning the publication point of the millennial core-set recommendations. Our objectives were to identify the most commonly used outcomes, and the domain coverage of back-specific PROMs, and to consider whether there was any change in the trajectories of outcome measure use over the period of interest. Additionally, we reviewed the number of publications over time, reporting methods and analytical approaches for the most commonly used outcome measures to provide a baseline assessment of current practice so that any future change may be monitored.

## Materials and Methods

Two independent reviewers (RF and SP) identified randomised controlled trials (RCTs) of any intervention for nsLBP published in or after 1980, from COST-B13’s European Guidelines for the Management of Low Back Pain (EGLBP), which included a comprehensive systematic search of all interventions for nsLBP, and the systematic reviews reported in the EGLBP. [15] As the COST-B13 search ended in November 2002, we extended the search to January 1, 2007 using the Cochrane Library, EMBASE, Lilacs, PsycINFO, and PubMed databases, and we hand-searched the Health Technology Assessment (HTA) journal. We later updated the search to January 1, 2012, using the Cochrane Library, PubMed, and EMBASE database. We omitted PsycINFO, Lilacs, and the HTA journal in this extension due to good cross-coverage from the other databases (see Discussion). An example search strategy is included as a supplementary file (S1 Text). We combined material from the EGLBP and the extended searches, removed duplicates, and short-listed by title and abstract. Full-texts were obtained if the titles and abstract alone contained insufficient information for assessment against the criteria listed in Table 1.

**Table 1:** Inclusion and exclusion criteria.

Order	Inclusion criterion
1	RCTs of nsLBP
	**Exclusion criteria**
1	Non-English language reports
2	Studies that were not RCTs or presented insufficient information for us to determine whether randomisation was used to allocate participants
3	Reports that self-identified as pilot/feasibility studies
4	Cross-over designs (because of limited utility in the LBP field)
5	RCTs with mixed samples (*e.g.* neck or thoracic pain in addition to LBP), samples of participants with radiating leg pain, or referred pain extending past the knee, or samples including LBP specific pathology (*e.g* cancer, ankylosing spondylitis, or disc herniation) or pregnancy
6	Non-inferiority trials (because of limited utility in the LBP field)
7	Follow-up studies with no new outcome measures, and multiple publications. In the case of multiple publications, we included the first published article and excluded subsequent publications

RCT = Randomised controlled trial; nsLBP = non-specific low back pain; LBP = low back pain.

### Data abstraction and validation

Two reviewers (either RF, SP, TB, PB, or DR) independently abstracted data on outcome measure use, details of primary outcome analysis, and reporting methods. An outcome was identified as ‘primary’ if (1) the outcome was nominated as such; if no outcome was nominated, or multiple outcomes were nominated, we used (2) the outcome measure on which the sample size calculation was based; if this was not reported, we identified (3) the first outcome measure referred to in the abstract; and if this was not identified, we used (4) the first outcome mentioned in the paper. We identified the primary time point of interest, or used the first follow-up time point in cases when this was not clear. This approach has been taken in other methodological reviews. [16–20] For comparison, a sensitivity analysis of primary outcome measure use for the most commonly used outcome measures was performed using only criterion 1 and 2. Disagreements were resolved through discussion and, if necessary, with arbitration and a third reviewer (RF, SE, or MU).

Using Microsoft Visual Basic 6.3 (Microsoft, Washington) and Microsoft Office Excel 2003 (Microsoft, Washington), we developed a front-end program to assist the data abstraction process and manage abstracted data, which validated entries and provided alerts in the case of missed fields.

For outcome measure identification, we used expert validation of 20% of papers, as has been done in other methodological reviews. [21] Half (*i.e.* 10%) were selected at random and half (*i.e.* 10%) were purposively sampled (papers that we anticipated might lead to disagreement) from the papers marked for full-text extraction and then given to an independent reviewer (either SE, RF, SP, TB, PB, or DR), for independent abstraction. Since early in the process we observed good (> 80%) agreement on outcome measurement identification, but inadequate agreement on analytical and reporting methods, full independent abstraction was subsequently used to identify analytical approach and reporting methods used. Disagreements were settled by arbitration involving one of the statisticians (either RF or SE). Quality of included trials was not evaluated, since we were interested in all non-specific back pain trials, regardless of the trials’ methodological quality.

### Analysis

To report the proportion of outcome measure use by year, the total number of trials is needed as the denominator. For this reason, we first explored the number of published nsLBP trials by year. Prevalence of outcome measure use and in the case of PROMs, domain of measurement, was then calculated for primary and secondary outcomes, by year. We were particularly interested in PROM use, and we did not differentiate between different types of objective outcome measure use, or clinical judgements.

Methods for reporting the two most commonly used outcome measures and the types of analysis used were summarised using descriptive statistics and graphical methods. For reporting methods, we explored the statistics used to summarise central tendency and variance, graphical forms of representation, and use of tables. For analytical approach, we explored the statistical test used to test the null hypothesis that a between group difference was zero, or the model that was fitted to the data. We used regression analysis to explore the relationship between publications and time, and outcome measure use and time, fitting polynomial terms if relationships were non-linear. Residuals from regression analysis were examined for fit. If data were too heteroschedastic (*i.e* variance of an outcome variable was dependent on the value of a predictor variable) for regression modelling, we fitted locally weighted scatter plot smoothing (Lowess) lines, which has the effect of smoothing across erratic data points, so that trends can be more easily visualised. All analyses were performed in Stata, version 12 (Statacorp, Texas). We did not publish a review protocol ahead of undertaking this work.

## Results

**Fig 1:**
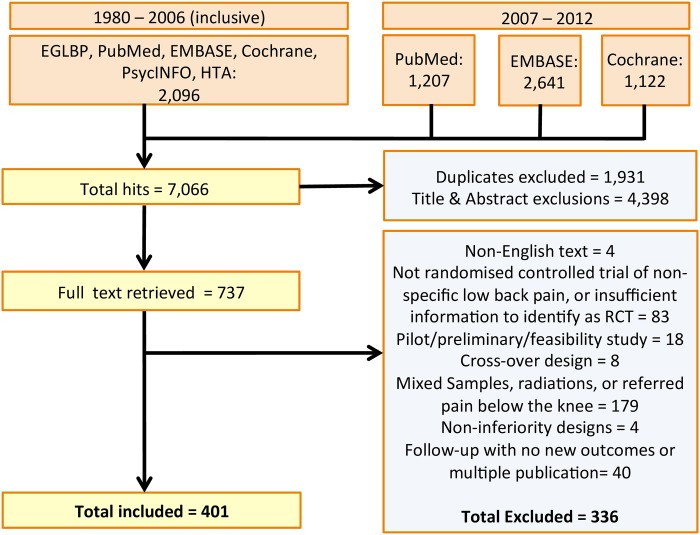
Flow chart showing search results. The figure shows the number of initial hits, duplicates, exclusions based on titles and abstracts screening, and assessments at full text level evaluation.

We identified 7,066 potential articles from EGLBP and electronic databases and following removal of duplicates, titles and abstracts sifting, and full-text inspection against inclusion criteria, included 401 trials (Fig 1). Characteristics of included and excluded trials are detailed with their references as supplementary material (S1 and S2 Tables).

### Publications over time

**Fig 2:**
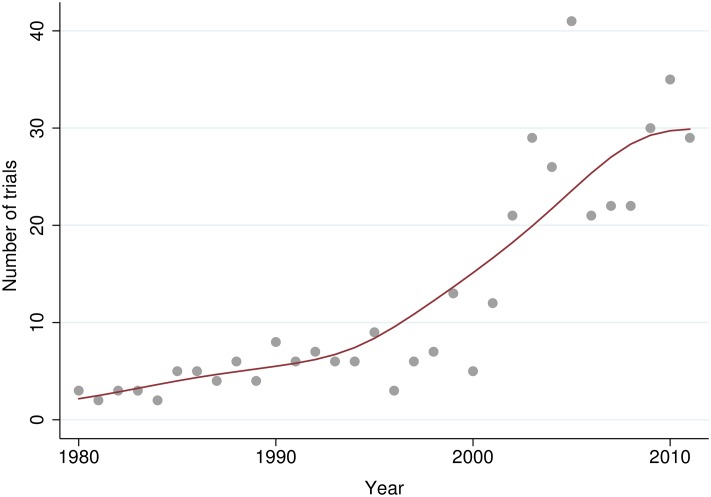
Number of published non-specific low back pain trials by publication year between 1980 and 2012. The figure shows the increase in the number of published non-specific low back pain trials by year of publication and change in publication rate over time. A Lowess smoother is fitted to these data.

A linear regression model with a quadratic term in year was significant (*β*_*year*_ = −166.187, *P* = 0.002, *β*_*year*^2^_ = 0.041, *P* = 0.002), and explained 79% of the variance (Fig 2). Some caution must be noted with respect to interpretation, since as can be seen from Fig 2, these data are slightly heteroschedastic. The number of publications increases after the millennium; the average number of publications per year from 1980 to 1999 is 5.4 and from 2000 to 2012 is 24.4.

### Outcome measure use

Authors explicitly declared a primary outcome measure in 50% (*n* = 201) of trials. In trials that did not declare one, a primary outcome measure could be identified from a sample size calculation in 20% (*n* = 40) of trials. We identified the primary as the first outcome measure mentioned in the abstract, or in the paper, in the remaining 40% (*n* = 161).

**Fig 3:**
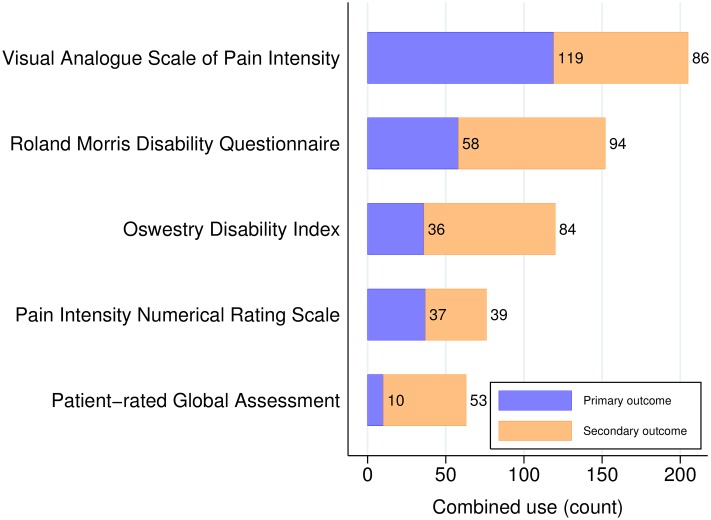
The five most common back-specific patient reported outcome measures. The figure shows the use of the five most common back-specific patient reported outcome measures as primary and secondary outcome measures.

The most commonly used PROMs were the Visual Analogue Scale for measuring Pain intensity (VAS-P), [22], and the Roland Morris Disability Questionnaire (RMDQ), [23] Oswestry Disability Index (ODI), [24] Numerical Rating Scale for measuring pain intensity (NRS), [25, 26] and patient-rated global assessment of improvement (*i.e.* a health transition question (TQ) [27]) (Table 2 and Fig 3).

**Table 2:** The most common back-specific PROMs: Frequency of use.

Instrument	Primary outcome	Secondary outcome	Total use	Primary use (%)	Sensitivity analysis (%)^†^
Visual Analogue Scale of back pain intensity [22]	119	86	205	29.7	28.2
Roland-Morris Disability Questionnaire [23]	58	94	152	14.5	20.7
Oswestry Disability Index [24]	36	84	120	9.0	10.4
Pain Intensity Numerical Rating Scale [25, 26]	37	39	76	9.2	8.7
Patient Rated Global Assessment (TQ) [27]*	10	53	63	2.5	2.5

PROM = Patient-reported outcome measure

* Example reference only

^†^ Of proportional use as primary outcome measure

We note that we class the VAS-P and NRS as back-specific, since the wording of these instruments, when used in back pain trials, typically refers specifically to a person’s back pain. In total, there were 258 different PROMs used across the trials studied within the time period.

Sensitivity analyses of primary outcome measure use within only trials that either explicitly declared the outcome measure as primary, or used it for a sample size calculation, revealed similar estimates of primary outcome measure use in across outcome measures, with the possible exception of the RMDQ (Table 2).

Apart from PROMs, objectively assessed outcome measures were also common (*n* = 130), particularly as secondary outcome measures, as were medication and medical services consumption (*n* = 66), and subjective clinical examinations (*n* = 19).

**Fig 4:**
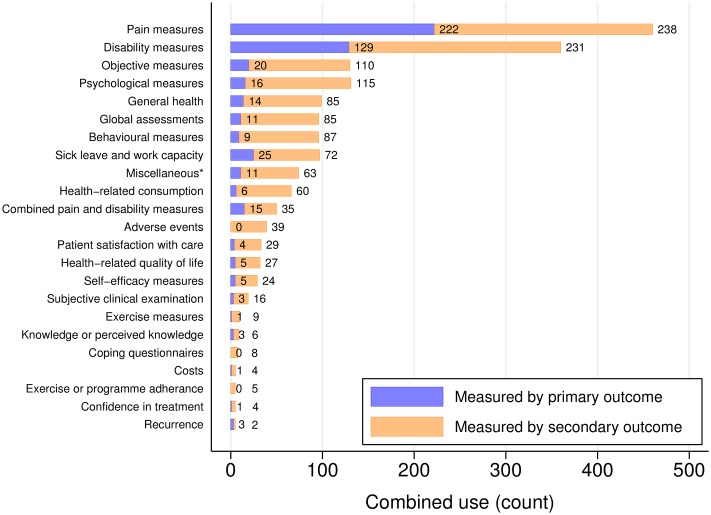
Domains of measurement in non-specific low back pain trials. The figure shows the domains of measurement in non-specific low back pain trials published between 1980 and 2012.

Pain and disability were the most commonly measured domains (Fig 4). Some outcomes, for example, adverse events, or adherence, have only ever been measured as secondary outcomes. We note that it is possible for the usage to exceed the number of included trials, which is due to some trials using more than one outcome measure to measure within these domains.

**Fig 5:**
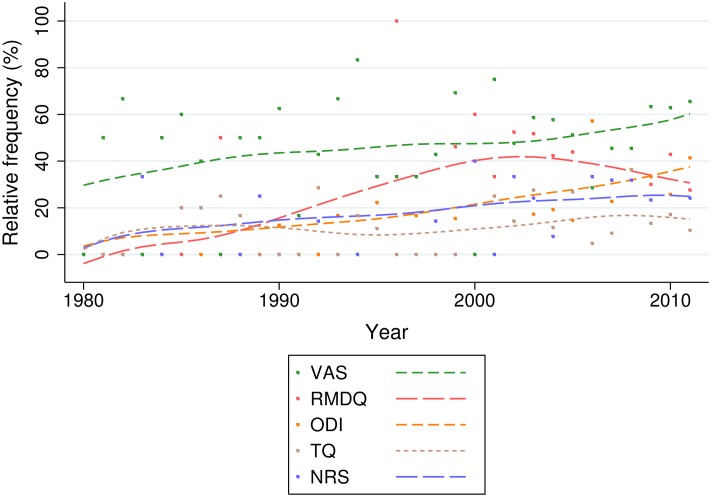
The five most common back-specific patient-reported outcome measures: Relative use by year. The figure shows relative frequency of use for the most common back-specific patient-reported outcome measures, by publication year.

The VAS-P has had a slowly increasing usage as an outcome measure in nsLBP trials, increasing from from 20% in slowly increased in use since 1980/81 to 60% of trials by 2012. There is some suggestion of a rise and fall in the use of the RMDQ, which peaks at 55% in 2002/2003. Use of the ODI has steadily increased. These data were too heteroschedastic for regression analysis and we present the data graphically with a Lowess smoother (Fig 5). We note the use of a large smoothing bandwidth to easily visualise trends; while the RMDQ smoother intersects the x-axis at 1982, the year of its publication, it was not recorded as used in any included trials until 1987. As such, these Lowess lines should be interpreted as a general impression of trend only. We have included as an additional file (S1 Fig) the plot with a smoother of half the bandwidth, to depict more sensitive trend-lines.

### Reporting methods and analytical approach

For the two most commonly used outcomes, the VAS-P and the RMDQ, reporting methods include describing mean changes (within groups) or mean differences (between groups), P-values for changes or differences, and standard deviations (Table 3). Confidence intervals were only provided in around 40% of trials. Few authors reported individual improvements. The *t* test has been the most common analytical approach, followed by regression analyses. The most common covariates used in adjusted models were baseline score, sex, age, study centre, and episode duration, respectively. In 4% of trials utilising the VAS-P or RMDQ, authors present within-group analyses only.

**Table 3:** Reporting methods and statistical analysis: Prevalence of use.

Details	VAS-P(%)	RMDQ(%)	Totals(%)
Usage	205 (100)	152 (100)	357 (100)
**Reporting method**			
Mean or mean difference	179 (87)	126 (83)	300 (85)
P-values	162 (79)	109 (72)	271 (76)
Standard deviation	134 (65)	98 (64)	232 (65)
Median	22 (11)	14 (9)	36 (10)
Range or IQR	20 (10)	13 (9)	33 (9)
Standard error	18 (9)	18 (12)	36 (10)
Confidence intervals	62 (30)	76 (50)	138 (39)
Number/proportion improved*	20 (10)	8 (5)	28 (8)
Number needed to treat	2 (1)	5 (3)	7 (2)
Odds ratio (improvement)	3 (1)	9 (6)	12 (3)
Relative risk (improvement)	1 (0)	6 (4)	7 (2)
Percentage change score	25 (12)	15 (10)	40 (11)
Standardised mean difference	9 (4)	11 (7)	20 (6)
**Graphs and tables**			
Table	174 (85)	119 (78)	293 (82)
Line chart	60 (29)	39 (26)	99 (28)
Bar chart	29 (14)	9 (6)	38 (11)
Other	8 (4)	9 (6)	17 (5)
**Statistical analysis**			
ANCOVA regression / mixed model	53 (25)	64 (42)	117 (33)
ANOVA/MANOVA regression / mixed model	74 (36)	39 (26)	113 (32)
*t* test	93 (45)	43 (28)	136 (38)
Non-parametric test^†^	68 (33)	37 (24)	105 (29)
Other	18 (9)	9 (6)	27 (8)
Within-group analysis only	11 (5)	3 (2)	14 (4)
Test not described	10 (5)	10 (7)	20 (6)

VAS = Visual analogue scale

RMDQ = Roland-Morris Disability Questionnaire

IQR = Interquartile range

* Where ‘improvement’ is defined by the change in score of a specified magnitude

^†^ *e.g.* Wilcoxon, Friedman’s, Mann Whitney *U*, or Kruskal Wallis

## Discussion

We discuss the results and consider their implications in three sections. We consider these data and past recommendations for core sets of outcome measures in back pain trials (*Respice*), the current situation and emerging recommendations for core sets (*Adspice*), and implications for the future of outcome measure use in back pain trials (*Prospice*).

### *Respice* 

The results show that the most commonly used domains in back-specific outcome measures over the whole time-period of interest are domains and PROMs that were included in the millennial recommendations. The millennial recommendations of Deyo *et al* in 1998, updated by Bombardier *et al* in 2000, recommend measurement in the domains of pain, function, well-being, disability, and work disability and both recommended using the VAS-P and NRS for measuring pain, and the RMDQ or ODI for measuring function (disability). [1, 2] The World Health Organisation made back-specific recommendations to measure pain, disability, and depression, in 1999, recommending the VAS-P and the ODI be used as primary outcome measures in trials. [3] International Classifications of functioning (ICF) categories were later proposed, recommending 78 (comprehensive) or 35 (brief) domains of measurement for LBP in 2004. [28] The brief set, intended in particular for use in clinical studies, included body functions, structures, activities and participation and environmental factors. The recommendations were criticised for having incomplete coverage. [29]

If the millennial recommendations for measurement instruments had impacted practice then notwithstanding the time it takes for recommendations to be implemented, one might expect relative increases in proportional use trajectories sometime after the publication of the recommendations at the turn of the millennium. There is possibly a post-millennial increase in trajectory in the VAS-P and ODI; there may be a decrease in use of the RMDQ. As these data represent a population of trials over the time period, inferential statistics are unnecessary, and the millennial recommendations appear to have had a limited or inconsistent impact on actual use.

### *Adspice* 

Towards the end of the 1980–2012 period of interest, researchers suggested outcome measures are inadequate and that their reassessment needed to be prioritised. [4, 7] A second wave of recommendations is now emerging. In 2011, Buchbinder *et al* identified several domains of measurement not covered by existing core sets, including loss of independence, worry about the future, and negative or discriminatory actions by others. [30] In 2012, Hush *et al* recommended adding the Patient Generated Index (PGI) and a global back pain recovery scale to the core set, specifically to measure recovery. [31] In 2014, Froud *et al* showed social components were not adequately represented in measurement. [32] The results in the current study highlight the absence of measurement in social domains; although social factors are covered to some extent within the domain of health-related quality of life.

In 2014, a National Institutes of Health (NIH) Task Force recommended using Patient Reported Outcomes Measurement Information System (PROMIS) measures as a minimum dataset in all NIH-funded LBP research; permitting the RMDQ to substitute the PROMIS physical activity items if more extensive ‘legacy measures’ are required. [14] In 2015, Froud *et al* extended the Hush recommendation for patient-centric instruments, such as the PGI and TQ, for trial outcome measurement more generally, after demonstrating that people may not attend to thinking about their back pain when completing the RMDQ and that changes in RMDQ score can be independent of changes in back pain. [8, 31] In 2015, Chiarotto *et al* recommended three domains for inclusion in a core set, including physical functioning, pain intensity and health-related quality of life. [33]

Thus, it may currently be difficult from a trialist’s perspective to decide upon appropriate primary and secondary outcomes in the design-phase of nsLBP trials. We suggest that this requires careful future attention from groups making core set recommendations, and we discuss this in the next section. In the meantime, given the widespread use shown in the results, there may be some value in using either the VAS-P or RMDQ as an outcome measure in nsLBP trials as this facilitates the greatest cross-comparison. Choices of secondary outcomes might include some of the more patient-relevant and coverage-improving domains, such as the PGI and TQ. The results showed that some domains (for example, adverse events and adherence) are only ever measured as secondary outcomes. In our view this is reasonable (unless the research question relates to adverse events) since the primary focus across most trials and core set recommendations has emphasis on pain and function.

### *Prospice* 

There is a risk that overlaps or contractions between and within the millennial and current recommendations may continue to give a discordant message to trialists. For example, the millennial Deyo/Bombardier recommendations suggested use of VAS-P or NRS (pain) and RMDQ or ODI (function) whereas the World Health Organisation recommended only VAS-P and ODI for measuring these domains. Currently, researchers are taking slightly different directions with their recommendations for the future (see above). Future divergences in recommendations may benefit from clear justification and argument. One driving issue may be that researchers disagree upon fundamental clinical measurement properties of instruments. For example, although the RMDQ is the second most commonly used outcome in nsLBP trials, it has gained both criticism and support of its clinimetric properties. [8, 27, 34] An underlying issue here may be disagreement on and heterogeneity in how key clinimetric assessments, such as test-retest studies and responsiveness studies, are conducted. [35–37] The Consensus-based Standards for the Selection of Health Measurement Instruments (COSMIN) checklist may go some way towards helping with this, and it may be that new instruments, with a focus on performance, coverage, and patient-relevance need to be developed prior to future recommendations being made. [38]

The historical data in the current study suggest that the current wave of recommendations may be set to have limited or inconsistent impact on practice. In future, consideration may need to be given to mechanisms for maximising impact of recommendations and affecting change. We suggest that to maximise impact it may be worth considering (1) the *consistency* of recommendations, which might be facilitated through (2) *inclusivity*—a more widespread and comprehensive collaboration between methodologists working on outcome measures, along with clinimetric/psychometric experts, and domain-specific experts where required, in addition to clinical experts. One approach to achieving this may be Delphi technique, which is supported by the Core Outcome Measures in Effectiveness Trials (COMET) initiative, with consideration given to fully reporting panel disagreement and lack of consensus. [39] Finally, generating sufficient (3) *participation* or *‘buy-in’* from trialists with acquisition of support, and a clear and implementable transition strategy to manages the large lag-times from trial conception to publication, may help to avoid fragmentation in outcome measure use. It may be that amendments to well-adopted check-lists would also help. The Standard Protocol Items: Recommendations for Interventional Trials (SPIRIT) check list strongly encourages trialists to explain the clinical relevance of chosen outcomes in trial protocols (item 12). [40] However, for trial reporting, the Consolidated Standards of Reporting Trials (CONSORT) statement (in-particular item 6a), might be modified to encourage authors to discuss any divergences from recommended core sets or justify outcome measurement selection, which over time, may help to homogenise outcome measurement across similar trials. [41] Additionally, it may be that public and private funders, as stakeholders with a vital interest in maximising the use of clinical trial data, are well-placed to encourage trial teams to carefully consider outcome selection prior to their awarding a grant for a trial.

### Reporting methods and analysis

Specific recommendations for including individual improvements when reporting the outcomes of back pain trials were not made until 2011. [13] These were reiterated by the NIH task force in 2014. [14] The 1980 to 2012 data in the current study are not consistent with the recommendations, but the results of the current study will allow future monitoring of the impact of these or future recommendations.

It is encouraging that the majority of authors report between-group differences and a reasonable proportion (33%) report adjusted models. Adjusted models improve the precision of estimates by taking account of imbalances that exist between groups notwithstanding randomisation. [42] Few authors have used inappropriate analytical methods, such as analysing only within-group changes; meaning it is not possible to differentiate treatment effect from regression to the mean.

### Comparisons to existing research

Litcher-Kelly *et al* report, from a systematic review of musculoskeletal clinical trials, that the most frequently used instruments were the VAS (60%), and the NRS (12%), with an RMDQ prevalence of 14%. [43] These data are not directly comparable to ours since the study population and publication years differ. In 2004, Müller *et al* identified 84 different back-specific PROMs and showed that the Oswestry Disability Questionnaire and the RMDQ were most commonly cited. [9] In 2011, Chapman *et al* also reviewed which outcome measures had been cited in back pain trials between 2006 and 2011. [10] They found that the most cited functional measure was the ODI and that the most cited pain measure was the NRS. Counting citations may not reflect actual use. In some cases, citations may not be given and for long-running outcome measures, such as the VAS, references may not be uniform. For example, Huskisson is commonly credited with developing the VAS, in 1974, but he is not uniformly credited with it. [22] There is evidence that the VAS was being used at least as far back as 1921. [44] Also, some citations will reference validatory work.

In other fields, Araújo *et al* have shown that recommendation of core sets of measurement in gout have not impacted practice. [45] Page *et al* showed in 2015 that shoulder pain trials suffer from having no core measurement sets recommended. [46] Relatively the situation in back pain research may be similar or better, respectively.

Nuovo *et al* examined the prevalence of reporting of absolute risk reduction (ARR) and number needed to treat (NNT) in RCTs published in five mainstream medical journals. [47] Evaluating publications in 1989, 1992, 1995, and 1998 they found that amongst 359 articles that ARR was reported in 18 (5.0%) reports, and that NNT was used in eight (2.2%). This is similar to our study, in which 8% reported the number or proportion of improvements (a coding in our review which subsumed ARR), and 2% of trials reported NNT.

### Strengths and limitations

A strength of our research is provision of prevalence data based on actual use, rather than using citations as a proxy. Other authors have reviewed what outcome measures exist for LBP, but have not estimated prevalence of use. [48–50]

We acknowledge that it is preferable to conduct a systematic review using a singular search pattern. As the EGLBP search was comprehensive, included all interventions, and we had previously used it to identify all nsLBP trials, we reasoned it had good cross-coverage with out later search. Also, we did not search PsycINFO and HTA reports, due to good cross-coverage from the other databases. This fragmented search strategy may be viewed as a limitation. However, in comparison to Castellini *et al*, who reviewed back pain trials published after 1968 and included 222 trials, we judge our search to have been comprehensive. [51]

We adopted a 20% validation approach rather than full independent reviewer extraction for all variables. We used full independent abstraction when abstracting details of reporting methods and analysis as validation revealed inadequate agreement only on these variables. While full independent abstraction on all variables would have improved validity, as our focus was methodological and not on estimating a treatment effect, we considered the approach to be reasonable.

Our sensitivity analysis, using only the first two of four criteria for judging a primary outcome measure, showed similar results for estimates of prevalence of use for all but the RMDQ. Few additional (only eight instances) RMDQ primary outcomes were identified using criteria 3 or 4. It may be that, unlike users of other primary outcome measures, those who use the RMDQ as a primary are relatively more likely to explicitly declare it as a primary, or perform a sample size calculation based on detecting a difference in RMDQ score between groups. We note that the difference between estimating prevalence of use using only the first two criteria, or all four criteria, does not change the relative ranking of most common usage of primary outcome measures and that, using either method, the RMDQ is the second most commonly used primary outcome measure.

### Conclusions

The Visual Analogue Scale of pain intensity and the Roland Morris Disability Questionnaire have been most commonly used in back pain trials. Recommendations for standardising outcomes may have had a limited or inconsistent effect on practice. Analytical and reporting practice is encouraging, although there is still room for improvement. Research groups planning to make further recommendations on core outcome measures for back pain may have more impact if they consider better ways of generating trialist buy-in.

## Supporting Information

S1 Text Typical search strategy. A typical search strategy used in the systematic review.
(PDF)

S1 Table Characteristics of included trials. A table showing the characteristics of included trials and their references.
(PDF)

S2 Table Characteristics of excluded trials. A table showing the characteristics of excluded trials and their references.
(PDF)

S1 Fig Most commonly used measures over time, standardised by the number of annual publications. The figure shows the most commonly used measures over time, standardised by the number of annual publications, using a Lowess smoother of half the bandwidth of that shown in Fig 5, in case more sensitive trend-lines are preferred.
(TIF)

S1 Checklist PRISMA checklist. A completed PRISMA checklist for the systematic review.
(PDF)

## References

[1] Deyo RA, Battie M, Beurskens AJ, Bombardier C, Croft P, Koes B, et al Outcome measures for low back pain research. A proposal for standardized use. Spine. 1998; 23(18):2003–13. 10.1097/00007632-199809150-00018 9779535

[2] Bombardier C. Outcome assessments in the evaluation of treatment of spinal disorders: Summary and general recommendations. Spine. 2000; 25(24):3100–3. 10.1097/00007632-200012150-00003 11124724

[3] Jayson M: Outcome measures for back pain: introduction, justification, and epidemiology in “Low back pain initiative” Geneva: World Health Organisation; 1999.

[4] Foster NE, Dziedzic KS, van der Windt DA, Fritz JM, Hay EM. Research priorities for non-pharmacological therapies for common musculoskeletal problems: nationally and internationally agreed recommendations. BMC Musculoskelet Disord. 2009; 10:3 10.1186/1471-2474-10-3 19134184 PMC2631495

[5] Mullis R, Barber J, Lewis M, Hay E. ICF core sets for low back pain: do they include what matters to patients? J Rehabil Med. 2007; 39(5):353–357. 10.2340/16501977-0059 17549324

[6] Hush JM, Refshauge K, Sullivan G, Souza LD, Maher CG, McAuley JH. Recovery: what does this mean to patients with low back pain? Arthritis Rheum. 2009; 61:124–131. 10.1002/art.24162 19116958

[7] Hush JM, Refshauge KM, Sullivan G, De Souza L, McAuley JH. Do numerical rating scales and the Roland-Morris Disability Questionnaire capture changes that are meaningful to patients with persistent back pain? Clin Rehabil. 2010; 24(7):648–57. 10.1177/0269215510367975 20530647

[8] Froud R, Ellard D, Patel S, Eldridge S, Underwood M. Primary outcome measure use in back pain trials may need radical reassessment. BMC Musculoskelet Disord. 2015; 16:88 10.1186/s12891-015-0534-1 25887581 PMC4419506

[9] Muller U, Duetz MS, Roeder C, Greenough CG. Condition-specific outcome measures for low back pain. Part I: Validation. Eur Spine J. 2004; 13(4):301–13. 10.1007/s00586-003-0665-1 15029488 PMC3468051

[10] Chapman JR, Norvell DC, Hermsmeyer JT, Bransford RJ, Devine J, McGirt MJ, et al Evaluating common outcomes for measuring treatment success for chronic low back pain. Spine. 2011; 36(21 Suppl):S54–S68. 10.1097/BRS.0b013e31822ef74d 21952190

[11] Froud R, Underwood M, Carnes D, Eldridge S. Clinicians’ perceptions of reporting methods for back pain trials: a qualitative study. Br J Gen Pract. 2012; 62(596):e151–e159. 10.3399/bjgp12X630034 22429424 PMC3289820

[12] McGettigan P, Dianne S, O’Connell K, Hill S, Henry D. The Effects of Information Framing on the Practices of Physicians J Gen Intern Med. 1999; 14(10): 633–642. 10.1046/j.1525-1497.1999.09038.x 10571710 PMC1496755

[13] Froud R, Eldridge S, Kovacs F, Breen A, Bolton J, Dunn K, et al Reporting outcomes of back pain trials: A modified Delphi study. Eur J Pain. 2011; 15(10):1068–74. 10.1016/j.ejpain.2011.04.015 21596600

[14] Deyo RA, Dworkin SF, Amtmann D, Andersson G, Borenstein D, Carragee E, et al Report of the NIH Task Force on research standards for chronic low back pain. Spine J. 2014; 14(8):1375–1391. 10.1016/j.spinee.2014.05.002 24950669

[15] COST B13. European guidelines for the management of low back pain. Eur Spine J. 2006; 15(S2).

[16] Eldridge S. Assessing, understanding, and improving the efficiency of cluster randomised trials in primary care. PhD thesis. Queen Mary University of London; 2005.

[17] Eldridge S, Ashby D, Bennett C, Wakelin M, Feder G. Internal and external validity of cluster randomised trials: systematic review of recent trials. BMJ. 2008; 336(7649):876–80. 10.1136/bmj.39517.495764.25 18364360 PMC2323095

[18] Diaz-Ordaz K, Froud R, Sheehan B, Eldridge S. A systematic review of cluster randomised trials in residential facilities for older people suggests how to improve quality. BMC Med Res Methodol. 2013; 127:13 10.1186/1471-2288-13-127 24148859 PMC4015673

[19] Froud R, Bjørkli T, Bright P, Rajendran D, Buchbinder R, Underwood M, et al The effect of journal impact factor, reporting conflicts, and reporting funding sources, on standardized effect sizes in back pain trials: a systematic review and meta-regression. BMC Musculoskeletal Disorders. 2015; 16:370 10.1186/s12891-015-0825-6 26620449 PMC4663726

[20] Froud R. Improving interpretation of patient-reported outcomes in low back pain trials. PhD thesis. Queen Mary University of London; 2010.

[21] Eldridge SM, Ashby D, Feder GS, Rudnicka AR, Ukoumunne OC. Lessons for cluster randomized trials in the twenty-first century: A systematic review of trials in primary care. Clin Trials. 2004; 1:80–90. 10.1191/1740774504cn006rr 16281464

[22] Huskisson E. Measurement of Pain. The Lancet. 1974; (ii):1127 10.1016/S0140-6736(74)90884-8 4139420

[23] Roland M, Morris R. A study of the natural history of back pain. Part I: development of a reliable and sensitive measure of disability in low-back pain. Spine. 1983; 8(2):141–4. 10.1097/00007632-198303000-00004 6222486

[24] Fairbank JC, Couper J, Davies JB, O’Brien JP. The Oswestry low back pain disability questionnaire. Physiotherapy. 1980; 66(8):271–3. 6450426

[25] Downie WW, Leatham PA, Rhind VM, Wright V, Branco JA, Anderson JA. Studies with pain rating scales. Ann Rheum Dis. 1978; 37(4):378–81. 10.1136/ard.37.4.378 686873 PMC1000250

[26] Childs J, Piva S, Fritz J. Responsiveness of the Numeric Pain Rating Scale in Patients with Low Back Pain. Spine. 2005; 30(11). 10.1097/01.brs.0000164099.92112.29 15928561

[27] Beurskens A, de Vet H, Koke A. Responsiveness of functional status in low back pain: A comparison of different instruments. Pain. 1996; 65:71–76. 10.1016/0304-3959(95)00149-2 8826492

[28] Cieza L, Stucki G, Weigl M, Disler P, Jackel W, van der Linden S, et al ICF core sets for low back pain. J Rehabil Med. 2004; Suppl. 44:69–74. 10.1080/16501960410016037 15370751

[29] Mullis R, Barber J, Lewis M, Hay E. ICF core sets for low back pain: do they include what matters to patients? J Rehabil Med. 2007; 39(5): 353–357. 10.2340/16501977-0059 17549324

[30] Buchbinder R, Batterham R, Elsworth G, Dionne C, Irvin E, Osborne R. A validity-driven approach to the understanding of the personal and societal burden of low back pain: development of a conceptual and measurement model. Arthritis Research & Therapy. 2011; 13(5):R152 10.1186/ar3468 21933393 PMC3308082

[31] Hush JM, Kamper SJ, Stanton TR, Ostelo R, Refshauge KM. Standardized measurement of recovery from nonspecific back pain. Arch Phys Med Rehabil. 2012; 93(5):849–855. 10.1016/j.apmr.2011.11.035 22444028

[32] Froud R, Patterson S, Eldridge S, Seale C, Pincus T, Rajendran D, et al A systematic review and meta-synthesis of the impact of low back pain on people’s lives. BMC Musculoskelet Disord. 2014; 15:50 10.1186/1471-2474-15-50 24559519 PMC3932512

[33] Chiarotto A, Deyo RA, Terwee CB, Boers M, Buchbinder R, Corbin TP, et al Core outcome domains for clinical trials in non-specific low back pain. Eur Spine J. 2015; 24(6):1127–1142. 10.1007/s00586-015-3892-3 25841358

[34] Chiarotto A, Maxwell L, Terwee C, Wells G, Tugwell P, Ostelo R. Roland-Morris Disability Questionnaire and Oswestry Disability Index: Which Has Better Measurement Properties for Measuring Physical Functioning in Nonspecific Low Back Pain? Systematic Review and Meta-Analysis. Physical Therapy. 2016 10.2522/ptj.20150420 27081203

[35] Froud R, Abel G. Using ROC Curves to Choose Minimally Important Change Thresholds when Sensitivity and Specificity Are Valued Equally: The Forgotten Lesson of Pythagoras. Theoretical Considerations and an Example Application of Change in Health Status. PLoS One. 2014; 9(12):e114468 10.1371/journal.pone.0114468 25474472 PMC4256421

[36] de Vet HC, Terwee C, Knol DL, Bouter L. When to use agreement versus reliability measures. Clin Epidemiol. 2006; 59:1033–1039. 10.1016/j.jclinepi.2005.10.015 16980142

[37] Froud, R. How we currently measure back pain in O’Dowd FRCS Orth, J. and Hlavsova MSc, MCSP, HPC, A. (eds). Back Pain Management: Physical and Psychological Treatments, The Biomedical & Life Sciences Collection, Henry Stewart Talks Ltd, London (online at http://hstalks.com/?t=BL1984087).

[38] Mokkink LB, Terwee CB, Patrick DL, Alonso J, Stratford PW, Knol DL, et al The COSMIN checklist for assessing the methodological quality of studies on measurement properties of health status measurement instruments: an international Delphi study. Quality of Life Research. 2010; 19:539–549. 10.1007/s11136-010-9606-8 20169472 PMC2852520

[39] Williamson P, Gargon L, Clarke M, Blazeby J, Altman D. Standards for COS development. COMET Management Group 2013; http://www.comet-initiative.org/assets/downloads/Standards.pdf.

[40] Chan AW, Tetzlaff JM, Altman DG, Laupacis A, Gøtzsche PC, Krleza-Jerić K, et al SPIRIT 2013 statement: defining standard protocol items for clinical trials. Ann Intern Med. 2013; 158: 200–207. 10.7326/0003-4819-158-3-201302050-00583 23295957 PMC5114123

[41] Schulz KF, Altman DG, Moher D, CONSORT Group. CONSORT 2010 Statement: Updated Guidelines for Reporting Parallel Group Randomised Trials. PLoS Med. 2010; 7(3): e1000251 10.1371/journal.pmed.1000251 20352064 PMC2844794

[42] International Conference on Harmonisation Expert Working Group. Statistical principles for clinical trials E9. ICH tripartite guideline. 1998. 10532877

[43] Litcher-Kelly L, Martino S, Broderick J, Stone A: A Systematic Review of Measures Used to Assess Chronic Musculoskeletal Pain in Clinical and Randomized Controlled Clinical Trials. The Journal of Pain. 2007; 8(12):906–913. 10.1016/j.jpain.2007.06.009 17690014 PMC2691574

[44] Hayes M, Patterson D. Experimental development of the graphic rating method. Psychol Bull. 1921; 18(98).

[45] Araújo F, Cordeiro I, Ramiro S, Falzon L, Branco J, Buchbinder R. Outcomes assessed in trials of gout and accordance with OMERACT-proposed domains: a systematic literature review. Rheumatology. 2015; 54(6):981–93. 10.1093/rheumatology/keu424 25398382

[46] Page MJ, McKenzie JE, Green SE, Beaton DE, Jain NB, Lenza M, et al Core domain and outcome measurement sets for shoulder pain trials are needed: systematic review of physical therapy trials. J Clin Epidemiol. 2015; 68(11):1270–81. 10.1016/j.jclinepi.2015.06.006 26092288 PMC4711903

[47] Nuovo J, Melnikow J, Chang D. Reporting number needed to treat and absolute risk reduction in randomized controlled trials. Jama-Journal of the American Medical Association. 2002; 287(21):2813–2814. 10.1001/jama.287.21.2813 12038920

[48] Grotle M, Brox J, Vollestad N. Functional Status and Disability Questionnaires: What Do They Assess?: A Systematic Review of Back-Specific Outcome Questionnaires. Spine. 2004; 30:130–140. 10.1097/01.brs.0000149184.16509.73 15626993

[49] Costa L, Meyer C, Latimer J. Self-report outcome measures for low back pain. Spine. 2007; 32(9). 10.1097/01.brs.0000261024.27926.0f 17450079

[50] Kopec J. Measuring functional outcomes in persons with back pain. A review of back-specific questionnaires. Spine. 2000; 25(24):3110–3114. 10.1097/00007632-200012150-00005 11124726

[51] Castellini G, Gianola S, Bonovas S, Moja L. Improving Power and Sample Size Calculation in Rehabilitation Trial Reports: A Methodological Assessment. Arch Phys Med Rehabil. 2016; 97(7):1195–201. 10.1016/j.apmr.2016.02.013 26971671

