# Supplementary material for: A Systematic Review of Outcome Measures Use, Analytical Approaches, Reporting Methods, and Publication Volume by Year in Low Back Pain Trials Published between 1980 and 2012: Respice, adspice, et prospice
Source: PLoS One. 2016 Oct 24;11(10):e0164573. doi: 10.1371/journal.pone.0164573 (PMC5077121; doi:10.1371/journal.pone.0164573)
Supplement: S1 Text — A typical search strategy used in the systematic review. (PDF) [file pone.0164573.s001.pdf]

S1: Example search strategy

PubMed

#1 **(("2007/1/1"[Date - MeSH] : "3000"[Date - MeSH])) AND low back pain**
#2 **(("2007/1/1"[Date - MeSH] : "3000"[Date - MeSH])) AND back pain**
#3 low back pain OR lumbago OR backache
#4 (#1 OR #2 OR #3) Filters: **Clinical Trial; Publication date from 2007/1/01 to 2012/01/01**
