# Supplementary material for: A Systematic Review of Outcome Measures Use, Analytical Approaches, Reporting Methods, and Publication Volume by Year in Low Back Pain Trials Published between 1980 and 2012: Respice, adspice, et prospice
Source: PLoS One. 2016 Oct 24;11(10):e0164573. doi: 10.1371/journal.pone.0164573 (PMC5077121; doi:10.1371/journal.pone.0164573)
Supplement: S1 Table — A table showing the characteristics of included trials and their references. (PDF) [file pone.0164573.s002.pdf]

Included trial characteristics and references

Lear author	Year	Title	Primary outcome(s)	Sample size
Ackerman	2008	Pain relief with intraarticular or medial branch nerve blocks in patients with positive lumbar facet joint SPECT imaging: A 12-week outcome study	Numeric Rating Scale for Pain (NRS)	46
Agrifoglio	1994	Aceclofenac: A new NSAID in the treatment of acute lumbago. Multicentre single blind study vs diclofenac	Visual Analogue Scale for Pain (VAS-P)	90
Ahmed	2009	Evaluation of the effects of shortwave diathermy in patients with chronic low back pain	Lattinen's test score	97
Akbari	2008	The effect of motor control exercise versus general exercise on lumbar local stabilizing muscles thickness: Randomized controlled trial of patients with chronic low back pain	Muscle thickness measurement	49
Alaranta	1994	Intensive physical and psychosocial training program for patients with chronic low back pain. A controlled clinical trial	Million Visual Analogue Scale (MVAS)	378
Albaladejo	2010	The efficacy of a short education program and a short physiotherapy program for treating low back pain in primary care: a cluster randomized trial	Roland-Morris Disability Questionnaire (RMDQ)	348
Allan	2005	Transdermal fentanyl versus sustained release oral morphine in strong-opioid naive patients with chronic low back pain	VAS-P 100mm, constipation (bowel function assessment)	680
Altmaier	1992	The effectiveness of psychological interventions for the rehabilitation of low back pain: a randomized controlled trial evaluation	Low back pain rating scale (LBPRS)	45
Amlie	1987	Treatment of acute low-back pain with piroxicam: results of a double-blind placebo-controlled trial	Visual Analogue Scale for Pain (VAS-P)	266
Andersson	1999	A comparison of osteopathic spinal manipulation with standard care for patients with low back pain	Roland-Morris Disability Questionnaire (RMDQ)	155
Anema	2007	Multidisciplinary rehabilitation for subacute low back pain: graded activity or workplace intervention or both? A randomized controlled trial	Sick leave duration	112
Attanayake	2010	Clinical evaluation of selected yogic procedures in individuals with low back pain	Numeric Rating Scale for Pain (NRS)	12
Aure	2003	Manual therapy and exercise therapy in patients with chronic low back pain: a randomized, controlled trial with 1-year follow-up	VAS; ODI; Dartmouth COOP; return to work status.	49
Bakshi	1994	Treatment of acute lumbosacral back pain with diclofenac resinate: results of a double-blind trial versus piroxicam.	Visual Analogue Scale for Pain (VAS-P)	132
Balogh	2005	Effectiveness of balneotherapy in chronic low back pain -- a randomized single-blind controlled follow-up study	Visual Analogue Scale for Pain (VAS-P)	56
Bannwarth	2005	A randomized, double-blind, placebo controlled triphosphate in study of oral adenosine subacute low back pain	Roland-Morris Disability Questionnaire (RMDQ)	161
Baratta	1982	A double-blind study of cyclobenzaprine and placebo in the treatment of acute musculoskeletal conditions of the low back	Presence of muscle spasm (palpation)	117
Basford	1999	Laser therapy: a randomized, controlled trial of the effects of low-intensity Nd:YAG laser irradiation on musculoskeletal back pain	Perception of benefit, Oswestry Disability Index (ODI) , lumbar mobility (Schober's test)	56
Basler	1997	Incorporation of cognitive-behavioral treatment into the medical care of chronic low back patients: a controlled randomized study in German pain treatment centers	Pain diary (pain intensity, control over pain NRS 0-10, intake of pain medication)	76
Becker	2008	Effects of two guideline implementation strategies on patient outcomes in primary care: a cluster randomized controlled trial	The Hannover Functional Ability Questionnaire is a 12-item self-administered questionnaire	1261
Bekkering	2005	Implementation of clinical guidelines on physical therapy for patients with low back pain: Randomized trial comparing patient outcomes after a standard and active implementation strategy	Quebec back pain disability scale (QBPDS)	476
Bello	2010	Hydrotherapy versus land-based exercises in the management of chronic low back pain: A comparative study	Visual Analogue Scale for Pain (VAS-P)	12
Bendix	1997	Comparison of three intensive programs for chronic low back pain patients: a prospective, randomized, observer-blinded study with one-year follow-up	Work-ready rate	109
Bendix	1995	Active treatment programs for patients with chronic low back pain: a prospective, randomized, observer-blinded study	Return-to-work rate	106
BenSalahFrih	2009	Efficacy and treatment compliance of a home-based rehabilitation programme for chronic low back pain: a randomized, controlled study	Visual Analogue Scale for Pain (VAS-P)	107
Bentsen	1997	The effect of dynamic strength back exercise and/or a home training program in 57-year-old women with chronic low back pain. Results of a prospective randomized study with a 3-year follow-up period	The Nordic questionnaire	74
Bertalanffy	2005	Transcutaneous electrical nerve stimulation reduces acute low back pain during emergency transport	Pain and anxiety (VAS)	63
Berwick	1989	No clinical effect of back schools in an HMO. A randomized prospective trial	Visual Analogue Scale for Pain (VAS-P)	222
Beurskens	1995	Efficacy of traction for non-specific low back pain: a randomised clinical trial	Global perception of perceived effect (7-point scale (TQ)), severity of main complaints VAS	151
Bialosky	2009	Spinal manipulative therapy has an immediate effect on thermal pain sensitivity in people with low back pain: a randomized controlled trial	Numeric Rating Scale for Pain (NRS)	36
Bicalho	2010	Immediate effects of a high-velocity spine manipulation in paraspinal muscles activity of nonspecific chronic low-back pain subjects	EMG instrumentation	40
Birbara	2003	Treatment of chronic low back pain with etoricoxib, a new cyclo-oxygenase-2 selective inhibitor: improvement in pain and disability--a randomized, placebo-controlled, 3-month trial	Visual Analogue Scale for Pain (VAS-P)	212
Birkenmaier	2007	Medial branch blocks versus pericapsular blocks in selecting patients for percutaneous cryodenervation of lumbar facet joints	Visual Analogue Scale for Pain (VAS-P)	26
Bishop	2010	The Chiropractic Hospital-based Interventions Research Outcomes (CHIRO) study: a randomized controlled trial on the effectiveness of clinical practice guidelines in the medical and chiropractic management of patients with acute mechanical low back pain	Roland-Morris Disability Questionnaire (RMDQ)	72

Blomberg	1994	Manual therapy with steroid injections--a new approach to treatment of low back pain. A controlled multicenter trial with an evaluation by orthopedic surgeons	Quality of life VAS measurements for 24 variables	98
Blomberg	1992	A controlled, multicentre trial of manual therapy in low-back pain. Initial status, sick-leave and pain score during follow-up	Sick leave	101
Borman	2003	The efficacy of lumbar traction in the management of patients with low back pain.	Transition Question (TQ)	48
Brennan	2006	Identifying subgroups of patients with acute/subacute "nonspecific" low back pain: results of a randomized clinical trial	Oswestry Disability Index (ODI)	123
Bruce	2004	The impact of a moderated e-mail discussion group on use of complementary and alternative therapies in subjects with recurrent back pain	Complementary/alternative medicine use	421
Bruehl	2007	Trait Anger Expressiveness and Pain-Induced Beta-Endorphin Release: Support for the Opioid Dysfunction Hypothesis	Anger management style	27
Bruehl	2008	Anger Management Style and Emotional Reactivity to Noxious Stimuli Among Chronic Pain Patients and Healthy Controls: The Role of Endogenous Opioids	Anger management style	125
Brinkhaus	2006	Acupuncture in patients with chronic low back pain: a randomized controlled trial	Visual Analogue Scale for Pain (VAS-P)	284
Bronfort	1996	Trunk exercise combined with spinal manipulative or NSAID therapy for chronic low back pain: a randomized, observer-blinded clinical trial	Patient related low back pain (ordinal 11-box scale (NRS)), disability (RMDQ), functional status (Dartmouth primary care cooperative information project (COOP)) at 5 and 11 weeks	148
Brown	1986	Comparison of diflunisal and acetaminophen with codeine in the treatment of initial or recurrent acute low back strain	Graded scale for pain and disability	40
Buerger	1980	A controlled trial of rotational manipulation in low back pain.	Patient's impression of treatment effect (TQ)	53
Burton	1999	Information and Advice to Patients With Back Pain Can Have a Positive Effect	FABQ	126
Bush	1985	A controlled evaluation of paraspinal EMG biofeedback in the treatment of chronic low back pain	Pain (daily low back pain record (1-10 point scale (NRS)) duration in waking hours and resultant incapacity (1-5 point scale)	66
Cairns	2006	Randomized controlled trial of specific spinal stabilization exercises and conventional physiotherapy for recurrent low back pain	Roland-Morris Disability Questionnaire (RMDQ)	97
Cairns	2003	Level of distress in a recurrent low back pain population referred for physical therapy	Roland-Morris Disability Questionnaire (RMDQ)	212
Callaghan	1994	Evaluation of a back rehabilitation groupof chronic back pain in an outpatient setting	Lumbar movements (fluid goniometer)	80
Calmels	2009	Effectiveness of a lumbar belt in subacute low back pain: an open, multicentric, and randomized clinical study	EIFEL scale (French version of the Roland-Morris scale).	190
Carr	2005	A randomized trial comparing a group exercise programme for back pain patients with individual physiotherapy in a severely deprived area	Roland-Morris Disability Questionnaire (RMDQ)	203
Cecchi	2010	Spinal manipulation compared with back school and with individually delivered physiotherapy for the treatment of chronic low back pain: a randomized trial with one-year follow-up	Roland-Morris Disability Questionnaire (RMDQ)	205
Chan	2011	Aerobic exercise training in addition to conventional physiotherapy for chronic low back pain: a randomized controlled trial	VAS-P, Aberdeen low back pain disability questionnaire	43
Chang	2008	Effects of piroxicam-beta-cyclodextrin sachets on abnormal postural sway in patients with chronic low back pain	Visual Analogue Scale for Pain (VAS-P)	42
Chatchawan	2005	Effectiveness of traditional Thai massage versus Swedish massage among patients with back pain associated with myofascial trigger points	Visual Analogue Scale for Pain (VAS-P)	178
Chatzitheodorou	2008	The effect of exercise on adrenocortical responsiveness of patients with chronic low back pain, controlled for psychological strain	McGill Pain Questionnaire	61
Cheing	1999	Transcutaneous Electrical Nerve Stimulation: NonparallelAntinociceptive Effects on Chronic Clinical Pain and AcuteExperimental Pain	Visual Analogue Scale for Pain (VAS-P)	30
Cherkin	1996	Pitfalls of Patient Education: Limited Success of a Program for Back Pain in Primary Care	Perceived knowledge (5-point scale), Worry (0-10 point scale), Symptoms (0-10 point scale - bothersomeness of pain), Control (5-point likert), Evaluation of care (0-100 point scale), Exercise (days per week, duration of sessions), Function (RMDQ), Disability (National Health Interview Survey), health care use	201
Cherkin	1998	A Comparison of Physical Therapy, Chiropractic Manipulation, and Provision of an Educational Booklet for the Treatment of Patients with Low Back Pain	Bothersomeness during preceding 24 hours 11-point scale	321
Cherkin	2001	Randomized trial comparing traditional Chinese medical acupuncture, therapeutic massage, and self-care education for chronic low back pain	Bothersomeness of symptoms (0-10 point scale), RMDQ	250
Cherkin	2009	A randomized trial comparing acupuncture, simulated acupuncture, and usual care for chronic low back pain	Roland-Morris Disability Questionnaire (RMDQ)	606
Cherkin	2011	A comparison of the effects of 2 types of massage and usual care on chronic low back pain: a randomized, controlled trial	Roland-Morris Disability Questionnaire (RMDQ), Numerig Rating Scale (NRS)	380
Childs	2004	A clinical prediction rule to identify patients with low back pain most likely to benefit from spinal manipulation: a validation study	Oswestry Disability Index (ODI)	125
Chiradejnant	2003	Efficacy of "therapist-selected" versus "randomly selected" mobilisation techniques for the treatment of low back pain: a randomised controlled trial	Visual Analogue Scale for Pain (VAS-P)	140
Chiu	2011	The efficacy and safety of intramuscular injections of methylcobalamin in patients with chronic nonspecific low back pain: a randomised controlled trial	Oswestry Disability Index (ODI)	58
Chok	1999	Endurance training of the trunk extensor muscles in people with subacute low back pain	VAS-P, pain rating index of the McGill pain questionnaire	54
Chown	2008	A prospective study of patients with chronic back pain randomised to group exercise, physiotherapy or osteopathy	Oswestry Disability Index (ODI)	98
Chrubasik	2001	Treatment of low back pain with a herbal or synthetic anti-rheumatic	Modified Arthus index (mAI)	62

Cleland	2009	Comparison of the effectiveness of three manual physical therapy techniques in a subgroup of patients with low back pain who satisfy a clinical prediction rule: a randomized clinical trial	Oswestry Disability Index (ODI)	108
Coats	2004	Effects of valdecoxib in the treatment of chronic low back pain: results of a randomized, placebo-controlled trial	Visual Analogue Scale for Pain (VAS-P)	249
Costa	2009	Motor control exercise for chronic low back pain: a randomized placebo-controlled trial	Numeric Rating Scale (NRS)	154
Cramer	1993	The Hmax/Mmax ratio as an outcome measure for acute low back pain.	Hmax/Mmax	36
Cuesta-Vargas	2011	Exercise, manual therapy, and education with or without high-intensity deep-water running for nonspecific chronic low back pain: a pragmatic randomized controlled trial	Visual Analogue Scale for Pain (VAS-P)	46
Damush	2003	The long-term effects of a self-management program for inner-city primary care patients with acute low back pain	Functional status (Arthritis impact measurement scales (AIMS2), RMDQ	211
Dapas	1985	Baclofen for the treatment of acute low-back syndrome. A double-blind comparison with placebo	Local pain in lumbar area	195
Dechow	1999	A randomized, double-blind, placebo-controlled trial of sclerosing injections in patients with chronic low back pain	McGill pain questionnaire (short-form)	74
Defrin	2005	Conservative correction of leg-length discrepancies of 10mm or less for the relief of chronic low back pain	Visual Analogue Scale for Pain (VAS-P)	33
Demirel	2008	Effects of balneotherapy with exercise in patients with low back pain	Oswestry Disability Index (ODI)	44
Demoulin	2010	Effectiveness of a semi-intensive multidisciplinary outpatient rehabilitation program in chronic low back pain	Visual Analogue Scale for Pain (VAS-P)	160
Descarreaux	2002	Evaluation of a specific home exercise program for low back pain.	Visual Analogue Scale for Pain (VAS-P)	20
Dettori	1995	The effects of spinal flexion and extension exercises and their associated postures in patients with acute low back pain	Roland-Morris Disability Questionnaire (RMDQ)	180
Deyo	1986	How many days of bed rest for acute low back pain? A randomized clinical trial	Days of work lost	189
Deyo	1990	A controlled trial of transcutaneous electrical nerve stimulation (TENS) and exercise for chronic low back pain	Pain NRS, VAS-P	125
DiazArribas	2009	Effectiveness of the physical therapy Godelive Denys-Struyf method for nonspecific low back pain: primary care randomized control trial	Visual Analogue Scale for Pain (VAS-P)	126
DiCesare	2011	Comparison between the effects of trigger point mesotherapy versus acupuncture points mesotherapy in the treatment of chronic low back pain: a short term randomized controlled trial	VAS-P, VRS (Vermab rating scale)	61
Djais	2005	The role of lumbar spine radiography in the outcomes of patients with simple acute low back pain	Roland-Morris Disability Questionnaire (RMDQ)	76
Djavid	2007	In chronic low back pain, low level laser therapy combined with exercise is more beneficial than exercise alone in the long term: a randomised trial	Visual Analogue Scale for Pain (VAS-P)	53
Donchin	1990	Secondary prevention of low-back pain. A clinical trial	Low back pain episodes in year following treatment (Painful months in the 1-year period following treatment)	142
Donzelli	2006	Two different techniques in the rehabilitation treatment of low back pain: a randomized controlled trial	Oswestry Disability Index (ODI)	43
Dreiser	2003	Relief of acute low back pain with diclofenac-K 12.5 mg tablets: a flexible dose, ibuprofen 200 mg and placebo-controlled clinical trial	End of study global assessment (5-point) (TQ)	356
Dufour	2010	Treatment of chronic low back pain: a randomized, clinical trial comparing group-based multidisciplinary biopsychosocial rehabilitation and intensive individual therapist-assisted back muscle strengthening exercises	Visual Analogue Scale for Pain (VAS-P)	272
Dundar	2009	Clinical effectiveness of aquatic exercise to treat chronic low back pain: a randomized controlled trial	Spinal mobility (Schober test)	65
Durmus	2009	Effects of electrical stimulation program on trunk muscle strength, functional capacity, quality of life, and depression in the patients with low back pain: a randomized controlled trial	Muscle strength (Baseline Push-Pull Dyna-)	43
Durmus	2010	Effects of therapeutic ultrasound on pain, disability, walking performance, quality of life, and depression in patients with chronic low back pain: A randomized, placebo controlled trial	Visual Analogue Scale for Pain (VAS-P)	42
Durmus	2010	Effects of therapeutic ultrasound and electrical stimulation program on pain, trunk muscle strength, disability, walking performance, quality of life, and depression in patients with low back pain: a randomized-controlled trial	Visual Analogue Scale for Pain (VAS-P)	59
Elnaggar	1991	Effects of spinal flexion and extension exercises on low-back pain and spinal mobility in chronic mechanical low-back pain patients	McGill Pain questionnaire	56
Engbert	2011	The effects of therapeutic climbing in patients with chronic low back pain: a randomized controlled study	SF-36 (physical and mental)	23
Ergun	2010	The efficacy, safety, and pharmacokinetics of intramuscular and oral phenyramidol in patients with low back pain in an emergency department	Visual Analogue Scale for Pain (VAS-P)	72
Estlander	1991	Effects and follow-up of a multimodal treatment program including intensive physical training for low back pain patients	Spinal mobility	65
Ewert	2009	The comparative effectiveness of a multimodal program versus exercise alone for the secondary prevention of chronic low back pain and disability	German version of the WHYMPI (multidimensional pain inventory, or MPI-D)	183
Fairbank	2005	Randomised controlled trial to compare surgical stabilisation of the lumbar spine with an intensive rehabilitation programme for patients with chronic low back pain: the MRC spine stabilisation trial	Oswestry Disability Index (ODI), the shuttle walking test (speed and endurance)	321
Farhadi	2009	The effectiveness of wet-cupping for nonspecific low back pain in Iran: a randomized controlled trial	McGill Pain Questionnaire	98
Ferreira	2007	Comparison of general exercise, motor control exercise and spinal manipulative therapy for chronic low back pain: A randomized trial	Patient-Specific Functional Scale	224
Ferreira	2009	Relationship between spinal stiffness and outcome in patients with chronic low back pain	Global perceived effect (GPE (TQ))	191
Field	2007	Lower back pain and sleep disturbance are reduced following massage therapy	VITAS (VAS)	30

Fiore	2011	Short-term effects of high-intensity laser therapy versus ultrasound therapy in the treatment of low back pain: a randomized controlled trial	Visual Analogue Scale for Pain (VAS-P)	30
Fonseca	2009	Laboratory gait analysis in patients with low back pain before and after a pilates intervention	Vertical ground-reaction force (VGRF)	28
Franca	2010	Segmental stabilization and muscular strengthening in chronic low back pain: a comparative study	Visual Analogue Scale for Pain (VAS-P)	30
Frerick	2003	Topical treatment of chronic low back pain with a capsicum plaster	Arthus low back pain rating scale, reduction in pain (11-point likert)	319
Friedrich	2009	Long-term effects of a combined exercise and motivation program in patients with chronic low back pain: A five-year follow-up	Disability (low back outcome scale questionnaire developed by Greenough and Fras	74
Friedrich	2005	Long-term effect of a combined exercise and motivational program on the level of disability of patients with chronic low back pain	Low Back Outcome Score (LBOS), Pain (101 point NRS and pain drawing), working ability	74
Fritzell	2001	Lumbar fusion versus non-surgical treatment for chronic low back pain. A multicenter randomized controlled trial from the swedish lumbar spine study	VAS-P, back-to-work (binary), disability (Oswestry disability index, Million VAS, general function score), global self reporting	289
Frost	2004	Randomised controlled trial of physiotherapy compared with advice for low back pain.	Oswestry Disability Index (ODI)	200
Frost	1995	Randomised controlled trial for evaluation of fitness programme for patients with chronic low back pain	Oswestry Disability Index (ODI)	71
Fuchs	2005	Intraarticular hyaluronic acid versus glucocorticoid injections for nonradicular pain in the lumbar spine	Visual Analogue Scale for Pain (VAS-P)	60
Garvey	1989	A prospective, randomized, double-blind evaluation of trigger-point injection therapy for low-back pain	Numeric Rating Scale for Pain (NRS)	63
Gatchel	2003	Treatment- and cost-effectiveness of early intervention for acute low-back pain patients: a one-year prospective study	Characteristic Pain Inventory	70
Geisser	2005	A randomized, controlled trial of manual therapy and specific adjuvant exercise for chronic low back pain	Visual Analogue Scale for Pain (VAS-P)	100
George	2003	The effect of a fear-avoidance-based physical therapy intervention for patients with acute low back pain: results of a randomized clinical trial	Oswestry Disability Index (ODI)	66
George	2006	Sex differences in predictors of outcome in selected physical therapy interventions for acute low back pain.	Numeric Rating Scale for Pain (NRS)	165
George	2005	Distinguishing patient satisfaction with treatment delivery from treatment effect: a preliminary investigation of patient satisfaction with symptoms after physical therapy treatment of low back pain	Patient satisfaction NASS outcome assessment (3 items from the north American spine society lumbar spine outcome assessment instrument)	58
Gibson	1985	Controlled comparison of short-wave diathermy treatment with osteopathic treatment in non-specific low back pain	Clinical examination (including XR)	101
Gilbert	2004	Low Back Pain: Influence ofEarly MR Imaging or CT onTreatment and Outcome?Multicenter RandomizedTrial	Aberdeen Low Back Disability Scale (LBPDS)	648
Gladwell	2006	Does a program of Pilates improve chronic non-specific low back pain?	Roland Disability Questionnaire (RMDQ)	34
Glazov	2009	Laser acupuncture for chronic non-specific low back pain: a controlled clinical trial	Visual Analogue Scale for Pain (VAS-P)	90
Goehner	2006	Preventing chronic back pain: evaluation of a theory-based cognitive-behavioural training programme for patients with subacute back pain	Likert scale for self-efficacy	47
Goldby	2006	A randomized controlled trial investigating the efficiency of musculoskeletal physiotherapy on chronic low back disorder	Numeric Rating Scale for Pain (NRS)	200
Goldstein	2002	The impact of treatment confidence on pain and related disability among patients with low-back pain: results from the University of California, Los Angeles, low-back pain study	Treatment confidence (10-point)	650
Grant	1999	A randomized comparative trial of acupuncture versus transcutaneous electrical nerve stimulation for chronic back pain in the elderly	Visual Analogue Scale for Pain (VAS-P)	118
Gudavalli	2006	A randomized clinical trial and subgroup analysis to compare flexion-distraction with active exercise for chronic low back pain	VAS-P, RMDQ, SF-36	235
Gunn	1980	Dry needling of muscle motor points for chronic low-back pain: a randomized clinical trial with long-term follow-up	Status at discharge (pain and work status - 4-point scale)	56
Gur	2003	Efficacy of low power laser therapy and exercise on pain and functions in chronic low back pain	Visual Analogue Scale for Pain (VAS-P)	75
Guthrie	2011	The Effect of Traditional Bridging or Suspension Exercise Bridging on Lateral Abdominal Thickness in Individuals with Low Back Pain	Muscle thickness of external oblique, internal oblique and transversus abdominis	51
Haake	2007	German Acupuncture Trials (GERAC) for chronic low back pain: randomized, multicenter, blinded, parallel-group trial with 3 groups	von Korff Chronic Pain Grade; Hanover Functional Ability Questionnaire	1117
Haas	2005	Chronic disease self-management program for low back pain in the elderly	Modified Von Korff pain and disability scales (MvK)	101
Hackett	1988	Electroacupuncture compared with paracetamol for acute low back pain	Visual Analogue Scale for Pain (VAS-P)	37
Hadler	1987	A benefit of spinal manipulation as adjunctive therapy for acute low-back pain: a stratified controlled trial	Roland-Morris Disability Questionnaire (RMDQ)	54
Hagen	2010	Adding a physical exercise programme to brief intervention for low back pain patients did not increase return to work	Visual Analogue Scale for Pain (VAS-P)	246
Hall	2011	Tai chi exercise for treatment of pain and disability in people with persistent low back pain: a randomized controlled trial	NRS for bothersomeness	160
Hancock	2007	Assessment of diclofenac or spinal manipulative therapy, or both, in addition to recommended first-line treatment for acute low back pain: a randomised controlled trial	Number of days to recovery (measured in pain)	235
Hansen	1993	Intensive, dynamic back-muscle exercises, conventional physiotherapy, or placebo-control treatment of low-back pain. A randomized, observer-blind trial	Changes in pain level (visual interval scale 0-9 (NRS)	150
Harkapaa	1989	A controlled study on the outcome of inpatient and outpatient treatment of low back pain. Part I. Pain, disability, compliance, and reported treatment benefits three months after treatment	Pain severity (The pain index 4x VAS-P) - in general, in the morning, after work day and in the evening), LBP disability index	459
Harts	2008	A high-intensity lumbar extensor strengthening program is little better than a low-intensity program or a waiting list control group for chronic low back pain: a randomised clinical trial	Global Perceived Effect (GPE (TQ)); Roland-Morris Disability Questionnaire (RMDQ)	59

Hartvigsen	2005	Intensive education combined with low tech ergonomic intervention does not prevent low back pain in nurses	Standardised Nordic questionnaire	255
Hartvigsen	2010	Supervised and non-supervised Nordic walking in the treatment of chronic low back pain: a single blind randomized clinical trial	Low back pain rating scale (LBPRS)	136
Hasson	2004	A Randomized Clinical Trial of the Treatment Effects of Massage Compared to Relaxation Tape Recordings on Diffuse Long-Term Pain	Self related health (5-point) (TQ), Mental Energy Scale, muscle pain (3 or 4 point response to items for each measure)	88
Hawk	2005	A randomized trial investigating a chiropractic manual placebo: a novel design using standardized forces in the delivery of active and control treatments	Pain Disability Index (PDI)	106
Hay	2005	Comparison of physical treatments versus a brief pain-management programme for back pain in primary care: a randomised clinical trial in physiotherapy practice	Roland-Morris Disability Questionnaire (RMDQ)	319
Hazard	2000	A Controlled Trial of an Educational Pamphlet toPrevent Disability After Occupational Low Back Injury	Disability (percentage not working), lost work days	450
Helliwell	1987	Manipulation in low-back pain	"Standard Questions" about symptoms	14
Hemmila	2002	Long-term effectiveness of bone-setting, light exercise therapy, and physiotherapy for prolonged back pain: a randomized controlled trial	Oswestry Disability Index (ODI)	113
Henchoz	2010	Role of physical exercise in low back pain rehabilitation: a randomized controlled trial of a three-month exercise program in patients who have completed multidisciplinary rehabilitation	Oswestry Disability Index (ODI)	103
Henchoz2	2010	Functional multidisciplinary rehabilitation versus outpatient physiotherapy for non specific low back pain: randomized controlled trial	Oswestry Disability Index (ODI)	92
Herman	1994	A randomized controlled trial of transcutaneous electrical nerve stimulation (CODETRON) to determine its benefits in a rehabilitation program for acute occupational low back pain	Roland-Morris Disability Questionnaire (RMDQ)	58
Hernandez-Reif	2001	Lower back pain is reduced and range of motion increased after massage therapy.	McGill Pain Questionnaire (MPQ)	24
HertzmanMiller	2002	Comparing the satisfaction of low back pain patients randomized to receive medical or chiropractic care: results from the UCLA low-back pain study	Satisfaction with treatment provider (10 items)	672
Heymans	2006	The effectiveness of high-intensity versus low-intensity back schools in an occupational setting: a pragmatic randomized controlled trial	Return-to-work	299
Hickey	1982	Chronic low back pain: a comparison of diflunisal with paracetamol	Subjective and objective evaluation of clinical and physical signs: 0 - 3 scale of severity for LBP (NRS) , radiating pain, functional disability and limitation/pain on extension (scale)	30
Hoehler	1981	Spinal manipulation for low back pain	Questionnaires measuring pain, improvement in walking, bending, sitting, reaching, and dressing	95
Hoiriis	2004	A randomized clinical trial comparing chiropractic adjustments to muscle relaxants for subacute low back pain	VAS-P, Oswestry Disability Index (ODI), Zung self rating for depression scale, Schober's test	110
Hondras	2009	A randomized controlled trial comparing 2 types of spinal manipulation and minimal conservative medical care for adults 55 years and older with subacute or chronic low back pain	Roland-Morris Disability Questionnaire (RMDQ)	213
Hosie	1993	The topical NSAID, felbinac, versus oral ibuprofen: a comparison of efficacy in the treatment of acute lower back injury	Global assessment of condition (patient (TQ) and practitioner rated)	261
Hsieh	2002	Effectiveness of four conservative treatments for subacute low back pain: a randomized clinical trial.	Visual Analogue Scale for Pain (VAS-P)	184
Hsieh	2006	Treatment of low back pain by acupressure and physical therapy: randomised controlled trial	Roland-Morris Disability Questionnaire (RMDQ) (Chinese)	110
Hsieh	2004	A randomized controlled clinical trial for low back pain treated by acupressure and physical therapy	Short Form McGill pain questionnaire (Chinese)	146
Hsieh	2002	One-Shot Percutaneous ElectricalNerve Stimulation vs.Transcutaneous Electrical NerveStimulation for Low Back Pain	Visual Analogue Scale for Pain (VAS-P)	133
Hurley	2005	A descriptive study of the usage of spinal manipulative therapy techniques within a randomized clinical trial in acute low back pain	Roland-Morris Disability Questionnaire (RMDQ)	146
Hurley	2004	A randomized clinical trial of manipulative therapy and interferential therapy for acute low back pain	Roland-Morris Disability Questionnaire (RMDQ)	194
Hurri	1989	The Swedish back school in chronic low back pain. Part II. Factors predicting the outcome	Visual Analogue Scale for Pain (VAS-P)	185
Hurwitz	2002	A randomized trial of medical care with and without physical therapy and chiropractic care with and without physical modalities for patients with low back pain: 6-month follow-up outcomes from the UCLA low back pain study	Average and most severe low back pain intensity (10 point rating scale (NRS)), disability (RMDQ)	681
Iles	2011	Telephone coaching can increase activity levels for people with non-chronic low back pain: a randomised trial	Patient Specific Functional Scale	26
Inoue	2006	Relief of low back pain immediately after acupuncture treatment--a randomised, placebo controlled trial	Visual Analogue Scale for Pain (VAS-P)	31
Itoh	2004	Trigger point acupuncture treatment of chronic low back pain in elderly patients--a blinded RCT	Visual Analogue Scale for Pain (VAS-P)	35
Jarvik	2003	Rapid magnetic Resonance imaging vs radiography for patients with low back pain	Roland-Morris Disability Questionnaire (RMDQ)	337
Jellema	2005	Should treatment of (sub)acute low back pain be aimed at psychosocial prognostic factors? Cluster randomised clinical trial in general practice	Roland-Morris Disability Questionnaire (RMDQ), perceived recovery (seven point) (TQ), sick leave because of low back pain	307
Johannsen	1995	Exercises for chronic low back pain: a clinical trial.	Isokinetic back strength	27
Johnson	2007	Active exercise, education, and cognitive behavioral therapy for persistent disabling low back pain: a randomized controlled trial	VAS-P; Roland-Morris Disability Questionnaire (RMDQ)	223
Jousset	2004	Effects of functional restoration versus 3 hours per week physical therapy: a randomized controlled study	Days of sick leave between end of program and 6 month follow-up	82
Ju	2001	(211) A Placebo-Controlled Trial of Rofecoxib in the Treatment of Chronic Low Back Pain	Visual Analogue Scale for Pain (VAS-P)	380
Juni	2009	A randomised controlled trial of spinal manipulative therapy in acute low back pain	11-point box scale (BS-11) (NRS)	104

Kankaanpaa	1999	The efficacy of active rehabilitation in chronic low back pain. Effect on pain intensity, self-experienced disability, and lumbar fatigability	Pain disability index (PDI)	54
Kapitza	2010	First non-contingent respiratory biofeedback placebo versus contingent biofeedback in patients with chronic low back pain: a randomized, controlled, double-blind trial	Relaxation Index (RI)	42
Katz	2003	Efficacy and safety of rofecoxib in patients with chronic low back pain: results from two 4-week, randomized, placebo-controlled, parallel-group, double-blind trials	Visual Analogue Scale for Pain (VAS-P)	580
Keel	1998	Effectiveness of in-patient rehabilitation for sub-chronic and chronic low back pain by an integrative group treatment program (Swiss Multicentre Study)	Return-to-work	254
Keijsers	1990	The efficacy of the back school: a randomized trial.	Visual Analogue Scale for Pain (VAS-P)	77
Keitel	2001	Capsicum pain plaster in chronic non-specific low back pain	Arthus Low Back Rating Scale (various dimensions of pain with a VAS, impairment of movement and disability)	150
Kell	2009	A comparison of two forms of periodized exercise rehabilitation programs in the management of chronic nonspecific low-back pain	Strength Measures	240
Kell	2011	The response of persons with chronic nonspecific low back pain to three different volumes of periodized musculoskeletal rehabilitation	Biering-Sorensen Back Endurance (BSBE) test	27
Keller	1997	Multidisciplinary rehabilitation for chronic back pain in an outpatient setting: a controlled randomized trial	Pain frequency (5-point likert)	64
Kellett	1991	Effects of an exercise program on sick leave due to back pain	Short-term sick leave attributable to back pain	85
Kendrick	2001	Radiography of the lumbar spine in primary care patientswith low back pain: randomised controlled trial	Roland-Morris Disability Questionnaire (RMDQ)	402
Kerr	2003	Acupuncture in the management of chronic low back pain: a blinded randomized controlled trial	SF-36	60
Kerry	2000	Routine referral for radiography of patients presenting with low back pain: is patients? outcome influenced by GPs? referral for plain radiography?	Roland-Morris Disability Questionnaire (RMDQ), hospital anxiety and depression, EuroQol, SF-36	126
Ketenci	2005	Assessment of efficacy and psychomotor performances of thiocolchicoside and tizanidine in patients with acute low back pain	Global efficacy (4-point) (TQ)	97
Klein	1993	A randomized double-blind trial of dextrose-glycerine-phenol injections for chronic, low back pain.	Roland-Morris Disability Questionnaire (RMDQ)	79
Kofotolis	2006	Effects of two 4-week proprioceptive neuromuscular facilitation programs on muscle endurance, flexibility, and functional performance in women with chronic low back pain	Muscle endurance and lumbar mobility	86
Kolda	2008	Comparison of three different approaches in the treatment of chronic low back pain	LROM -Schober test	55
Kole-Snijders	1999	Chronic low-back pain: what does cognitive coping skills training add to operant behavioral treatment? Results of a randomized clinical trial	Activity tolerance	133
Kool	2005	Increasing days at work using function-centered rehabilitation in nonacute nonspecific low back pain: a randomized controlled trial	Number of days at work in 3 months after treatment	171
Kool	2007	Function-Centred Rehabilitation Increases Work Days in Patients With Nonacute Nonspecific Low Back Pain: 1-Year Results From a Randomized Controlled Trial	The number of calendar work days in the follow-up year	174
Koumantakis	2005	Trunk muscle stabilization training plus general exercise versus general exercise only: randomized controlled trial of patients with recurrent low back pain	Short form McGill Pain questionnaire	45
Koumantakis	2005	Supplementation of general endurance exercise with stabilisation training versus general exercise only. Physiological and functional outcomes of a randomised controlled trial of patients with recurrent low back pain	Short form McGill Pain questionnaire	55
Kovacs	2003	Effect of firmness of mattress on chronic non-specific low-backpain: randomised, double-blind, controlled, multicentre trial	VAS-P (in bed and on rising)	310
Kovacs	1997	Local and remote sustained trigger point therapy for exacerbations of chronic low back pain. A randomized, double-blind, controlled, multicenter trial	Visual Analogue Scale for Pain (VAS-P)	71
Kroll	2008	A randomized, double-blind, prospective study comparing the efficacy of continuous versus pulsed radiofrequency in the treatment of lumbar facet syndrome	Visual Analogue Scale for Pain (VAS-P)	26
Kulisch	2009	Effect of thermal water and adjunctive electrotherapy on chronic low back pain: a double-blind, randomized, follow-up study	Visual Analogue Scale for Pain (VAS-P)	71
Kumar	2009	Efficacy of two multimodal treatments on physical strength of occupationally subgrouped male with low back pain	Visual Analogue Scale for Pain (VAS-P)	141
Kumar	2010	Comparative efficacy of two multimodal treatments on male and female sub-groups with low back pain (part II)	Visual Analogue Scale for Pain (VAS-P)	102
Kumar	2009	Efficacy of dynamic muscular stabilization techniques (DMST) over conventional techniques in rehabilitation of chronic low back pain	Waddle functional evaluation test	30
Kuukkanen	2000	Effects of a three-month therapeutic exercise programme on flexibility in subjects with low back pain	Lumbar flexion (extension, side bending and rotation), muscle flexibility measurements	74
Lalanne	2009	Modulation of the flexion-relaxation response by spinal manipulative therapy: a control group study	Surface EMG of erector spinae at L2 and L5	27
Lamb	2010	Group cognitive behavioural treatment for low-back pain in primary care: a randomised controlled trial and cost-effectiveness analysis	Roland-Morris Disability Questionnaire (RMDQ), modified von Korff scores	598
Lambeek	2010	Randomised controlled trial of integrated care to reduce disability from chronic low back pain in working and private life	Return-to-work	134
Lankhorst	1983	The effect of the Swedish Back School in chronic idiopathic low back pain. A prospective controlled study.	Jan van Breemen Institute set of measurement for the quantification of LBP (six pain aspects each measured on NRS giving mean pain score, and degree of disability over last week also on NRS, and spinal mobility)	43
Lau	2008	Early physiotherapy intervention in an Accident and Emergency Department reduces pain and improves satisfaction for patients with acute low back pain: a randomised trial	Numeric Rating Scale for Pain (NRS)	102
Leclaire	2001	Radiofrequency facet joint denervation in the treatment of low back pain: a placebo-controlled clinical trial to assess efficacy	Roland-Morris Disability Questionnaire (RMDQ)	70

Leclaire	1996	Back School in a First Episode of Compensated Acute LowBack Pain: A Clinical Trial to Assess Efficacy and PreventRelapse	Time off work, recurrences	168
Leeuw	2008	Exposure in vivo versus operant graded activity in chronic low back pain patients: Results of a randomized controlled trial	Quebeck Back Pain Disability Scale (QBPDS) and patient specific complaints	85
Leibing	2002	Acupuncture treatment of chronic low-back pain -- a randomized, blinded, placebo-controlled trial with 9-month follow-up	VAS-P, Pain Disability Index (PDI)	131
Lengsfeld	2007	Passive rotary dynamic sitting at the workplace by office-workers with lumbar pain: a randomized multicenter study	Oswestry Disability Index (ODI) (German)	231
Leon	2008	TTM-based motivational counselling does not increase physical activity of low back pain patients in a primary care setting?A cluster-randomized controlled trial	Freiburger Questionnaire on Physical Activity	1261
Lewis	2011	Strain-Counterstrain therapy combined with exercise is not more effective than exercise alone on pain and disability in people with acute low back pain: a randomised trial	Oswestry Disability Index (ODI)	85
Lewis	2005	A randomized clinical trial comparing two physiotherapy interventions for chronic low back pain	Quebec Back Pain Disability Scale (QBPDS)	62
Licciardone	2003	Osteopathic manipulative treatment for chronic low back pain: a randomized controlled trial	SF-36, VAS-P, RMDQ, lost work or school days due to back pain	85
Lindstrom	1992	The effect of graded activity on patients with subacute low back pain: a randomized prospective clinical study with an operant-conditioning behavioral approach	Return-to-work	103
Lindstrom	1995	Physical performance, pain, pain behavior and subjective disability in patients with subacute low back pain	Perceived pain (Borg, 10-point scale)	98
Linton	1989	The secondary prevention of low back pain: a controlled study with follow-up	Visual Analogue Scale for Pain (VAS-P)	66
Little	2008	Randomised controlled trial of Alexander technique lessons, exercise, and massage (ATEAM) for chronic and recurrent back pain	Roland Disability Questionnaire (RMDQ)	463
Little	2001	Should we give detailed advice and information booklets to patients with back pain? A randomized controlled factorial trial of a self-management booklet and doctor advice to take exercise for back pain	Pain / function score (0-100, phone call)	239
Long	2004	Does it matter which exercise? A randomized control trial of exercise for low back pain	Pain intensity VAS-P, RMDQ, medication use	201
Lorig	2002	Can a Back Pain E-mail Discussion Group improve health status and lower health care costs?: A randomized study	Pain (visual numeric scale, a variation of a VAS), disability (RMDQ), role function(Illness intrusiveness scale), health distress (a short scale developed for the medical outcomes study) and health care utilization	454
MacDonald	1990	An open controlled assessment of osteopathic manipulation in nonspecific low-back pain	A disability index (12-point)	95
Machado	2010	The effectiveness of the McKenzie method in addition to first-line care for acute low back pain: a randomized controlled trial	Numeric Rating Scale for Pain (NRS)	139
Mackawan	2007	Effects of traditional Thai massage versus joint mobilization on substance P and pain perception in patients with non-specific low back pain	Substance P levels	68
Magnussen	2007	Motivating disability pensioners with back pain to return to work - A randomized controlled trial	Return-to-work defined as reduced disability pension payment	89
Manchikanti	2005	[ISRCTN 16558617] A randomized, controlled trial of spinal endoscopic adhesiolysis in chronic refractory low back and lower extremity pain	Visual Analogue Scale for Pain (VAS-P)	81
Manchikanti	2004	One day lumbar epidural adhesiolysis and hypertonic saline neurolysis in treatment of chronic low back pain: a randomized, double-blind trial	Visual Analogue Scale for Pain (VAS-P)	75
Manniche	1988	Clinical trial of intensive muscle training for chronic low back pain	Current low back pain (10-point scale (NRS))	90
Mannion	1999	A randomized clinical trial of three active therapies for chronic low back pain	Visual Analogue Scale for Pain (VAS-P)	137
Marchand	1993	Is TENS purely a placebo effect? A controlled study on chronic low back pain	Visual Analogue Scale for Pain (VAS-P)	42
Marks	1992	Facet joint injection and facet nerve block: a randomised comparison in 86 patients with chronic low back pain	Pain relief (4-point scale)	86
Marshall	2008	Self-report measures best explain changes in disability compared with physical measures after exercise rehabilitation for chronic low back pain.	McGill Pain Questionnaire (SF-MPQ)	50
Marshall	2008	Muscle activation changes after exercise rehabilitation for chronic low back pain.	Oswestry Disability Index (ODI)	50
Mathews	1988	Manipulation and traction for lumbago and sciatica: Physiotherapeutic techniques used in two controlled trials.	Pain (6-point score - referred to as a pain scale but it evidence it was improvement of symptoms: 6=symptom free, 5=much better, 4=slightly better, 3=the same, 2=slightly worse, 1=much worse)	53
Maul	2005	Long-term effects of supervised physical training in secondary prevention of low back pain	Functional ability, clinical investigation (height, weight, BP, ES tone, SLR), lifting capacity, trunk strength, range of motion, isometric muscle endurance, aerobic capacity	148
Mayer	2005	Treating acute low back pain with continuous low-level heat wrap therapy and/or exercise: a randomized controlled trial	Multidimensional Task Ability Profile Questionnaire	100
Mayer	2004	A randomized clinical trial of treatment for lumbar segmental rigidity	Roland-Morris Disability Questionnaire (RMDQ)	70
Mazza	2010	Escitalopram 20 mg versus duloxetine 60 mg for the treatment of chronic low back pain	Pain (described as Likert scale but coded as NRS as 1 to 10 point scale from no pain to worst pain imaginable)	75
Meade	1990	Low back pain of mechanical origin: randomised comparison of chiropractic and hospital outpatient treatment.	Oswestry Disability Index (ODI)	608
Melancon	2005	Massage therapy versus traditional therapy for low back pain relief: implications for holistic nursing practice	Oswestry Disability Index (ODI)	60
Melzack	1983	Transcutaneous electrical nerve stimulation for low back pain. A comparison of TENS and massage for pain and range of motion	McGill pain questionnaire	41

Meng	2011	Intermediate and Long-term Effects of a Standardized Back School for Inpatient Orthopedic Rehabilitation on Illness Knowledge and Self-management Behaviors	Illness knowledge	344
Mibielli	2009	Diclofenac plus B vitamins versus diclofenac monotherapy in lumbago: the DOLOR study	Visual Analogue Scale for Pain (VAS-P)	372
Middleton	1984	A comparison of two analgesic muscle relaxant combinations in acute back pain	Physician assessment of pain	107
Miller	2002	Cost-effectiveness of lumbar spine radiography in primary care patients with low back pain	Roland-Morris Disability Questionnaire (RMDQ)	89
Million	1981	Evaluation of low back pain and assessment of lumbar corsets with and without back supports	Subjective variables asked on back pain questionnaire for visual analogue assessment	19
Moffett	1986	A controlled, prospective study to evaluate the effectiveness of a back school in the relief of chronic low back pain.	Visual Analogue Scale for Pain (VAS-P)	78
Moffett	1999	Randomised controlled trial of exercise for low back pain: clinical outcomes, costs, and preferences	RMDQ, Aberdeen Back Pain Scale	179
Mohseni-Bandpei	2006	A prospective randomised controlled trial of spinal manipulation and ultrasound in the treatment of chronic low back pain	Visual Analogue Scale for Pain (VAS-P)	112
Mohseni-Bandpei	2011	The effect of pelvic floor muscle exercise on women with chronic non-specific low back pain	Visual Analogue Scale for Pain (VAS-P)	20
Molsberger	2002	Does acupuncture improve the orthopedic management of chronic low back pain: A randomized, blinded, controlled trial with 3 months follow up	Visual Analogue Scale for Pain (VAS-P)	124
Moore	2000	A randomized trial of a cognitive-behavioral program for enhancing back pain self care in a primary care setting	Back pain worry (10-point scale)	218
Morone	2011	Quality of life improved by multidisciplinary back school program in patients with chronic non-specific low back pain: a single blind randomized controlled trial	SF-36	70
Morrison	1988	Back pain: treatment and prevention in a community hospital	Physical strength (point scale)	54
Moseley	2003	Joining forces - combining cognition-targeted motor control training with group or individual pain physiology education: a successful treatment for chronic low back pain	Roland-Morris Disability Questionnaire (RMDQ)	41
Moseley	2004	A randomized controlled trial of intensive neurophysiology education in chronic low back pain	Survey of pain attitudes	54
Moseley	2002	Combined physiotherapy and education is efficacious for chronic low back pain	Roland-Morris Disability Questionnaire (RMDQ)	57
Muehlbacher	2006	Topiramate in treatment of patients with chronic low back pain: a randomized, double-blind, placebo-controlled study	Pain rating index of the McGill Pain Questionnaire, Stait-trait Anger Expression	96
Muller-Schwefe	2011	Dysport(registered trademark) for the treatment of myofascial back pain: Results from an open-label, Phase II, randomized, multicenter, dose-ranging study	Five-category verbal rating scale (referred to as NRS but not coded as such as verbal)	181
Muthukrishnan	2010	The differential effects of core stabilization exercise regime and conventional physiotherapy regime on postural control parameters during perturbation in patients with movement and control impairment chronic low back pain	Roland-Morris Disability Questionnaire (RMDQ)	30
Nadler	2003	Continuous low-level heatwrap therapy for treating acute nonspecific low back pain.	One-day pain relief (verbal response scale, 6-point)	208
Nadler	2002	Continuous low-level heat wrap therapy provides more efficacy than Ibuprofen and acetaminophen for acute low back pain.	Pain relief (6-point verbal rating scale)	354
Nadler	2003	Overnight use of continuous low-level heatwrap therapy for relief of low back pain.	Morning pain relief (5-point verbal response scale)	70
Nassif	2011	Evaluation of a randomized controlled trial in the management of chronic lower back pain in a French automotive industry: An observational study	Numeric Rating Scale for Pain (NRS)	60
Newcomer	2008	Is a videotape to change beliefs and behaviors superior to a standard videotape in acute low back pain? A randomized controlled trial	Oswestry Disability Index (ODI)	111
Newton-John	1995	Cognitive-behavioural therapy versus EMG biofeedback in the treatment of chronic low back pain.	Pain diary with 10-point scale (NRS)	44
Nicholas	1991	Operant-behavioural and cognitive-behavioural treatment for chronic low back pain	Pain rating chart (NRS 6-point scale)	58
Niemisto	2003	A randomized trial of combined manipulation, stabilizing exercises, and physician consultation compared to physician consultation alone for chronic low back pain	Pain intensity (VAS-P), frequency of LBP experienced, Oswestry (ODI)	200
Niemistoe	2005	Cost-effectiveness of combined manipulation, stabilizing exercises, and physician consultation compared to physician consultation alone for chronic low back pain: A prospective randomized trial with 2-year follow-up	Visual Analogue Scale for Pain (VAS-P)	201
Nigg	2009	The effectiveness of an unstable sandal on low back pain and golf performance	Visual Analogue Scale for Pain (VAS-P)	37
Nordeman	2006	Early access to physical therapy treatment for subacute low back pain in primary health care: a prospective randomized clinical trial	Borg category scale for rating of perceived pain (BRPP)	60
Norris	2008	The role of an integrated back stability program in patients with chronic low back pain	Sort from McGill Pain Questionnaire (SF-MPQ)	59
Nouwen	1983	EMG biofeedback used to reduce standing levels of paraspinal muscle tension in chronic low back pain.	Pain reports (daily log; 5 pt scale including severe, moderate, no pain labels but no labels on three of the 5 points)	20
Oesch	2006	The influence of a functional capacity evaluation on fitness for work certificates in patients with non-specific chronic low back pain	Functional capacity evaluation	174
Oleske	2007	Are back supports plus education more effective than education alone in promoting recovery from low back pain?: Results from a randomized clinical trial	Numeric Rating Scale (NRS)	433
Olson	1991	An analysis of the effectiveness of a low back health education program in an employee population.	Beck Behaviour Inventory (personal behaviour change)	37
Ongley	1987	A new approach to the treatment of chronic low back pain	Roland-Morris Disability Questionnaire (RMDQ), 9 questions from the Chronic Disability Index	81
Orava	1986	Medical treatment of acute low back pain. Diflunisal compared with indomethacin in acute lumbago	Pain at rest	130

Pach	2011	Efficacy of injections with Disci/Rhus toxicodendron compositum for chronic low back pain - a randomized placebo-controlled trial	Visual Analogue Scale for Pain (VAS-P)	142
Palangio	2002	Combination hydrocodone and ibuprofen versus combination oxycodone and acetaminophen in the treatment of moderate or severe acute low back pain.	Daily mean pain relief score (patient diary with numeric coded scale (NRS))	147
Pallay	2004	Etoricoxib reduced pain and disability and improved quality of life in patients with chronic low back pain: a 3 month, randomized, controlled trial	Visual Analogue Scale for Pain (VAS-P)	231
Palmieri	2002	Chronic low back pain: a study of the effects of manipulation under anesthesia	Numeric Rating Scale for Pain (NRS)	87
Paoloni	2011	Kinesio Taping applied to lumbar muscles influences clinical and electromyographic characteristics in chronic low back pain patients	Visual Analogue Scale for Pain (VAS-P)	39
Paolucci	2011	Psychological features and outcomes of the Back School treatment in patients with chronic non-specific low back pain.A randomized controlled study	SF-36	50
Peloso	2004	Analgesic efficacy and safety of tramadol/ acetaminophen combination tablets (Ultracet) in treatment of chronic low back pain: a multicenter, outpatient, randomized, double blind, placebo controlled trial	Visual Analogue Scale for Pain (VAS-P)	336
Penttinen	2002	Randomized controlled trial of back school with and without peer support	Oswestry Disability Index (ODI)	81
Perez-Palomares	2010	Percutaneous electrical nerve stimulation versus dry needling: Effectiveness in the treatment of chronic low back pain	Visual Analogue Scale for Pain (VAS-P)	124
Petersen	2002	The effect of McKenzie therapy as compared with that of intensive strengthening training for the treatment of patients with subacute or chronic low back pain: A randomized controlled trial	Low Back Pain Rating Scale (LBPRS) (Danish)	260
Pipino	1991	A direct myotonolytic (Pridinol Mesilate) for the management of chronic low back pain: A multicentre,comparative clinical evaluation.	Walking time on staircase	120
Pohjolainen	2000	Treatment of acute low back pain with the COX-2-selective anti-inflammatory drug nimesulide: results of a randomized, double-blind comparative trial versus ibuprofen.	Million Visual Analogue Scale (MVAS) (1 pain intensity, 2 stiffness in back , 3 pain relief)	96
Pope	1994	A prospective randomized three-week trial of spinal manipulation, transcutaneous muscle stimulation, massage and corset in the treatment of subacute low back pain	Visual Analogue Scale for Pain (VAS-P)	121
Powers	2008	Effects of a single session of posterior-to-anterior spinal mobilization and press-up exercise on pain response and lumbar spine extension in people with nonspecific low back pain	Visual Analogue Scale for Pain (VAS-P)	30
Preyde	2000	Effectiveness of massage therapy for subacute low-back pain: a randomized controlled trial	Roland-Morris Disability Questionnaire (RMDQ), McGill pain questionnaire	98
Puranik	2002	A randomised, single blind study to evaluate the effects of action potential simulator therapy compared with placebo in patients with chronic back pain	Visual Analogue Scale for Pain (VAS-P)	24
Pushpika	2010	Clinical evaluation of selected yogic procedures in individuals with low back pain	Grade of total effect (bespoke scale)	12
Rasmussen	2008	Manipulation does not add to the effect of extension exercises in chronic low-back pain (LBP). A randomized, controlled, double blind study	Visual Analogue Scale for Pain (VAS-P)	72
Rasmussen-Barr	2009	Graded exercise for recurrent low-back pain: a randomized, controlled trial with 6-, 12-, and 36-month follow-ups	Oswestry Disability Index (ODI), Visual analogue scale (VAS)	71
Rittweger	2002	Treatment of chronic lower back pain with lumbar extension and whole-body vibration exercise: a randomized controlled trial	Pain sensation and pain relief (VAS-P)	50
Ritvanen	2007	Dynamic surface electromyographic responses in chronic low back pain treated by traditional bone setting and conventional physical therapy	Oswestry Disability Index (ODI)	61
Roberts	2002	The back home trial: general practitioner-supported leaflets may change back pain behavior	Knowledge (questionnaire based on The Back Quiz, Jessep, 1991), attitude (six questions on managing back pain alone), observable behaviour (coded behaviour undertaking 4 activities) and function (Aberdeen Low Back Pain Scale)	58
Roche	2007	Comparison of a functional restoration program with active individual physical therapy for patients with chronic low back pain: a randomized controlled trial	Trunk flexibility: fingertip-to-floor (FTF)	132
Roche-Leboucher	2011	Multidisciplinary intensive functional restoration versus outpatient active physiotherapy in chronic low back pain: a randomized controlled trial	Reduction in the number of sick-leave days	131
Rose	1997	Chronic low back pain rehabilitation programs: a study of the optimum duration of treatment and a comparison of group and individual therapy.	Visual Analogue Scale for Pain (VAS-P)	104
Rossignol	2005	Measuring the contribution of pharmacological treatment to advice to stay active in patients with subacute low-back pain: a randomised controlled trial	Roland-Morris Disability Questionnaire (RMDQ)	318
Rozenberg	2002	Bed rest or normal activity for patients with acute low back pain: a randomized controlled trial	Visual Analogue Scale for Pain (VAS-P)	277
Ruoff	2003	Tramadol/acetaminophen combination tablets for the treatment of chronic lower back pain: a multicenter, randomized, double-blind, placebo-controlled outpatient study	Visual Analogue Scale for Pain (VAS-P)	318
Sahin	2011	Effectiveness of back school for treatment of pain and functional disability in patients with chronic low back pain: a randomized controlled trial	VAS-P (motion)	146
Salzmann	1992	Treatment of chronic low-back syndrome with tetrazepam in a placebo controlled double-blind trial.	VAS-P, return-to-normal Shober's index, improvement of spinal mobility by 50% in at least one special dimension	103
Sanders	1990	Chiropractic adjustive manipulation on subjects with acute low back pain: visual analog pain scores and plasma beta-endorphin levels.	States VAS (although may be NRS as term '5-point' is used)	18
Santaella	2009	Assessment of a biofeedback program to treat chronic low back pain	Visual Analogue Scale for Pain (VAS-P)	60
SatorKatzenschlager	2004	The short- and long-term benefit in chronic low back pain through adjuvant electrical versus manual auricular acupuncture	McGill pain questionnaire	61

Schattenkirchner	2003	A double-blind, multicentre, randomised clinical trial comparing the efficacy and tolerability of aceclofenac with diclofenac resinate in patients with acute low back pain	Visual Analogue Scale for Pain (VAS-P)	205
Schiltenwolf	2006	Comparison of a biopsychosocial therapy (BT) with a conventional biomedical therapy (MT) of subacute low back pain in the first episode of sick leave: a randomized controlled trial	Pain intensity (numeric rating scale (NRS))	64
Schnitzer	2000	Efficacy of tramadol in treatment of chronic low back pain	Time to exit from the double blind phase due to therapeutic failure (inadequate pain relief)	254
Senna	2011	Does maintained spinal manipulation therapy for chronic nonspecific low back pain result in better long-term outcome?	Oswestry Disability Index (ODI)	88
Shabat	2005	The effect of insoles on the incidence and severity of low back pain among workers whose job involves long-distance walking	Million Visual Analogue Scale (MVAS)	58
Shankar	2011	Autonomic status and pain profile in patients of chronic low back pain and following electro acupuncture therapy: a randomized control trial	Visual Analogue Scale for Pain (VAS-P)	60
Sherman	2005	Comparing yoga, exercise, and a self-care book for chronic low back pain: a randomized, controlled trial	Roland-Morris Disability Questionnaire (RMDQ), bothersomeness of back pain (11-point scale)	96
Sherman	2011	A randomized trial comparing yoga, stretching, and a self-care book for chronic low back pain	Roland Disability Questionnaire (RMDQ), NRS (11-point symptom bothersomeness)	206
Shirado	2010	Multicenter randomized controlled trial to evaluate the effect of home-based exercise on patients with chronic low back pain: the Japan low back pain exercise therapy study	Visual Analogue Scale for Pain (VAS-P)	175
Silva	2010	Evaluation of an extract of Brazilian arnica (Solidago chilensis Meyen, Asteraceae) in treating lumbago	Visual Analogue Scale for Pain (VAS-P)	20
Skljarevski	2009	A double-blind, randomized trial of duloxetine versus placebo in the management of chronic low back pain	Pain on a 0–10 weekly “mean average pain Likert scale” (coded as NRS as 11-point numeric rather than Likert)	404
Skljarevski	2010	Duloxetine versus placebo in patients with chronic low back pain: a 12-week, fixed-dose, randomized, double-blind trial	Brief Pain Inventory (BPI)	401
Skljarevski	2009	Maintenance of effect of duloxetine in patients with chronic low back pain	Brief Pain Inventory (BPI; modified short form)	177
Skljarevski	2010	Efficacy and safety of duloxetine 60 mg once-daily in patients with chronic low back pain	Brief Pain Inventory (BPI)	236
Sonne	1985	Injection of steroids and local anaesthetics as therapy for low-back pain.	Visual Analogue Scale for Pain (VAS-P)	29
Sorensen	2010	An educational approach based on a non-injury model compared with individual symptom-based physical training in chronic LBP. A pragmatic, randomised trial with a one-year follow-up	Numerical Rating Scale (NRS)	185
Soriano	1998	Gallium Arsenide Laser Treatment of chronic low back pain: a prospective randomised and double blind study.	Visual Analogue Scale for Pain (VAS-P)	71
Sokunbi	2007	Changes in plasma concentration of serotonin in response to spinal stabilisation exercises in chronic low back pain patient	Plasma levels of seratonin	22
Soukup	1999	The effect of a mensendieck exercise program as secondary prophylaxis for recurrent low back pain	Incidence of recurrent low back pain	69
Spinhoven	2004	Catastrophizing and internal pain control as mediators of outcome in the multidisciplinary treatment of chronic low back pain	Pain intensity (the pain rating index of the McGill pain questionnaire)	130
Staal	2004	Graded activity for low back pain in occupational health care: a randomized, controlled trial	Number of days absence from work over 6 months	134
Stankovic	1990	Conservative treatment of acute low-back pain. A prospective randomized trial: McKenzie method of treatment versus patient education in "mini back school"	Sick-leave suring the initial episode, sick leave during recurrences, recurrences of pain during the year of observation, patients' ability to self-help, pain over last 24 hrs (VAS- P), and spinal movements (4-point Likert)	95
Steenstra	2006	Economic evaluation of a multi-stage return to work program for workers on sick-leave due to low back pain	Return to work (RTW)	196
Storheim	2003	Intensive group training versus cognitive intervention in sub-acute low back pain: short-term results of a single-blind randomized controlled trial	Pain (VAS-P), disability (RMDQ), sick-listing (working days lost during 18 week study period), satisfaction with care (Deyo, 1998)	57
Strong	1998	Incorporating cognitive-behavioral therapy with occupational therapy: a comparative study with patients with low back pain	Integrated Psychosocial Assessment Model	30
Suen	2007	Auriculotherapy on low back pain in the elderly	Chinese Pain Intensity (verbal)	60
Suni	2006	Control of the lumbar neutral zone decreases low back pain and improves self-evaluated work ability: a 12-month randomized controlled study	VAS-P, Oswestry Disability Index (ODI), Pain and Disability index (PDI)	92
Sweetman	1987	Mefenamic acid, chlormezanone-paracetamol, ethoheptazine-aspirin-meprobamate: a comparative study in acute low back pain	Clinician's assessments, patient diary of pain severity	122
Szczurko	2007	Naturopathic care for chronic low back pain: a randomized trial	Oswestry Low Back Pain Disability(ODI), SF-36	59
Tao	2005	A randomized clinical trial of continuous low-level heat therapy for acute muscular low back pain in the workplace	Pain diary with pain intensity as NRS (0-10)	43
Tefner	2011	The effect of spa therapy in chronic low back pain: a randomized controlled, single-blind, follow-up study	Visual Analogue Scale for Pain (VAS-P)	37
Thomas	2005	Longer term clinical and economic benefits of offering acupuncture care to patients with chronic low back pain	SF-36 bodily pain dimension	239
Thomas	2006	Randomised controlled trial of a short course of traditional acupuncture compared with usual care for persistent non-specific low back pain	SF-36 bodily pain dimension	215
Toda	2002	Impact of waist/hip ratio on the therapeutic efficacy of lumbosacral corsets for chronic muscular low back pain	Quebec Back Pain Disability Scale (QBPDS)	143
Triano	1995	Manipulative therapy versus education programs in chronic low back pain	Perceived pain (VAS-P), functional activity (Oswestry (ODI), limited emotional status (modified zung depression index (ZDI),	129
Tritilanunt	2001	The efficacy of an aerobic exercise and health education program for treatment of chronic low back pain	Visual Analogue Scale for Pain (VAS-P)	68
Tsao	2007	Immediate changes in feedforward postural adjustments following voluntary motor training	EMG	22
Tuezuen	2003	Multicenter, randomized, double-blinded, placebo-controlled trial of thiocolchicoside in acute low back pain	Visual Analogue Scale for Pain (VAS-P)	137

Turner	1982	Comparison of group progressive-relaxation training and cognitive-behavioral group therapy for chronic low back pain	Visual Analogue Scale for Pain (VAS-P)	36
Turner	1988	Comparison of operant behavioral and cognitive-behavioral group treatment for chronic low back pain	McGill Pain Questionnaire (MPQ)	74
Turner	1990	Effectiveness of behavioral therapy for chronic low back pain: a component analysis	McGill Pain Questionnaire (MPQ)	76
Turner	1993	Efficacy of cognitive therapy for chronic low back pain	Visual Analogue Scale for Pain (VAS-P)	54
Underwood	2004	United Kingdom back pain exercise and manipulation (UK BEAM) randomised trial: Effectiveness of physical treatments for back pain in primary care	Roland Disability Questionnaire (RMDQ)	1029
Underwood	1998	The use of a back class teaching extension exercises in the treatment of acute low back pain in primary care	Oswestry Disability Index (ODI) , VAS-P	67
Unsgaard-Tondel	2010	Motor control exercises, sling exercises, and general exercises for patients with chronic low back pain: a randomized controlled trial with 1-year follow-up	Numeric Rating Scale for Pain (NRS)	109
Valle-Jones	1992	Controlled trial of a back support ('Lumbotrain') in patients with non-specific low back pain	Daily self-assessment (diary cards including VAS-P, limitation of activity VAS, ability to work or not, number of doses of analgesic taken during the day)	216
Van der Roer	2008	Intensive group training protocol versus guideline physiotherapy for patients with chronic low back pain: a randomised controlled trial	Roland Disability Questionnaire (RMDQ)	102
van Wijk	2005	Radiofrequency denervation of lumbar facet joints in the treatment of chronic low back pain: a randomized, double-blind, sham lesion-controlled trial	VAS-back pain, VAS-leg pain, physical activities (0-30 point scale), analgesic intake (0-8 points)	81
Vasseljen	2010	Abdominal muscle contraction thickness and function after specific and general exercises: A randomized controlled trial in chronic low back pain patients	Contraction thickness ratio in transversus abdominis, obliquus internus and externus	85
Vasseljen	2011	Effect of Core Stability Exercises on Feedforward Activation of Deep Abdominal Muscles in Chronic Low Back Pain: A Randomized Controlled Trial	Change in onset (before to after the intervention) of the deep abdominal muscles	87
Verbeek	2002	Early Occupational Health Management of Patientswith Back Pain	Time until return to work (measured at 1-year)	120
Videman	1984	Double-blind parallel study of meptazinol versus diflunisal in the treatment of lumbago	Oswestry Disability Index (ODI)	70
Vlaeyen	1995	Behavioural rehabilitation of chronic low back pain: comparison of an operant treatment, an operant-cognitive treatment and an operant-respondent treatment	Pain behaviour - Behavioural Approach Test (BAT)	56
VollenbroekHutten	2004	Differences in outcome of a multidisciplinary treatment between subgroups of chronic low back pain patients defined using two multiaxial assessment instruments: the multidimensional pain inventory and lumbar dynamometry	EQ-5D, RMDQ	152
Vong	2011	Motivational enhancement therapy in addition to physical therapy improves motivational factors and treatment outcomes in people with low back pain: a randomized controlled trial	Pain Rehabilitation Expectations Scale (PRES) and Pain Self-Efficacy Questionnaire (PSEQ)	76
VonKorff	2005	A trial of an activating intervention for chronic back pain in primary care and physical therapy settings	Roland Disability Questionnaire (RMDQ)	240
VonKorff	1998	A randomized trial of a lay person-led self-management group intervention for back pain patients in primary care	Roland Disability Questionnaire (RMDQ)	92
Waagen	1986	Short term trial of chiropractic adjustments for the relief of chronic low back pain	Visual Analogue Scale for Pain (VAS-P)	19
Wand	2004	Early intervention for the management of acute low back pain: a single-blind randomized controlled trial of biopsychosocial education, manual therapy, and exercise	Roland Disability Questionnaire (RMDQ)	65
Weiner	2008	Efficacy of percutaneous electrical nerve stimulation and therapeutic exercise for older adults with chronic low back pain: a randomized controlled trial	McGill Pain Questionnaire	184
Weiner	2003	Efficacy of Percutaneous Electrical Nerve Stimulation for the Treatment of Chronic Low Back Pain in Older Adults	McGill Pain Quest (MPQ) and Multi-dimensional Pain Inventory (MPI)	34
Wetherell	2011	A randomized, controlled trial of acceptance and commitment therapy and cognitive-behavioral therapy for chronic pain	Brief Pain Inventory (short form) Interference subscale (BPI)	99
White	2001	The effect of montage on the analgesic response to percutaneous neuromodulation therapy	Visual Analogue Scale for Pain (VAS-P)	72
Whitfill	2010	Early intervention options for acute low back pain patients: a randomized clinical trial with one-year follow-up outcomes	Obstacles to Return-to-Work (ORQ)	102
Wiesel	1980	Acute low-back pain. An objective analysis of conservative therapy	Numeric Rating Scale for Pain (NRS)	200
Williams	2009	Evaluation of the effectiveness and efficacy of Iyengar yoga therapy on chronic low back pain	Oswestry Disability Index (ODI)	90
Williams	2003	Randomized osteopathic manipulation study (ROMANS): Pragmatic trial for spinal pain in primary care	Extended Aberdeen Spine Pain Scale (EASPS)	186
Witt	2006	Pragmatic randomized trial evaluating the clinical and economic effectiveness of acupuncture for chronic low back pain	Hannover functional ability questionnaire (German)	2594
Worth S	2007	Real-time ultrasound feedback and abdominal hollowing exercises for people with low back pain	Retention test of abdominal holding examinination	19
Wright	2005	Individual active treatment combined with group exercise for acute and subacute low back pain	Rate of return-to-work	96
Wu	2004	Early intervention of aerobic exercise to rehabilitation of non-specific low back pain	VAS-P, Oswestry (ODI)	189
Yelland	2004	Prolotherapy Injections, Saline Injections, and Exercises for Chronic Low-Back Pain: A Randomized Trial	VAS-P, RMDQ	110
Yeung	2003	The use of electro-acupuncture in conjunction with exercise for the treatment of chronic low-back pain	NRS, Disability (Aberdeen LBP scale)	52
Yildirim	2010	Relationship between learning strategies of patients and proper perception of the home exercise program with non-specific low back pain	Assessing the Learning Strategies of Adults (ATLAS)	26
Yip	2004	The effectiveness of relaxation acupoint stimulation and acupressure with aromatic lavender essential oil for non-specific low back pain in Hong Kong: a randomised controlled trial	Visual Analogue Scale for Pain (VAS-P)	51
Yokoyama	2004	Comparison of Percutaneous Electrical Nerve Stimulationwith Transcutaneous Electrical Nerve Stimulation for Long-Term Pain Relief in Patients with Chronic Low Back Pain	Visual Analogue Scale for Pain (VAS-P)	53

Yozbatiran	2004	Effects of fitness and aquafitness exercises on physical fitness in patients with chronic low back pain	Musculoskeletal fitness	20
Zaringhalam	2010	Reduction of chronic non-specific low back pain: a randomised controlled clinical trial on acupuncture and baclofen	Visual Analogue Scale for Pain (VAS-P)	80
Zerbini	2005	Efficacy of etoricoxib 60 mg/day and diclofenac 150 mg/day in reduction of pain and disability in patients with chronic low back pain: results of a 4-week, multinational, randomized, double-blind study	Low back pain intensity scale (VAS)	401

[1–401]

References

1. Ackerman WE, Ahmad M: **Pain relief with intraarticular or medial branch nerve blocks in patients with positive lumbar facet joint SPECT imaging: A 12-week outcome study**. *Southern Medical Journal* 2008, **101**(9):931–934.
2. Agrifoglio E: **Aceclofenac: A new NSAID in the treatment of acute lumbago. Multicentre single blind study vs diclofenac**. *Acta theraputica* 1994, **20**.
3. Ahmed MS, Shakoor MA, Khan AA: **Evaluation of the effects of shortwave diathermy in patients with chronic low back pain**. *Bangladesh Med Res Counc Bull* 2009, **35**:18–20.
4. Akbari A, Khorashadizadeh S, Abdi G: **The effect of motor control exercise versus general exercise on lumbar local stabilizing muscles thickness: Randomized controlled trial of patients with chronic low back pain**. *Journal of Back and Musculoskeletal Rehabilitation* 2008, **21**(2):105–112.
5. Alaranta H, Rytokoski U, Rissanen A, Talo S, Ronnemaa T, Puukka P, Karppi SL, Videman T, Kallio V, Slatis P: **Intensive physical and psychosocial training program for patients with chronic low back pain. A controlled clinical trial**. *Spine* 1994, **19**(12):1339–49.
6. Albaladejo C, Kovacs FM, Royuela A, del Pino R, Zamora J: **The efficacy of a short education program and a short physiotherapy program for treating low back pain in primary care: a cluster randomized trial**. *Spine (Phila Pa 1976)* 2010, **35**(5):483–96.
7. Allan L, Richarz U, Simpson K, Slappendel R: **Transdermal fentanyl versus sustained release oral morphine in strong-opioid naive patients with chronic low back pain**. *Spine* 2005, **30**(22):2484–90.
8. Altmaier EM, Lehmann TR, Russell DW, Weinstein JN, Kao CF: **The effectiveness of psychological interventions for the rehabilitation of low back pain: a randomized controlled trial evaluation**. *Pain* 1992, **49**(3):329–35.
9. Amlie E, Weber H, Holme I: **Treatment of acute low-back pain with piroxicam: results of a double-blind placebo-controlled trial**. *Spine* 1987, **12**(5):473–6.
10. Andersson GB, Lucente T, Davis AM, Kappler RE, Lipton JA, Leurgans S: **A comparison of osteopathic spinal manipulation with standard care for patients with low back pain**. *N Engl J Med* 1999, **341**(19):1426–31.
11. Anema JR, Steenstra IA, Bongers PM, de Vet HC, Knol DL, Loisel P, van Mechelen W: **Multidisciplinary rehabilitation for subacute low back pain: graded activity or workplace intervention or both? A randomized controlled trial**. *Spine (Phila Pa 1976)* 2007, **32**(3):291–8; discussion 299–300.
12. Attanayake AMP, Somarathna K, Vyas GH, Dash SC: **Clinical evaluation of selected yogic procedures in individuals with low back pain**. *Ayu* 2010, **31**(2):245–50.
13. Aure OF, Nilsen JH, Vasseljen O: **Manual therapy and exercise therapy in patients with chronic low back pain: a randomized, controlled trial with 1-year follow-up**. *Spine* 2003, **28**(6):525–531.
14. Bakshi R: **Treatment of acute lumbosacral back pain with diclofenac resinate: results of a double-blind trial versus piroxicam.** *Drug investigation* 1994, **8**.
15. Balogh Z, Ordogh J, Gasz A, Nemet L, Bender T: **Effectiveness of balneotherapy in chronic low back pain – a randomized single-blind controlled follow-up study**. *Forsch Komplementarmed Klass Naturheilkd* 2005, **12**(4):196–201.
16. Bannwarth B, Allaert FA, Avouac B, Rossignol M, Rozenberg S, Valat JP: **A randomized, double-blind, placebo controlled triphosphate in study of oral adenosine subacute low back pain**. *The Journal of rheumatology.* 2005, **32**(6):1114–7.
17. Baratta R: **A double-blind study of cyclobenzaprine and placebo in the treatment of acute musculoskeletal conditions of the low back**. *curr theraputic research* 1982, **32**.
18. Basford JR, Sheffield CG, Harmsen WS: **Laser therapy: a randomized, controlled trial of the effects of low-intensity Nd:YAG laser irradiation on musculoskeletal back pain**. *Arch.Phys.Med Rehabil* 1999, **80**(6):647–652.

19. Basler HD, Jakle C, Kroner-Herwig B: **Incorporation of cognitive-behavioral treatment into the medical care of chronic low back patients: a controlled randomized study in German pain treatment centers**. *Patient Educ Couns* 1997, **31**(2):113–24.

20. Becker A, Leonhardt C, Kochen MM, Keller S, Wegscheider K, Baum E, Donner-Banzhoff N, Pfingsten M, Hildebrandt J, Basler HD, Chenot JF: **Effects of two guideline implementation strategies on patient outcomes in primary care: a cluster randomized controlled trial**. *Spine (Phila Pa 1976)* 2008, **33**(5):473–80.

21. Bekkering GE, Van TMW, Hendriks EJM, Koopmanschap MA, Knol DL, Bouter LM, Oostendorp RAB: **Implementation of clinical guidelines on physical therapy for patients with low back pain: Randomized trial comparing patient outcomes after a standard and active implementation strategy**. *Physical Therapy* 2005, **85**(6):544–555.

22. Bello AI, Kalu NH, Adegoke BOA, Agyepong-Badu S: **Hydrotherapy versus land-based exercises in the management of chronic low back pain: A comparative study**. *Journal of Musculoskeletal Research* 2010, **13**(4):159–165.

23. Ben Salah Frih Z, Fendri Y, Jellad A, Boudoukhane S, Rejeb N: **Efficacy and treatment compliance of a home-based rehabilitation programme for chronic low back pain: a randomized, controlled study**. *Ann Phys Rehabil Med* 2009, **52**(6):485–96.

24. Bendix AF, Bendix T, Lund C, Kirkbak S, Ostenfeld S: **Comparison of three intensive programs for chronic low back pain patients: a prospective, randomized, observer-blinded study with one-year follow-up**. *Scand J Rehabil Med* 1997, **29**(2):81–9.

25. Bendix AF, Bendix T, Ostenfeld S, Bush E, Andersen: **Active treatment programs for patients with chronic low back pain: a prospective, randomized, observer-blinded study**. *Eur.Spine J* 1995, **4**(3):148–152.

26. Bentsen H, Lindgarde F, Manthorpe R: **The effect of dynamic strength back exercise and/or a home training program in 57-year-old women with chronic low back pain. Results of a prospective randomized study with a 3-year follow-up period**. *Spine* 1997, **22**(13):1494–1500.

27. Bertalanffy A, Kober A, Bertalanffy P, Gustorff B, Gore O, Adel S, Hoerauf K: **Transcutaneous electrical nerve stimulation reduces acute low back pain during emergency transport**. *Academic emergency medicine : official journal of the Society for Academic Emergency Medicine.* 2005, **12**(7):607–11.

28. Berwick DM, Budman S, Feldstein M: **No clinical effect of back schools in an HMO. A randomized prospective trial**. *Spine* 1989, **14**(3):338–44.

29. Beurskens AJ, de Vet HC, Koke AJ, Lindeman E, Regtop W, van der Heijden GJ, Knipschild PG: **Efficacy of traction for non-specific low back pain: a randomised clinical trial**. *Lancet* 1995, **346**(8990):1596–1600.

30. Bialosky JE, Bishop MD, Robinson ME, Zeppieri J G, George SZ: **Spinal manipulative therapy has an immediate effect on thermal pain sensitivity in people with low back pain: a randomized controlled trial**. *Phys Ther* 2009, **89**(12):1292–303.

31. Bicalho E, Palma Setti JA, Macagnan J, Rivas Cano JL, Manffra EF: **Immediate effects of a high-velocity spine manipulation in paraspinal muscles activity of nonspecific chronic low-back pain subjects**. *Manual Therapy* 2010, **15**(5):469–475.

32. Birbara CA, Puopolo AD, Munoz DR, Sheldon EA, Mangione A, Bohidar NR, Geba GP: **Treatment of chronic low back pain with etoricoxib, a new cyclo-oxygenase-2 selective inhibitor: improvement in pain and disability–a randomized, placebo-controlled, 3-month trial**. *J Pain* 2003, **4**(6):307–15.

33. Birkenmaier C, Veihelmann A, Trouillier HH, Hausdorf J, von Schulze Pellengahr C: **Medial branch blocks versus pericapsular blocks in selecting patients for percutaneous cryodenervation of lumbar facet joints**. *Reg Anesth Pain Med* 2007, **32**:27–33.

34. Bishop PB, Quon JA, Fisher CG, Dvorak MF: **The Chiropractic Hospital-based Interventions Research Outcomes (CHIRO) study: a randomized controlled trial on the effectiveness of clinical practice guidelines in the medical and chiropractic management of patients with acute mechanical low back pain**. *Spine J* 2010, **10**(12):1055–64.

35. Blomberg S, Hallin G, Grann K, Berg E, Sennerby U: **Manual therapy with steroid injections–a new approach to treatment of low back pain. A controlled multicenter trial with an evaluation by orthopedic surgeons**. *Spine* 1994, **19**(5):569–77.

36. Blomberg S, Svardsudd K, Mildenberger F: **A controlled, multicentre trial of manual therapy in low-back pain. Initial status, sick-leave and pain score during follow-up**. *Scand J Prim Health Care* 1992, **10**(3):170–8.

37. Borman P, Keskin D, Bodur H: **The efficacy of lumbar traction in the management of patients with low back pain.** *Rheumatol Int* 2003, **23**(2):82–86, [http://dx.doi.org/10.1007/s00296-002-0249-0].

38. Brennan GP, Fritz JM, Hunter SJ, Thackeray A, Delitto A, Erhard RE: **Identifying subgroups of patients with acute/subacute "nonspecific" low back pain: results of a randomized clinical trial**. *Spine* 2006, **31**(6):623–31.

39. Brinkhaus B, Witt CM, Jena S, Linde K, Streng A, Wagenpfeil S, Irnich D, Walther HU, Melchart D, Willich SN: **Acupuncture in patients with chronic low back pain: a randomized controlled trial**. *Archives of internal medicine.* 2006, **166**(4):450–7.

40. Bronfort G, Goldsmith CH, Nelson CF, Boline PD, Anderson AV: **Trunk exercise combined with spinal manipulative or NSAID therapy for chronic low back pain: a randomized, observer-blinded clinical trial**. *J Manipulative Physiol Ther.* 1996, **19**(9):570–582.

41. Brown J F L, Bodison S, Dixon J, Davis W, Nowoslawski J: **Comparison of diflunisal and acetaminophen with codeine in the treatment of initial or recurrent acute low back strain**. *Clin Ther* 1986, **9 Suppl C**:52–8.

42. Bruce B, Lorig K, Laurent D, Ritter P: **The impact of a moderated e-mail discussion group on use of complementary and alternative therapies in subjects with recurrent back pain**. *Patient Education and Counseling* 2005, **58**(3):305–311.

43. Bruehl S, Chung OY, Burns JW, Diedrich L: **Trait anger expressiveness and pain-induced beta-endorphin release: support for the opioid dysfunction hypothesis**. *Pain* 2007, **130**(3):208–15.

44. Bruehl S, Burns JW, Chung OY, Quartana P: **Anger management style and emotional reactivity to noxious stimuli among chronic pain patients and healthy controls: the role of endogenous opioids**. *Health Psychol* 2008, **27**(2):204–14.

45. Buerger AA: **A controlled trial of rotational manipulation in low back pain.** *Manuelle Medizine* 1980, **2**.

46. Burton K: **Information and Advice to Patients With Back Pain Can Have a Positive Effect**. *Spine* 1999, **24**(23).

47. Bush C, Ditto B, Feuerstein M: **A controlled evaluation of paraspinal EMG biofeedback in the treatment of chronic low back pain**. *Health Psychol* 1985, **4**(4):307–21.

48. Cairns MC, Foster NE, Wright C: **Randomized controlled trial of specific spinal stabilization exercises and conventional physiotherapy for recurrent low back pain**. *Spine* 2006, **31**(19):E670–81.

49. Cairns MC, Foster NE, Wright CC, Pennington D: **Level of distress in a recurrent low back pain population referred for physical therapy**. *Spine* 2003, **28**(9):953–9.

50. Callaghan M: **Evaluation of a back rehabilitation group of chronic back pain in an outpatient setting**. *Physiotherapy* 1994, **10**:677–681.

51. Calmels P, Queneau P, Hamonet C, Le Pen C, Maurel F, Lerouvreur C, Thoumie P: **Effectiveness of a lumbar belt in subacute low back pain: an open, multicentric, and randomized clinical study**. *Spine (Phila Pa 1976)* 2009, **34**(3):215–20.

52. Carr JL, Klaber MJA, Howarth E, Richmond SJ, Torgerson DJ, Jackson DA, Metcalfe CJ: **A randomized trial comparing a group exercise programme for back pain patients with individual physiotherapy in a severely deprived area**. *Disability and Rehabilitation* 2005, **27**(16):929–937.

53. Cecchi F, Molino-Lova R, Chiti M, Pasquini G, Paperini A, Conti AA, Macchi C: **Spinal manipulation compared with back school and with individually delivered physiotherapy for the treatment of chronic low back pain: a randomized trial with one-year follow-up**. *Clin Rehabil* 2010, **24**:26–36.

54. Chan CW, Mok NW, Yeung EW: **Aerobic exercise training in addition to conventional physiotherapy for chronic low back pain: a randomized controlled trial**. *Arch Phys Med Rehabil* 2011, **92**(10):1681–5.

55. Chang ST, Chen LC, Chang CC, Chu HY, Tsai KC: **Effects of piroxicam-beta-cyclodextrin sachets on abnormal postural sway in patients with chronic low back pain**. *J Clin Pharm Ther* 2008, **33**(5):495–506.

56. Chatchawan U, Thinkhamrop B, Kharmwan S, Knowles J, Eungpinichpong W: **Effectiveness of traditional Thai massage versus Swedish massage among patients with back pain associated with myofascial trigger points**. *Journal of Bodywork & Movement Therapies* 2005, **9**(4):298–309.

57. Chatzitheodorou D, Mavromoustakos S, Milioti S: **The effect of exercise on adrenocortical responsiveness of patients with chronic low back pain, controlled for psychological strain**. *Clin Rehabil* 2008, **22**(4):319–28.

58. Cheing G: **Transcutaneous Electrical Nerve Stimulation: Nonparallel Antinociceptive Effects on Chronic Clinical Pain and Acute Experimental Pain**. *Arch Phys Med Rehabil* 1999, **80**.

59. Cherkin DC: **Pitfalls of Patient Education: Limited Success of a Program for Back Pain in Primary Care**. *Spine* 1996, **21**(3).

60. Cherkin DC, Deyo RA, Battie M, Street J, Barlow W: **A Comparison of Physical Therapy, Chiropractic Manipulation, and Provision of an Educational Booklet for the Treatment of Patients with Low Back Pain**. *The New England Journal of Medicine* 1998, **339**(15):1021–1029.

61. Cherkin DC, Eisenberg D, Sherman KJ, Barlow W, Kaptchuk TJ, Street J, Deyo RA: **Randomized trial comparing traditional Chinese medical acupuncture, therapeutic massage, and self-care education for chronic low back pain**. *Arch Intern Med* 2001, **161**(8):1081–8.

62. Cherkin DC, Sherman KJ, Avins AL, Erro JH, Ichikawa L, Barlow WE, Delaney K, Hawkes R, Hamilton L, Pressman A, Khalsa PS, Deyo RA: **A randomized trial comparing acupuncture, simulated acupuncture, and usual care for chronic low back pain**. *Arch Intern Med* 2009, **169**(9):858–66.

63. Cherkin DC, Sherman KJ, Kahn J, Wellman R, Cook AJ, Johnson E, Erro J, Delaney K, Deyo RA: **A comparison of the effects of 2 types of massage and usual care on chronic low back pain: a randomized, controlled trial**. *Ann Intern Med* 2011, **155**:1–9.

64. Childs JD, Fritz JM, Flynn TW, Irrgang JJ, Johnson KK, Majkowski GR, Delitto A: **A clinical prediction rule to identify patients with low back pain most likely to benefit from spinal manipulation: a validation study**. *Ann Intern Med* 2004, **141**(12):920–8.

65. Chiradejnant A, Maher CG, Latimer J, Stepkovitch N: **Efficacy of "therapist-selected" versus "randomly selected" mobilisation techniques for the treatment of low back pain: a randomised controlled trial**. *Aust J Physiother* 2003, **49**(4):233–41.

66. Chiu CK, Low TH, Tey YS, Singh VA, Shong HK: **The efficacy and safety of intramuscular injections of methylcobalamin in patients with chronic nonspecific low back pain: a randomised controlled trial**. *Singapore Med J* 2011, **52**(12):868–73.

67. Chok B, Lee R, Latimer J, Tan SB: **Endurance training of the trunk extensor muscles in people with subacute low back pain**. *Phys Ther* 1999, **79**(11):1032–42.

68. Chown M, Whittamore L, Rush M, Allan S, Stott D, Archer M: **A prospective study of patients with chronic back pain randomised to group exercise, physiotherapy or osteopathy**. *Physiotherapy* 2008, **94**:21–28.

69. Chrubasik s: **Treatment of low back pain with a herbal or synthetic anti-rheumatic**. *Rheumatology.(Oxford)* 2001, **40**.

70. Cleland JA, Fritz JM, Kulig K, Davenport TE, Eberhart S, Magel J, Childs JD: **Comparison of the effectiveness of three manual physical therapy techniques in a subgroup of patients with low back pain who satisfy a clinical prediction rule: a randomized clinical trial**. *Spine (Phila Pa 1976)* 2009, **34**(25):2720–9.

71. Coats TL, Borenstein DG, Nangia NK, Brown MT: **Effects of valdecoxib in the treatment of chronic low back pain: results of a randomized, placebo-controlled trial**. *Clinical therapeutics.* 2004, **26**(8):1249–60.

72. Costa LO, Maher CG, Latimer J, Hodges PW, Herbert RD, Refshauge KM, McAuley JH, Jennings MD: **Motor control exercise for chronic low back pain: a randomized placebo-controlled trial**. *Phys Ther* 2009, **89**(12):1275–86.

73. Cramer GD, Humphreys CR, Hondras MA, McGregor M, Triano JJ: **The Hmax/Mmax ratio as an outcome measure for acute low back pain.** *J Manipulative Physiol Ther* 1993, **16**:7–13.

74. Cuesta-Vargas AI, Garcia-Romero JC, Arroyo-Morales M, Diego-Acosta AM, Daly DJ: **Exercise, manual therapy, and education with or without high-intensity deep-water running for nonspecific chronic low back pain: a pragmatic randomized controlled trial**. *Am J Phys Med Rehabil* 2011, **90**(7):526–34; quiz 535–8.

75. Damush TM, Weinberger M, Perkins SM, Rao JK, Tierney WM, Qi R, Clark DO: **The long-term effects of a self-management program for inner-city primary care patients with acute low back pain**. *Archives of internal medicine.* 2003, **163**(21):2632–8.

76. Dapas F, Hartman SF, Martinez L, Northrup BE, Nussdorf RT, Silberman HM, Gross H: **Baclofen for the treatment of acute low-back syndrome. A double-blind comparison with placebo**. *Spine* 1985, **10**(4):345–9.

77. Dechow E, Davies RK, Carr AJ, Thompson PW: **A randomized, double-blind, placebo-controlled trial of sclerosing injections in patients with chronic low back pain**. *Rheumatology (Oxford)* 1999, **38**(12):1255–9.

78. Defrin R, Ben Benyamin S, Aldubi RD, Pick CG: **Conservative correction of leg-length discrepancies of 10mm or less for the relief of chronic low back pain**. *Arch Phys Med Rehabil* 2005, **86**(11):2075–80.

79. Demirel R, Ucok K, Kavuncu V, Gecici O, Evcik D, Dundar U, Solak O, Mollaoglu H: **Effects of balneotherapy with exercise in patients with low back pain**. *Journal of Back and Musculoskeletal Rehabilitation* 2008, **21**(4):263–272.

80. Demoulin C, Grosdent S, Capron L, Tomasella M, Somville PR, Crielaard JM, Vanderthommen M: **Effectiveness of a semi-intensive multidisciplinary outpatient rehabilitation program in chronic low back pain**. *Joint Bone Spine* 2010, **77**:58–63.

81. Descarreaux M, Normand MC, Laurencelle L, Dugas C: **Evaluation of a specific home exercise program for low back pain.** *J Manipulative Physiol Ther* 2002, **25**(8):497–503.

82. Dettori JR, Bullock SH, Sutlive TG, Franklin RJ, Patience T: **The effects of spinal flexion and extension exercises and their associated postures in patients with acute low back pain**. *Spine* 1995, **20**(21):2303–12.

83. Deyo RA, Diehl AK, Rosenthal M: **How many days of bed rest for acute low back pain? A randomized clinical trial**. *N Engl J Med* 1986, **315**(17):1064–70.

84. Deyo RA, Walsh NE, Martin DC, Schoenfeld LS, Ramamurthy S: **A controlled trial of transcutaneous electrical nerve stimulation (TENS) and exercise for chronic low back pain**. *N.Engl.J.Med.* 1990, **322**(23):1627–1634.

85. Di Cesare A, Giombini A, Di Cesare M, Ripani M, Vulpiani MC, Saraceni VM: **Comparison between the effects of trigger point mesotherapy versus acupuncture points mesotherapy in the treatment of chronic low back pain: a short term randomized controlled trial**. *Complement Ther Med* 2011, **19**:19–26.

86. Diaz Arribas MJ, Ramos Sanchez M, Pardo Hervas P, Lopez Chicharro J, Angulo Carrere T, Ortega Molina P, Astasio Arbiza P: **Effectiveness of the physical therapy Godelive Denys-Struyf method for nonspecific low back pain: primary care randomized control trial**. *Spine (Phila Pa 1976)* 2009, **34**(15):1529–38.

87. Djais N, Kalim H: **The role of lumbar spine radiography in the outcomes of patients with simple acute low back pain**. *APLAR Journal of Rheumatology* 2005, **8**:45–50.

88. Djavid GE, Mehrdad R, Ghasemi M, Hasan-Zadeh H, Sotoodeh-Manesh A, Pouryaghoub G: **In chronic low back pain, low level laser therapy combined with exercise is more beneficial than exercise alone in the long term: a randomised trial**. *Aust J Physiother* 2007, **53**(3):155–60.

89. Donchin M, Woolf O, Kaplan L, Floman Y: **Secondary prevention of low-back pain. A clinical trial**. *Spine* 1990, **15**(12):1317–20.

90. Donzelli S, Domenica E, Cova AM, Galletti R, Giunta N: **Two different techniques in the rehabilitation treatment of low back pain: a randomized controlled trial**. *Eura Medicophys.* 2006, **42**(3):205–10.

91. Dreiser RL, Marty M, Ionescu E, Gold M, Liu JH: **Relief of acute low back pain with diclofenac-K 12.5 mg tablets: a flexible dose, ibuprofen 200 mg and placebo-controlled clinical trial**. *International journal of clinical pharmacology and therapeutics.* 2003, **41**(9):375–85.

92. Dufour N, Thamsborg G, Oefeldt A, Lundsgaard C, Stender S: **Treatment of chronic low back pain: a randomized, clinical trial comparing group-based multidisciplinary biopsychosocial rehabilitation and intensive individual therapist-assisted back muscle strengthening exercises**. *Spine (Phila Pa 1976)* 2010, **35**(5):469–76.

93. Dundar U, Solak O, Yigit I, Evcik D, Kavuncu V: **Clinical effectiveness of aquatic exercise to treat chronic low back pain: a randomized controlled trial**. *Spine (Phila Pa 1976)* 2009, **34**(14):1436–40.

94. Durmus D, Akyol Y, Alayli G, Tander B, Zahiroglu Y, Canturk F: **Effects of electrical stimulation program on trunk muscle strength, functional capacity, quality of life, and depression in the patients with low back pain: a randomized controlled trial**. *Rheumatol Int* 2009, **29**(8):947–54.

95. Durmus D, Akyol Y, Cengiz K, Terzi T, Canturk F: **Effects of therapeutic ultrasound on pain, disability, walking performance, quality of life, and depression in patients with chronic low back pain: A randomized, placebo controlled trial**. *Turkish Journal of Rheumatology* 2010, **25**(2):82–87.

96. Durmus D, Durmaz Y, Canturk F: **Effects of therapeutic ultrasound and electrical stimulation program on pain, trunk muscle strength, disability, walking performance, quality of life, and depression in patients with low back pain: a randomized-controlled trial**. *Rheumatol Int* 2010, **30**(7):901–10.

97. Elnaggar IM, Nordin M, Sheikhzadeh A, Parnianpour M, Kahanovitz N: **Effects of spinal flexion and extension exercises on low-back pain and spinal mobility in chronic mechanical low-back pain patients**. *Spine* 1991, **16**(8):967–972.

98. Engbert K, Weber M: **The effects of therapeutic climbing in patients with chronic low back pain: a randomized controlled study**. *Spine (Phila Pa 1976)* 2011, **36**(11):842–9.

99. Ergun H, Polat O, Demirkan NA, Gunalp M, Gurler S: **The efficacy, safety, and pharmacokinetics of intramuscular and oral phenyramidol in patients with low back pain in an emergency department**. *Turkish Journal of Medical Sciences* 2010, **40**:71–76.

100. Estlander AM, Mellin G, Vanharanta H, Hupli M: **Effects and follow-up of a multimodal treatment program including intensive physical training for low back pain patients**. *Scand J Rehabil Med* 1991, **23**(2):97–102.

101. Ewert T, Limm H, Wessels T, Rackwitz B, von Garnier K, Freumuth R, Stucki G: **The comparative effectiveness of a multimodal program versus exercise alone for the secondary prevention of chronic low back pain and disability**. *PM R* 2009, **1**(9):798–808.

102. Fairbank J, Frost H, Wilson MacDonald J, Yu LM, Barker K, Collins R, Spine Stabilisation Trial G: **Randomised controlled trial to compare surgical stabilisation of the lumbar spine with an intensive rehabilitation programme for patients with chronic low back pain: the MRC spine stabilisation trial**. *BMJ (Clinical research ed.)* 2005, **330**(7502):1233.

103. Farhadi K, Schwebel DC, Saeb M, Choubsaz M, Mohammadi R, Ahmadi A: **The effectiveness of wet-cupping for nonspecific low back pain in Iran: a randomized controlled trial**. *Complement Ther Med* 2009, **17**:9–15.

104. Ferreira ML, Ferreira PH, Latimer J, Herbert RD, Hodges PW, Jennings MD, Maher CG, Refshauge KM: **Comparison of general exercise, motor control exercise and spinal manipulative therapy for chronic low back pain: A randomized trial**. *Pain* 2007, **131**(1-2):31–7.

105. Ferreira ML, Ferreira PH, Latimer J, Herbert RD, Maher C, Refshauge K: **Relationship between spinal stiffness and outcome in patients with chronic low back pain**. *Man Ther* 2009, **14**:61–7.

106. Field T, Hernandez-Reif M, Diego M, Fraser M: **Lower back pain and sleep disturbance are reduced following massage therapy**. *Journal of Bodywork and Movement Therapies* 2007, **11**(2):141–145.

107. Fiore P, Panza F, Cassatella G, Russo A, Frisardi V, Solfrizzi V, Ranieri M, Di Teo L, Santamato A: **Short-term effects of high-intensity laser therapy versus ultrasound therapy in the treatment of low back pain: a randomized controlled trial**. *Eur J Phys Rehabil Med* 2011, **47**(3):367–73.

108. da Fonseca JL, Magini M, de Freitas TH: **Laboratory gait analysis in patients with low back pain before and after a pilates intervention**. *J Sport Rehabil* 2009, **18**(2):269–82.

109. Franca FR, Burke TN, Hanada ES, Marques AP: **Segmental stabilization and muscular strengthening in chronic low back pain: a comparative study**. *Clinics (Sao Paulo)* 2010, **65**(10):1013–7.

110. Frerick H, Keitel W, Kuhn U, Schmidt S, Bredehorst A, Kuhlmann M: **Topical treatment of chronic low back pain with a capsicum plaster**. *Pain* 2003, **106**(1-2):59–64.

111. Friedrich M, Gittler G: **Long-term effects of a combined exercise and motivation program in patients with chronic low back pain: A five-year follow-up**. *Pain Practice* 2009, **9**:121.

112. Friedrich M, Gittler G, Arendasy M, Friedrich KM: **Long-term effect of a combined exercise and motivational program on the level of disability of patients with chronic low back pain**. *Spine.* 2005, **30**(9):995–1000.

113. Fritzell P: **Lumbar fusion versus non-surgical treatment for chronic low back pain. A multicenter randomized controlled trial from the swedish lumbar spine study**. *Spine* 2001, **26**:2521–2534.

114. H F, SE L, HA D, PT C, S SB: **Randomised controlled trial of physiotherapy compared with advice for low back pain.** *BMJ* 2004, **329**(7468):708.

115. Frost H, Klaber Moffett JA, Moser JS, Fairbank JC: **Randomised controlled trial for evaluation of fitness programme for patients with chronic low back pain**. *BMJ* 1995, **310**(6973):151–154.

116. Fuchs S, Erbe T, Fischer HL, Tibesku CO: **Intraarticular hyaluronic acid versus glucocorticoid injections for nonradicular pain in the lumbar spine**. *Journal of vascular and interventional radiology : JVIR.* 2005, **16**(11):1493–8.

117. Garvey TA, Marks MR, Wiesel SW: **A prospective, randomized, double-blind evaluation of trigger-point injection therapy for low-back pain**. *Spine* 1989, **14**(9):962–4.

118. Gatchel RJ, Polatin PB, Noe C, Gardea M, Pulliam C, Thompson J: **Treatment- and cost-effectiveness of early intervention for acute low-back pain patients: a one-year prospective study**. *Journal of occupational rehabilitation.* 2003, **13**:1–9.

119. Geisser ME, Wiggert EA, Haig AJ, Colwell MO: **A randomized, controlled trial of manual therapy and specific adjuvant exercise for chronic low back pain**. *Clinical Journal of Pain* 2005, **21**(6):463–470.

120. George SZ, Fritz JM, Bialosky JE, Donald DA: **The effect of a fear-avoidance-based physical therapy intervention for patients with acute low back pain: results of a randomized clinical trial**. *Spine.* 2003, **28**(23):2551–60.

121. George SZ, Fritz JM, Childs JD, Brennan GP: **Sex differences in predictors of outcome in selected physical therapy interventions for acute low back pain.** *J Orthop Sports Phys Ther* 2006, **36**(6):354–363, [http://dx.doi.org/10.2519/jospt.2006.2270].

122. George SZ, Hirsh AT: **Distinguishing patient satisfaction with treatment delivery from treatment effect: a preliminary investigation of patient satisfaction with symptoms after physical therapy treatment of low back pain**. *Archives of physical medicine and rehabilitation.* 2005, **86**(7):1338–44.

123. Gibson T, Grahame R, Harkness J, Woo P, Blagrave P, Hills R: **Controlled comparison of short-wave diathermy treatment with osteopathic treatment in non-specific low back pain**. *Lancet* 1985, **1**(8440):1258–61.

124. Gilbert: **Low Back Pain: Influence of Early MR Imaging or CT on Treatment and Outcome? Multicenter Randomized Trial**. *radiology* 2004, (231):343.

125. Gladwell V, Head S, Haggar M, Beneke R: **Does a program of Pilates improve chronic non-specific low back pain?** *Journal of Sports Rehabilitation* 2006, **15**(4):338–350.

126. Glazov G, Schattner P, Lopez D, Shandley K: **Laser acupuncture for chronic non-specific low back pain: a controlled clinical trial**. *Acupunct Med* 2009, **27**(3):94–100.

127. Ghner W, Schlicht W: **Preventing chronic back pain: evaluation of a theory-based cognitive-behavioural training programme for patients with subacute back pain**. *Patient Educ Couns.* 2006, **64**(1-3):87–95.

128. Goldby LJ, Moore AP, Doust J, Trew ME: **A randomized controlled trial investigating the efficiency of musculoskeletal physiotherapy on chronic low back disorder**. *Spine* 2006, **31**(10):1083–93.

129. Goldstein MS, Morgenstern H, Hurwitz EL, Yu F: **The impact of treatment confidence on pain and related disability among patients with low-back pain: results from the University of California, Los Angeles, low-back pain study**. *Spine J* 2002, **2**(6):391–9; discussion 399–401.

130. Grant DJ, Bishop-Miller J, Winchester DM, Anderson M, Faulkner S: **A randomized comparative trial of acupuncture versus transcutaneous electrical nerve stimulation for chronic back pain in the elderly**. *Pain* 1999, **82**:9–13.

131. Gudavalli MR, Cambron JA, McGregor M, Jedlicka J, Keenum M, Ghanayem AJ, Patwardhan AG: **A randomized clinical trial and subgroup analysis to compare flexion-distraction with active exercise for chronic low back pain**. *Eur Spine J* 2006, **15**(7):1070–82.

132. Guthrie RJ, Grindstaff TL, Croy T, Ingersoll CD, Saliba SA: **The Effect of Traditional Bridging or Suspension Exercise Bridging on Lateral Abdominal Thickness in Individuals with Low Back Pain**. *J Sport Rehabil* 2011.

133. Gunn CC, Milbrandt WE, Little AS, Mason KE: **Dry needling of muscle motor points for chronic low-back pain: a randomized clinical trial with long-term follow-up**. *Spine* 1980, **5**(3):279–91.

134. Gur A, Karakoc M, Cevik R, Nas K, Sarac AJ, Karakoc M: **Efficacy of low power laser therapy and exercise on pain and functions in chronic low back pain**. *Lasers Surg.Med* 2003, **32**(3):233–238.

135. Haake M, Muller HH, Schade-Brittinger C, Basler HD, Schafer H, Maier C, Endres HG, Trampisch HJ, Molsberger A: **German Acupuncture Trials (GERAC) for chronic low back pain: randomized, multicenter, blinded, parallel-group trial with 3 groups**. *Arch Intern Med* 2007, **167**(17):1892–8.

136. Haas M, Groupp E, Muench J, Kraemer D, Brummel Smith K, Sharma R, Ganger B, Attwood M, Fairweather A: **Chronic disease self-management program for low back pain in the elderly**. *Journal of Manipulative & Physiological Therapeutics* 2005, **28**(4):228–37.

137. Hackett GI, Seddon D, Kaminski D: **Electroacupuncture compared with paracetamol for acute low back pain**. *Practitioner* 1988, **232**(1443):163–4.

138. Hadler NM, Curtis P, Gillings DB, Stinnett S: **A benefit of spinal manipulation as adjunctive therapy for acute low-back pain: a stratified controlled trial**. *Spine* 1987, **12**(7):702–6.

139. Hagen EM, Odelien KH, Lie SA, Eriksen HR: **Adding a physical exercise programme to brief intervention for low back pain patients did not increase return to work**. *Scand J Public Health* 2010, **38**(7):731–8.

140. Hall AM, Maher CG, Lam P, Ferreira M, Latimer J: **Tai chi exercise for treatment of pain and disability in people with persistent low back pain: a randomized controlled trial**. *Arthritis Care Res (Hoboken)* 2011, **63**(11):1576–83.

141. Hancock MJ, Maher CG, Latimer J, McLachlan AJ, Cooper CW, Day RO, Spindler MF, McAuley JH: **Assessment of diclofenac or spinal manipulative therapy, or both, in addition to recommended first-line treatment for acute low back pain: a randomised controlled trial**. *Lancet* 2007, **370**(9599):1638–43.

142. Hansen FR, Bendix T, Skov P, Jensen CV, Kristensen JH, Krohn L, Schioeler H: **Intensive, dynamic back-muscle exercises, conventional physiotherapy, or placebo-control treatment of low-back pain. A randomized, observer-blind trial**. *Spine* 1993, **18**:98–108.

143. Harkapaa K, Jarvikoski A, Mellin G, Hurri H: **A controlled study on the outcome of inpatient and outpatient treatment of low back pain. Part I. Pain, disability, compliance, and reported treatment benefits three months after treatment**. *Scand J Rehabil Med* 1989, **21**(2):81–9.

144. Harts CC, Helmhout PH, de Bie RA, Staal JB: **A high-intensity lumbar extensor strengthening program is little better than a low-intensity program or a waiting list control group for chronic low back pain: a randomised clinical trial**. *Aust J Physiother* 2008, **54**:23–31.

145. Hartvigsen J, Lauritzen S, Lings S, Lauritzen T: **Intensive education combined with low tech ergonomic intervention does not prevent low back pain in nurses**. *Occup.Environ.Med.* 2005, **62**:13–17.

146. Hartvigsen J, Morso L, Bendix T, Manniche C: **Supervised and non-supervised Nordic walking in the treatment of chronic low back pain: a single blind randomized clinical trial**. *BMC Musculoskelet Disord* 2010, **11**:30.

147. Hasson D, Arnetz B, Jelveus L, Edelstam B: **A Randomized Clinical Trial of the Treatment Effects of Massage Compared to Relaxation Tape Recordings on Diffuse Long-Term Pain**. *Psychotherapy and Psychosomatics* 2004, **73**:17–24.

148. Hawk C, Long CR, Rowell RM, Gudavalli MR, Jedlicka J: **A randomized trial investigating a chiropractic manual placebo: a novel design using standardized forces in the delivery of active and control treatments**. *J Altern Complement Med* 2005, **11**:109–17.

149. Hay EM, Mullis R, Lewis M, Vohora K, Main CJ, Watson P, Dziedzic KS, Sim J, Lowe CM, Croft PR: **Comparison of physical treatments versus a brief pain-management programme for back pain in primary care: a randomised clinical trial in physiotherapy practice**. *Lancet.* 2005, **365**(9476):2024–30.

150. Hazard R: **A Controlled Trial of an Educational Pamphlet to Prevent Disability After Occupational Low Back Injury**. *Spine* 2000, **25**(11).

151. Helliwell S P: **Manipulation in low-back pain**. *The Physician* 1987, :187–8.

152. Hemmila HM, Keinanen-Kiukaanniemi SM, Levoska S, Puska P: **Long-term effectiveness of bone-setting, light exercise therapy, and physiotherapy for prolonged back pain: a randomized controlled trial**. *J Manipulative Physiol Ther.* 2002, **25**(2):99–104.

153. Henchoz Y, de Goumoens P, Norberg M, Paillex R, So AK: **Role of physical exercise in low back pain rehabilitation: a randomized controlled trial of a three-month exercise program in patients who have completed multidisciplinary rehabilitation**. *Spine (Phila Pa 1976)* 2010, **35**(12):1192–9.

154. Henchoz Y, de Goumoens P, So AK, Paillex R: **Functional multidisciplinary rehabilitation versus outpatient physiotherapy for non specific low back pain: randomized controlled trial**. *Swiss Med Wkly* 2010, **140**:w13133.

155. Herman E, Williams R, Stratford P, Fargas-Babjak A, Trott M: **A randomized controlled trial of transcutaneous electrical nerve stimulation (CODETRON) to determine its benefits in a rehabilitation program for acute occupational low back pain**. *Spine* 1994, **19**(5):561–568.

156. Hernandez-Reif M, Field T, Krasnegor J, Theakston H: **Lower back pain is reduced and range of motion increased after massage therapy.** *Int J Neurosci* 2001, **106**(3-4):131–145.

157. Hertzman Miller RP, Morgenstern H, Hurwitz EL, Yu F, Adams AH, Harber P, Kominski GF: **Comparing the satisfaction of low back pain patients randomized to receive medical or chiropractic care: results from the UCLA low-back pain study**. *American journal of public health.* 2002, **92**(10):1628–33.

158. Heymans MW, Vet HC, Bongers PM, Knol DL, Koes BW, Mechelen W: **The effectiveness of high-intensity versus low-intensity back schools in an occupational setting: a pragmatic randomized controlled trial**. *Spine* 2006, **31**(10):1075–82.

159. Hickey RF: **Chronic low back pain: a comparison of diflunisal with paracetamol**. *N Z Med J* 1982, **95**(707):312–4.

160. Hoehler FK, Tobis JS, Buerger AA: **Spinal manipulation for low back pain**. *Jama* 1981, **245**(18):1835–8.

161. Hoiriis KT, Pfleger B, McDuffie FC, Cotsonis G, Elsangak O, Hinson R, Verzosa GT: **A randomized clinical trial comparing chiropractic adjustments to muscle relaxants for subacute low back pain**. *Journal of manipulative and physiological therapeutics.* 2004, **27**(6):388–98.

162. Hondras MA, Long CR, Cao Y, Rowell RM, Meeker WC: **A randomized controlled trial comparing 2 types of spinal manipulation and minimal conservative medical care for adults 55 years and older with subacute or chronic low back pain**. *J Manipulative Physiol Ther* 2009, **32**(5):330–43.

163. Hosie G: **The topical NSAID, felbinac, versus oral ibuprofen: a comparison of efficacy in the treatment of acute lower back injury**. *Br j clin res* 1993, **4**:5–17.

164. Hsieh CYJ, Adams AH, Tobis J, Hong CZ, Danielson C, Platt K, Hoehler F, Reinsch S, Rubel A: **Effectiveness of four conservative treatments for subacute low back pain: a randomized clinical trial.** *Spine (Phila Pa 1976)* 2002, **27**(11):1142–1148.

165. Hsieh LL, Kuo CH, Lee LH, Yen AM, Chien KL, Chen TH: **Treatment of low back pain by acupressure and physical therapy: randomised controlled trial**. *BMJ (Clinical research ed.)* 2006, **332**(7543):696–700.

166. Hsieh LL, Kuo CH, Yen MF, Chen TH: **A randomized controlled clinical trial for low back pain treated by acupressure and physical therapy**. *Prev Med* 2004, **39**:168–76.

167. Hsieh R: **One-Shot Percutaneous Electrical Nerve Stimulation vs. Transcutaneous Electrical Nerve Stimulation for Low Back Pain**. *American journal of physical medicine and rehabilitation* 2002, **81**(11).

168. Hurley DA, McDonough SM, Baxter GD, Dempster M, Moore AP: **A descriptive study of the usage of spinal manipulative therapy techniques within a randomized clinical trial in acute low back pain**. *Man Ther* 2005, **10**:61–7.

169. Hurley DA, McDonough SM, Dempster M, Moore AP, Baxter GD: **A randomized clinical trial of manipulative therapy and interferential therapy for acute low back pain**. *Spine.* 2004, **29**(20):2207–16.

170. Hurri H: **The Swedish back school in chronic low back pain. Part II. Factors predicting the outcome**. *Scand J Rehabil Med* 1989, **21**:41–4.

171. Hurwitz EL, Morgenstern H, Harber P, Kominski GF, Belin TR, Yu F, Adams AH: **A randomized trial of medical care with and without physical therapy and chiropractic care with and without physical modalities for patients with low back pain: 6-month follow-up outcomes from the UCLA low back pain study**. *Spine* 2002, **27**(20):2193–204.

172. Iles R, Taylor NF, Davidson M, O'Halloran P: **Telephone coaching can increase activity levels for people with non-chronic low back pain: a randomised trial**. *J Physiother* 2011, **57**(4):231–8.

173. Inoue M, Kitakoji H, Ishizaki N, Tawa M, Yano T, Katsumi Y, Kawakita K: **Relief of low back pain immediately after acupuncture treatment–a randomised, placebo controlled trial**. *Acupunct Med* 2006, **24**(3):103–8.

174. Itoh K, Katsumi Y, Kitakoji H: **Trigger point acupuncture treatment of chronic low back pain in elderly patients–a blinded RCT**. *Acupunct Med* 2004, **22**(4):170–7.

175. Jarvik G J: **Rapid magnetic Resonance imaging vs radiography for patients with low back pain**. *JAMA* 2003, **289**(21).

176. Jellema P, van der Windt DA, van der Horst HE, Twisk JW, Stalman WA, Bouter LM: **Should treatment of (sub)acute low back pain be aimed at psychosocial prognostic factors? Cluster randomised clinical trial in general practice**. *BMJ (Clinical research ed.)* 2005, **331**(7508):84.

177. Johannsen F, Remvig L, Kryger P, Beck P, Warming S, Lybeck K, Dreyer V, Larsen LH: **Exercises for chronic low back pain: a clinical trial.** *J Orthop Sports Phys Ther* 1995, **22**(2):52–59, [http://dx.doi.org/10.2519/jospt.1995.22.2.52].

178. Johnson RE, Jones GT, Wiles NJ, Chaddock C, Potter RG, Roberts C, Symmons DP, Watson PJ, Torgerson DJ, Macfarlane GJ: **Active exercise, education, and cognitive behavioral therapy for persistent disabling low back pain: a randomized controlled trial**. *Spine (Phila Pa 1976)* 2007, **32**(15):1578–85.

179. Jousset N, Fanello S, Bontoux L, Dubus V, Billabert C, Vielle B, Roquelaure Y, Penneau-Fontbonne D, Richard I: **Effects of functional restoration versus 3 hours per week physical therapy: a randomized controlled study**. *Spine* 2004, **29**(5):487–493.

180. Ju WD, Krupa DA, Walters DJ, Newman TL, Borenstein DG, Katz N, Group VCLBPI: **(211) A Placebo-Controlled Trial of Rofecoxib in the Treatment of Chronic Low Back Pain**. *Pain medicine* 2001, **2**(3):242–243.

181. Juni P, Battaglia M, Nuesch E, Hammerle G, Eser P, van Beers R, Vils D, Bernhard J, Ziswiler HR, Dahler M, Reichenbach S, Villiger PM: **A randomised controlled trial of spinal manipulative therapy in acute low back pain**. *Ann Rheum Dis* 2009, **68**(9):1420–7.

182. Kankaanpaa M, Taimela S, Airaksinen O, Hanninen O: **The efficacy of active rehabilitation in chronic low back pain. Effect on pain intensity, self-experienced disability, and lumbar fatigability**. *Spine* 1999, **24**(10):1034–1042.

183. Kapitza KP, Passie T, Bernateck M, Karst M: **First non-contingent respiratory biofeedback placebo versus contingent biofeedback in patients with chronic low back pain: a randomized, controlled, double-blind trial**. *Appl Psychophysiol Biofeedback* 2010, **35**(3):207–17.

184. Katz N, Ju WD, Krupa DA, Sperling RS, Bozalis Rodgers D, Gertz BJ, Gimbel J, Coleman S, Fisher C, Nabizadeh S, Borenstein D: **Efficacy and safety of rofecoxib in patients with chronic low back pain: results from two 4-week, randomized, placebo-controlled, parallel-group, double-blind trials**. *Spine* 2003, **28**(9):851–8; discussion 859.

185. Keel PJ, Wittig R, Deutschmann R, Diethelm U, Knusel O, Loschmann C, Matathia R, Rudolf T, Spring H: **Effectiveness of in-patient rehabilitation for sub-chronic and chronic low back pain by an integrative group treatment program (Swiss Multicentre Study)**. *Scand J Rehabil Med* 1998, **30**(4):211–9.

186. Keijsers JF: **The efficacy of the back school: a randomized trial.** *Arthritis Care Research* 1990, **3**.

187. Keitel W, Frerick H, Kuhn U, Schmidt U, Kuhlmann M, Bredehorst A: **Capsicum pain plaster in chronic non-specific low back pain**. *Arzneimittelforschung* 2001, **51**(11):896–903.

188. Kell RT, Asmundson GJ: **A comparison of two forms of periodized exercise rehabilitation programs in the management of chronic nonspecific low-back pain**. *J Strength Cond Res* 2009, **23**(2):513–23.

189. Kell RT, Risi AD, Barden JM: **The response of persons with chronic nonspecific low back pain to three different volumes of periodized musculoskeletal rehabilitation**. *J Strength Cond Res* 2011, **25**(4):1052–64.

190. Keller S, Ehrhardt-Schmelzer S, Herda C, Schmid S, Basler HD: **Multidisciplinary rehabilitation for chronic back pain in an outpatient setting: a controlled randomized trial**. *Eur J Pain* 1997, **1**(4):279–92.

191. Kellett KM, Kellett DA, Nordholm LA: **Effects of an exercise program on sick leave due to back pain**. *Phys.Ther.* 1991, **71**(4):283–291.

192. Kendrick D: **Radiography of the lumbar spine in primary care patients with low back pain: randomised controlled trial**. *BMJ* 2001, **2001**(322).

193. Kerr DP, Walsh DM, Baxter D: **Acupuncture in the management of chronic low back pain: a blinded randomized controlled trial**. *The Clinical journal of pain.* 2003, **19**(6):364–70.

194. Kerry S: **Routine referral for radiography of patients presenting with low back pain: is patients? outcome influenced by GPs? referral for plain radiography?** *Health Technology Assessment* 2000, **4**(20).

195. Ketenci A, Ozcan E, Karamursel S: **Assessment of efficacy and psychomotor performances of thiocolchicoside and tizanidine in patients with acute low back pain**. *International journal of clinical practice.* 2005, **59**(7):764–70.

196. Klaber Moffett JA, Chase SM, Portek I, Ennis JR: **A controlled, prospective study to evaluate the effectiveness of a back school in the relief of chronic low back pain.** *Spine (Phila Pa 1976)* 1986, **11**(2):120–122.

197. Klein RG, Eek BC, DeLong WB, Mooney V: **A randomized double-blind trial of dextrose-glycerine-phenol injections for chronic, low back pain.** *J Spinal Disord* 1993, **6**:23–33.

198. Kofotolis N, Kellis E: **Effects of two 4-week proprioceptive neuromuscular facilitation programs on muscle endurance, flexibility, and functional performance in women with chronic low back pain**. *Physical therapy.* 2006, **86**(7):1001–12.

199. Kolda, Do, an S, Sonel Tur B, Kurtai, Y, Atay MB: **Comparison of three different approaches in the treatment of chronic low back pain**. *Clin Rheumatol* 2008, **27**(7):873–81.

200. Kole-Snijders AM, Vlaeyen JW, Goossens ME, Rutten-van Molken MP, Heuts PH, van Breukelen G, van Eek H: **Chronic low-back pain: what does cognitive coping skills training add to operant behavioral treatment? Results of a randomized clinical trial**. *J Consult Clin Psychol* 1999, **67**(6):931–44.

201. Kool JP, Oesch PR, Bachmann S, Knuesel O, Dierkes JG, Russo M, de Bie RA, van den Brandt PA: **Increasing days at work using function-centered rehabilitation in nonacute nonspecific low back pain: a randomized controlled trial**. *Archives of physical medicine and rehabilitation.* 2005, **86**(5):857–64.

202. Kool J, Bachmann S, Oesch P, Knuesel O, Ambergen T, de Bie R, van den Brandt P: **Function-centered rehabilitation increases work days in patients with nonacute nonspecific low back pain: 1-year results from a randomized controlled trial**. *Arch Phys Med Rehabil* 2007, **88**(9):1089–94.

203. Koumantakis GA, Watson PJ, Oldham JA: **Trunk muscle stabilization training plus general exercise versus general exercise only: randomized controlled trial of patients with recurrent low back pain**. *Physical therapy.* 2005, **85**(3):209–25.

204. Koumantakis GA, Watson PJ, Oldham JA: **Supplementation of general endurance exercise with stabilisation training versus general exercise only. Physiological and functional outcomes of a randomised controlled trial of patients with recurrent low back pain**. *Clinical biomechanics (Bristol, Avon)* 2005, **20**(5):474–82.

205. Kovacs Fea: **Effect of firmness of mattress on chronic non-specific low-back pain: randomised, double-blind, controlled, multicentre trial**. *Lancet* 2003, **362**.

206. Kovacs FM, Abraira V, Pozo F, Kleinbaum DG, Beltran J, Mateo I, Perez de Ayala C, Pena A, Zea A, Gonzalez-Lanza M, Morillas L: **Local and remote sustained trigger point therapy for exacerbations of chronic low back pain. A randomized, double-blind, controlled, multicenter trial**. *Spine* 1997, **22**(7):786–97.

207. Kroll HR, Kim D, Danic MJ, Sankey SS, Gariwala M, Brown M: **A randomized, double-blind, prospective study comparing the efficacy of continuous versus pulsed radiofrequency in the treatment of lumbar facet syndrome**. *J Clin Anesth* 2008, **20**(7):534–7.

208. Kulisch A, Bender T, Nemeth A, Szekeres L: **Effect of thermal water and adjunctive electrotherapy on chronic low back pain: a double-blind, randomized, follow-up study**. *J Rehabil Med* 2009, **41**:73–9.

209. Kumar S, Negi MP, Sharma VP, Shukla R, Dev R, Mishra UK: **Efficacy of two multimodal treatments on physical strength of occupationally subgrouped male with low back pain**. *J Back Musculoskelet Rehabil* 2009, **22**(3):179–88.

210. Kumar S, Sharma VP, Negi MP: **Efficacy of dynamic muscular stabilization techniques (DMST) over conventional techniques in rehabilitation of chronic low back pain**. *J Strength Cond Res* 2009, **23**(9):2651–9.

211. Kumar S, Sharma VP, Shukla R, Dev R: **Comparative efficacy of two multimodal treatments on male and female sub-groups with low back pain (part II)**. *J Back Musculoskelet Rehabil* 2010, **23**:1–9.

212. Kuukkanen T, Malkia E: **Effects of a three-month therapeutic exercise programme on flexibility in subjects with low back pain**. *Physiother.Res.Int.* 2000, **5**:46–61.

213. Lalanne K, Lafond D, Descarreaux M: **Modulation of the flexion-relaxation response by spinal manipulative therapy: a control group study**. *J Manipulative Physiol Ther* 2009, **32**(3):203–9.

214. Lamb SE, Hansen Z, Lall R, Castelnuovo E, Withers EJ, Nichols V, Potter R, Underwood MR: **Group cognitive behavioural treatment for low-back pain in primary care: a randomised controlled trial and cost-effectiveness analysis**. *Lancet* 2010, **375**(9718):916–23.

215. Lambeek LC, van Mechelen W, Knol DL, Loisel P, Anema JR: **Randomised controlled trial of integrated care to reduce disability from chronic low back pain in working and private life**. *BMJ* 2010, **340**:c1035.

216. Lankhorst GJ, Van de Stadt RJ, Vogelaar TW, Van der Korst JK, Prevo AJ: **The effect of the Swedish Back School in chronic idiopathic low back pain. A prospective controlled study.** *Scand J Rehabil Med* 1983, **15**(3):141–145.

217. Lau PM, Chow DH, Pope MH: **Early physiotherapy intervention in an Accident and Emergency Department reduces pain and improves satisfaction for patients with acute low back pain: a randomised trial**. *Aust J Physiother* 2008, **54**(4):243–9.

218. Leclaire R, Fortin L, Lambert R, Bergeron YM, Rossignol M: **Radiofrequency facet joint denervation in the treatment of low back pain: a placebo-controlled clinical trial to assess efficacy**. *Spine* 2001, **26**(13):1411–6; discussion 1417.

219. Leclaire Rea: **Back School in a First Episode of Compensated Acute Low Back Pain: A Clinical Trial to Assess Efficacy and Prevent Relapse**. *Arch Phys Med Rehabil* 1996, **77**.

220. Leeuw M, Goossens ME, van Breukelen GJ, de Jong JR, Heuts PH, Smeets RJ, Koke AJ, Vlaeyen JW: **Exposure in vivo versus operant graded activity in chronic low back pain patients: results of a randomized controlled trial**. *Pain* 2008, **138**:192–207.

221. Leibing E, Leonhardt U, Koster G, Goerlitz A, Rosenfeldt JA, Hilgers R, Ramadori G: **Acupuncture treatment of chronic low-back pain – a randomized, blinded, placebo-controlled trial with 9-month follow-up**. *Pain* 2002, **96**(1-2):189–96.

222. Lengsfeld M, Konig IR, Schmelter J, Ziegler A: **Passive rotary dynamic sitting at the workplace by office-workers with lumbar pain: a randomized multicenter study**. *Spine Journal* 2007, **7**(5):531–540.

223. Leonhardt C: **TTM-based motivational counselling does not increase physical activity of low back pain patients in a primary care setting?A cluster-randomized controlled trial**. *Patient Education and Counseling* 2008, **70**:50–60.

224. Lewis C, Souvlis T, Sterling M: **Strain-Counterstrain therapy combined with exercise is not more effective than exercise alone on pain and disability in people with acute low back pain: a randomised trial**. *J Physiother* 2011, **57**(2):91–8.

225. Lewis JS, Hewitt JS, Billington L, Cole S, Byng J, Karayiannis S: **A randomized clinical trial comparing two physiotherapy interventions for chronic low back pain**. *Spine.* 2005, **30**(7):711–21.

226. Licciardone JC, Stoll ST, Fulda KG, Russo DP, Siu J, Winn W, Swift J J: **Osteopathic manipulative treatment for chronic low back pain: a randomized controlled trial**. *Spine* 2003, **28**(13):1355–62.

227. Lindstrom I, Ohlund C, Eek C, Wallin L, Peterson LE, Fordyce WE, Nachemson AL: **The effect of graded activity on patients with subacute low back pain: a randomized prospective clinical study with an operant-conditioning behavioral approach**. *Phys.Ther.* 1992, **72**(4):279–290.

228. Lindstrom I, Ohlund C, Nachemson A: **Physical performance, pain, pain behavior and subjective disability in patients with subacute low back pain**. *Scand J Rehabil Med* 1995, **27**(3):153–60.

229. Linton SJ, Bradley LA, Jensen I, Spangfort E, Sundell L: **The secondary prevention of low back pain: a controlled study with follow-up**. *Pain* 1989, **36**(2):197–207.

230. Little P, Lewith G, Webley F, Evans M, Beattie A, Middleton K, Barnett J, Ballard K, Oxford F, Smith P, Yardley L, Hollinghurst S, Sharp D: **Randomised controlled trial of Alexander technique lessons, exercise, and massage (ATEAM) for chronic and recurrent back pain**. *BMJ* 2008, **337**:a884.

231. Little P, Roberts L, Blowers H, Garwood J, Cantrell T, Langridge J, Chapman J: **Should we give detailed advice and information booklets to patients with back pain? A randomized controlled factorial trial of a self-management booklet and doctor advice to take exercise for back pain**. *Spine.* 2001, **26**(19):2065–72.

232. Long A, Donelson R, Fung T: **Does it matter which exercise? A randomized control trial of exercise for low back pain**. *Spine.* 2004, **29**(23):2593–602.

233. Lorig KR, Laurent DD, Deyo RA, Marnell ME, Minor MA, Ritter PL: **Can a Back Pain E-mail Discussion Group improve health status and lower health care costs?: A randomized study**. *Arch Intern Med* 2002, **162**(7):792–6.

234. MacDonald RS, Bell CM: **An open controlled assessment of osteopathic manipulation in nonspecific low-back pain**. *Spine* 1990, **15**(5):364–70.

235. Machado LA, Maher CG, Herbert RD, Clare H, McAuley JH: **The effectiveness of the McKenzie method in addition to first-line care for acute low back pain: a randomized controlled trial**. *BMC Med* 2010, **8**:10.

236. Mackawan S, Eungpinichpong W, Pantumethakul R, Chatchawan U, Hunsawong T, Arayawichanon P: **Effects of traditional Thai massage versus joint mobilization on substance P and pain perception in patients with non-specific low back pain**. *Journal of Bodywork and Movement Therapies* 2007, **11**:9–16.

237. Magnussen L, Strand LI, Skouen JS, Eriksen HR: **Motivating disability pensioners with back pain to return to work - A randomized controlled trial**. *Journal of Rehabilitation Medicine* 2007, **39**:81–87.

238. Manchikanti L, Boswell MV, Rivera JJ, Pampati VS, Damron KS, McManus CD, Brandon DE, Wilson SR: **[ISRCTN 16558617] A randomized, controlled trial of spinal endoscopic adhesiolysis in chronic refractory low back and lower extremity pain**. *BMC Anesthesiology* 2005, **5**:10.

239. Manchikanti L, Rivera JJ, Pampati V, Damron KS, McManus CD, Brandon DE, Wilson SR: **One day lumbar epidural adhesiolysis and hypertonic saline neurolysis in treatment of chronic low back pain: a randomized, double-blind trial**. *Pain Physician* 2004, **7**(2):177–86.

240. Manniche C, Hesselsoe G, Bentzen L, Christensen I, Lundberg E: **Clinical trial of intensive muscle training for chronic low back pain**. *Lancet* 1988, **2**(8626-8627):1473–1476.

241. Mannion AF, Muntener M, Taimela S, Dvorak J: **A randomized clinical trial of three active therapies for chronic low back pain**. *Spine* 1999, **24**(23):2435–2448.

242. Marchand Sea: **Is TENS purely a placebo effect? A controlled study on chronic low back pain**. *Pain* 1993, **54.1**:99–106.

243. Marks RC, Houston T, Thulbourne T: **Facet joint injection and facet nerve block: a randomised comparison in 86 patients with chronic low back pain**. *Pain* 1992, **49**(3):325–8.

244. Marshall P, Murphy B: **Self-report measures best explain changes in disability compared with physical measures after exercise rehabilitation for chronic low back pain.** *Spine* 2008, **33**(3):326–38.

245. Marshall P, Murphy B: **Muscle activation changes after exercise rehabilitation for chronic low back pain.** *Arch Phys Med Rehabil.* 2008, **89**(7):1305–13.

246. Mathews MMJ W; Morkel: **Manipulation and traction for lumbago and sciatica: Physiotherapeutic techniques used in two controlled trials.** *Physiotherapy Practice* 1988, **4**:201–6.

247. Maul I, Laubli T, Oliveri M, Krueger H: **Long-term effects of supervised physical training in secondary prevention of low back pain**. *Eur Spine J* 2005, **14**(6):599–611.

248. Mayer JM, Ralph L, Look M, Erasala GN, Verna JL, Matheson LN, Mooney V: **Treating acute low back pain with continuous low-level heat wrap therapy and/or exercise: a randomized controlled trial**. *The spine journal : official journal of the North American Spine Society.* 2005, **5**(4):395–403.

249. Mayer TG, Gatchel RJ, Keeley J, McGeary D, Dersh J, Anagnostis C: **A randomized clinical trial of treatment for lumbar segmental rigidity**. *Spine* 2004, **29**(20):2199–2205.

250. Mazza M, Mazza O, Pazzaglia C, Padua L, Mazza S: **Escitalopram 20 mg versus duloxetine 60 mg for the treatment of chronic low back pain**. *Expert Opin Pharmacother* 2010, **11**(7):1049–52.

251. Meade TWea: **Low back pain of mechanical origin: randomised comparison of chiropractic and hospital outpatient treatment.** *BMJ* 1990, **300**:1431–7.

252. Melancon B, Miller LH: **Massage therapy versus traditional therapy for low back pain relief: implications for holistic nursing practice**. *Holistic nursing practice.* 2005, **19**(3):116–21.

253. Melzack R, Vetere P, Finch L: **Transcutaneous electrical nerve stimulation for low back pain. A comparison of TENS and massage for pain and range of motion**. *Phys Ther* 1983, **63**(4):489–93.

254. Meng K, Seekatz B, Roband H, Worringen U, Vogel H, Faller H: **Intermediate and long-term effects of a standardized back school for inpatient orthopedic rehabilitation on illness knowledge and self-management behaviors: a randomized controlled trial**. *Clin J Pain* 2011, **27**(3):248–57.

255. Mibielli MA, Geller M, Cohen JC, Goldberg SG, Cohen MT, Nunes CP, Oliveira LB, da Fonseca AS: **Diclofenac plus B vitamins versus diclofenac monotherapy in lumbago: the DOLOR study**. *Curr Med Res Opin* 2009, **25**(11):2589–99.

256. Middleton RS: **A comparison of two analgesic muscle relaxant combinations in acute back pain**. *Br J Clin Pract* 1984, **38**(3):107–9.

257. Miller P, Kendrick D, Bentley E, Fielding K: **Cost-effectiveness of lumbar spine radiography in primary care patients with low back pain**. *Spine.* 2002, **27**(20):2291–7.

258. Million R, Nilsen KH, Jayson MI, Baker RD: **Evaluation of low back pain and assessment of lumbar corsets with and without back supports**. *Ann.Rheum.Dis.* 1981, **40**(5):449–454.

259. Moffett JK, Torgerson D, Bell-Syer S, Jackson D, Llewlyn-Phillips H, Farrin A, Barber J: **Randomised controlled trial of exercise for low back pain: clinical outcomes, costs, and preferences**. *BMJ* 1999, **319**(7205):279–283.

260. Mohseni-Bandpei MA, Critchley J, Staunton T, Richardson B: **A prospective randomised controlled trial of spinal manipulation and ultrasound in the treatment of chronic low back pain**. *Physiotherapy* 2006, **92**:34–42.

261. Mohseni-Bandpei MA, Rahmani N, Behtash H, Karimloo M: **The effect of pelvic floor muscle exercise on women with chronic non-specific low back pain**. *J Bodyw Mov Ther* 2011, **15**:75–81.

262. Molsberger AF, Mau J, Pawelec DB, Winkler J: **Does acupuncture improve the orthopedic management of chronic low back pain: A randomized, blinded, controlled trial with 3 months follow up**. *Pain* 2002, **99**(3):579–587.

263. Moore JE, Von Korff M, Cherkin D, Saunders K, Lorig K: **A randomized trial of a cognitive-behavioral program for enhancing back pain self care in a primary care setting**. *Pain* 2000, **88**(2):145–53.

264. Morone G, Paolucci T, Alcuri MR, Vulpiani MC, Matano A, Bureca I, Paolucci S, Saraceni VM: **Quality of life improved by multidisciplinary back school program in patients with chronic non-specific low back pain: a single blind randomized controlled trial**. *Eur J Phys Rehabil Med* 2011, **47**(4):533–41.

265. Morrison GE, Chase W, Young V, Roberts WL: **Back pain: treatment and prevention in a community hospital**. *Arch Phys Med Rehabil* 1988, **69**(8):605–9.

266. Moseley GL: **Joining forces - combining cognition-targeted motor control training with group or individual pain physiology education: a successful treatment for chronic low back pain**. *Journal of Manual & Manipulative Therapy* 2003, **11**(2):88–94.

267. Moseley GL, Nicholas MK, Hodges PW: **A randomized controlled trial of intensive neurophysiology education in chronic low back pain**. *Clinical Journal of Pain* 2004, **20**(5):324–330.

268. Moseley L: **Combined physiotherapy and education is efficacious for chronic low back pain**. *Aust.J Physiother.* 2002, **48**(4):297–302.

269. Muehlbacher M, Nickel MK, Kettler C, Tritt K, Lahmann C, Leiberich PK, Nickel C, Krawczyk J, Mitterlehner FO, Rother WK, Loew TH, Kaplan P: **Topiramate in treatment of patients with chronic low back pain: a randomized, double-blind, placebo-controlled study**. *Clin J Pain* 2006, **22**(6):526–31.

270. Muller-Schwefe GHH, Uberall MA: **Dysport(registered trademark) for the treatment of myofascial back pain: Results from an open-label, Phase II, randomized, multicenter, dose-ranging study**. *Scandinavian Journal of Pain* 2011, **2**:25–33.

271. Muthukrishnan R, Shenoy SD, Jaspal SS, Nellikunja S, Fernandes S: **The differential effects of core stabilization exercise regime and conventional physiotherapy regime on postural control parameters during perturbation in patients with movement and control impairment chronic low back pain**. *Sports Med Arthrosc Rehabil Ther Technol* 2010, **2**:13.

272. Nadler SF, Steiner DJ, Erasala GN, Hengehold DA, Abeln SB, Weingand KW: **Continuous low-level heatwrap therapy for treating acute nonspecific low back pain.** *Arch Phys Med Rehabil* 2003, **84**(3):329–334, [http://dx.doi.org/10.1053/apmr.2003.50102].

273. Nadler SF, Steiner DJ, Erasala GN, Hengehold DA, Hinkle RT, Beth Goodale M, Abeln SB, Weingand KW: **Continuous low-level heat wrap therapy provides more efficacy than Ibuprofen and acetaminophen for acute low back pain.** *Spine (Phila Pa 1976)* 2002, **27**(10):1012–1017.

274. Nadler SF, Steiner DJ, Petty SR, Erasala GN, Hengehold DA, Weingand KW: **Overnight use of continuous low-level heatwrap therapy for relief of low back pain.** *Arch Phys Med Rehabil* 2003, **84**(3):335–342, [http://dx.doi.org/10.1053/apmr.2003.50103].

275. Nassif H, Brosset N, Guillaume M, Delore-Milles E, Tafflet M, Buchholz F, Toussaint JF: **Evaluation of a randomized controlled trial in the management of chronic lower back pain in a French automotive industry: An observational study**. *Archives of Physical Medicine and Rehabilitation* 2011, **92**(12):1927–1936.e4.

276. Newcomer KL, Vickers Douglas KS, Shelerud RA, Long KH, Crawford B: **Is a videotape to change beliefs and behaviors superior to a standard videotape in acute low back pain? A randomized controlled trial**. *Spine J* 2008, **8**(6):940–7.

277. Newton-John SSD TR; Spence: **Cognitive-behavioural therapy versus EMG biofeedback in the treatment of chronic low back pain.** *Behav Res Ther* 1995, **33**.

278. Nicholas MK, Wilson PH, Goyen J: **Operant-behavioural and cognitive-behavioural treatment for chronic low back pain**. *Behav Res Ther* 1991, **29**(3):225–38.

279. Niemisto L, Lahtinen-Suopanki T, Rissanen P, Lindgren KA, Sarna S, Hurri H: **A randomized trial of combined manipulation, stabilizing exercises, and physician consultation compared to physician consultation alone for chronic low back pain**. *Spine* 2003, **28**(19):2185–91.

280. Niemist L, Rissanen P, Sarna S, Lahtinen ST, Lindgren KA, Hurri H: **Cost-effectiveness of combined manipulation, stabilizing exercises, and physician consultation compared to physician consultation alone for chronic low back pain: A prospective randomized trial with 2-year follow-up**. *Spine* 2005, **30**(10):1109–1115.

281. Nigg BM, Davis E, Lindsay D, Emery C: **The effectiveness of an unstable sandal on low back pain and golf performance**. *Clin J Sport Med* 2009, **19**(6):464–70.

282. Nordeman L, Nilsson B, Moller M, Gunnarsson R: **Early access to physical therapy treatment for subacute low back pain in primary health care: a prospective randomized clinical trial**. *Clin J Pain* 2006, **22**(6):505–11.

283. Norris C, Matthews M: **The role of an integrated back stability program in patients with chronic low back pain**. *Complement Ther Clin Pract* 2008, **14**(4):255–63.

284. Nouwen A: **EMG biofeedback used to reduce standing levels of paraspinal muscle tension in chronic low back pain.** *Pain* 1983, **17**(4):353–360.

285. Oesch PR, Kool JP, Bachmann S, Devereux J: **The influence of a Functional Capacity Evaluation on fitness for work certificates in patients with non-specific chronic low back pain**. *Work (Reading, Mass.)* 2006, **26**(3):259–71.

286. Oleske DM, Lavender SA, Andersson GB, Kwasny MM: **Are back supports plus education more effective than education alone in promoting recovery from low back pain?: Results from a randomized clinical trial**. *Spine (Phila Pa 1976)* 2007, **32**(19):2050–7.

287. Olson AMGea GM; Pellien: **An analysis of the effectiveness of a low back health education program in an employee population.** *Journal of Health Education* 1991, **22**:160–5.

288. Ongley MJ, Klein RG, Dorman TA, Eek BC, Hubert LJ: **A new approach to the treatment of chronic low back pain**. *Lancet* 1987, **2**(8551):143–6.

289. Orava S: **Medical treatment of acute low back pain. Diflunisal compared with indomethacin in acute lumbago**. *Int J Clin Pharmacol Res* 1986, **6**:45–51.

290. Pach D, Brinkhaus B, Roll S, Wegscheider K, Icke K, Willich SN, Witt CM: **Efficacy of injections with Disci/Rhus toxicodendron compositum for chronic low back pain - a randomized placebo-controlled trial**. *PLoS ONE* 2011, **6**(11).

291. Palangio M, Morris E, Doyle RT Jr, Dornseif BE, Valente TJ: **Combination hydrocodone and ibuprofen versus combination oxycodone and acetaminophen in the treatment of moderate or severe acute low back pain.** *Clin Ther* 2002, **24**:87–99.

292. Pallay RM, Seger W, Adler JL, Ettlinger RE, Quaidoo EA, Lipetz R, O'Brien K, Mucciola L, Skalky CS, Petruschke RA, Bohidar NR, Geba GP: **Etoricoxib reduced pain and disability and improved quality of life in patients with chronic low back pain: a 3 month, randomized, controlled trial**. *Scand J Rheumatol* 2004, **33**(4):257–66.

293. Palmieri NF, Smoyak S: **Chronic low back pain: a study of the effects of manipulation under anesthesia**. *Journal of manipulative and physiological therapeutics.* 2002, **25**(8):E8–e17.

294. Paoloni M, Bernetti A, Fratocchi G, Mangone M, Parrinello L, Del Pilar Cooper M, Sesto L, Di Sante L, Santilli V: **Kinesio Taping applied to lumbar muscles influences clinical and electromyographic characteristics in chronic low back pain patients**. *Eur J Phys Rehabil Med* 2011, **47**(2):237–44.

295. Paolucci T, Morone G, Iosa M, Fusco A, Alcuri R, Matano A, Bureca I, Saraceni VM, Paolucci S: **Psychological features and outcomes of the Back School treatment in patients with chronic non-specific low back pain.A randomized controlled study**. *Eur J Phys Rehabil Med* 2011.

296. Peloso PM, Fortin L, Beaulieu A, Kamin M, Rosenthal N: **Analgesic efficacy and safety of tramadol/ acetaminophen combination tablets (Ultracet) in treatment of chronic low back pain: a multicenter, outpatient, randomized, double blind, placebo controlled trial**. *J Rheumatol* 2004, **31**(12):2454–63.

297. Penttinen J, Nevala-Puranen N, Airaksinen O, Jaaskelainen M, Sintonen H, Takala J: **Randomized controlled trial of back school with and without peer support**. *J Occup Rehabil* 2002, **12**:21–9.

298. Perez-Palomares S, Olivan-Blazquez B, Magallon-Botaya R, De-La-Torre-Beldarrain MML, Gaspar-Calvo E, Romo-Calvo L, Garcia-Lazaro R, Serrano-Aparicio B: **Percutaneous electrical nerve stimulation versus dry needling: Effectiveness in the treatment of chronic low back pain**. *Journal of Musculoskeletal Pain* 2010, **18**:23–30.

299. Petersen T, Kryger P, Ekdahl C, Olsen S, Jacobsen S: **The effect of McKenzie therapy as compared with that of intensive strengthening training for the treatment of patients with subacute or chronic low back pain: A randomized controlled trial**. *Spine* 2002, **27**(16):1702–1709.

300. Pipino CLGGPFA F; Menarini, Pizzoli ABASGGR A; Grangie, Cilento F: **A direct myotonolytic (Pridinol Mesilate) for the management of chronic low back pain: A multicentre, comparative clinical evaluation.** *European Journal of Clinical Research* 1991, **1**.

301. Pohjolainen T, Jekunen A, Autio L, Vuorela H: **Treatment of acute low back pain with the COX-2-selective anti-inflammatory drug nimesulide: results of a randomized, double-blind comparative trial versus ibuprofen.** *Spine (Phila Pa 1976)* 2000, **25**(12):1579–1585.

302. Pope MH, Phillips RB, Haugh LD, Hsieh CY, MacDonald L, Haldeman S: **A prospective randomized three-week trial of spinal manipulation, transcutaneous muscle stimulation, massage and corset in the treatment of subacute low back pain**. *Spine* 1994, **19**(22):2571–2577.

303. Powers CM, Beneck GJ, Kulig K, Landel RF, Fredericson M: **Effects of a single session of posterior-to-anterior spinal mobilization and press-up exercise on pain response and lumbar spine extension in people with nonspecific low back pain**. *Phys Ther* 2008, **88**(4):485–493.

304. Preyde M: **Effectiveness of massage therapy for subacute low-back pain: a randomized controlled trial**. *CMAJ.* 2000, **162**(13):1815–1820.

305. Puranik S, Fozard J, Paremain G, Kilminister S, Hughes D, E W: **A randomised, single blind study to evaluate the effects of action potential simulator therapy compared with placebo in patients with chronic back pain**. *The Pain Clinic* 2002, **14**:69–73.

306. Pushpika Attanayake AM, Somarathna KI, Vyas GH, Dash SC: **Clinical evaluation of selected Yogic procedures in individuals with low back pain**. *Ayu* 2010, **31**(2):245–50.

307. Rasmussen J, Laetgaard J, Lindecrona AL, Qvistgaard E, Bliddal H: **Manipulation does not add to the effect of extension exercises in chronic low-back pain (LBP). A randomized, controlled, double blind study**. *Joint Bone Spine* 2008, **75**(6):708–13.

308. Rasmussen-Barr E, Ang B, Arvidsson I, Nilsson-Wikmar L: **Graded exercise for recurrent low-back pain: a randomized, controlled trial with 6-, 12-, and 36-month follow-ups**. *Spine (Phila Pa 1976)* 2009, **34**(3):221–8.

309. Rittweger J, Just K, Kautzsch K, Reeg P, Felsenberg D: **Treatment of chronic lower back pain with lumbar extension and whole-body vibration exercise: a randomized controlled trial**. *Spine* 2002, **27**(17):1829–1834.

310. Ritvanen T, Zaproudina N, Nissen M, Leinonen V, Hanninen O: **Dynamic surface electromyographic responses in chronic low back pain treated by traditional bone setting and conventional physical therapy**. *J Manipulative Physiol Ther* 2007, **30**:31–7.

311. Roberts L, Little P, Chapman J, Cantrell T, Pickering R, Langridge J: **The back home trial: general practitioner-supported leaflets may change back pain behavior**. *Spine* 2002, **27**(17):1821–8.

312. Roche G, Ponthieux A, Parot-Shinkel E, Jousset N, Bontoux L, Dubus V, Penneau-Fontbonne D, Roquelaure Y, Legrand E, Colin D, Richard I, Fanello S: **Comparison of a functional restoration program with active individual physical therapy for patients with chronic low back pain: a randomized controlled trial**. *Arch Phys Med Rehabil* 2007, **88**(10):1229–35.

313. Roche-Leboucher G, Petit-Lemanac'h A, Bontoux L, Dubus-Bausiere V, Parot-Shinkel E, Fanello S, Penneau-Fontbonne D, Fouquet N, Legrand E, Roquelaure Y, Richard I: **Multidisciplinary intensive functional restoration versus outpatient active physiotherapy in chronic low back pain: a randomized controlled trial**. *Spine (Phila Pa 1976)* 2011, **36**(26):2235–42.

314. van der Roer N, van Tulder M, Barendse J, Knol D, van Mechelen W, de Vet H: **Intensive group training protocol versus guideline physiotherapy for patients with chronic low back pain: a randomised controlled trial**. *Eur Spine J* 2008, **17**(9):1193–200.

315. Rose M, Reilly J, Penny B, al e: **Chronic low back pain rehabilitation programs: a study of the optimum duration of treatment and a comparison of group and individual therapy.** *Spine* 1997, **22**.

316. Rossignol M, Allaert FA, Rozenberg S, Valat JP, Avouac B, Peres G, Le Teuff G, Bannwarth B: **Measuring the contribution of pharmacological treatment to advice to stay active in patients with subacute low-back pain: a randomised controlled trial**. *Pharmacoepidemiology and drug safety.* 2005, **14**(12):861–7.

317. Rozenberg S, Delval C, Rezvani Y, Olivieri Apicella N, Kuntz JL, Legrand E, Valat JP, Blotman F, Meadeb J, Rolland D, Hary S, Duplan B, Feldmann JL, Bourgeois P: **Bed rest or normal activity for patients with acute low back pain: a randomized controlled trial**. *Spine.* 2002, **27**(14):1487–93.

318. Ruoff GE, Rosenthal N, Jordan D, Karim R, Kamin M: **Tramadol/acetaminophen combination tablets for the treatment of chronic lower back pain: a multicenter, randomized, double-blind, placebo-controlled outpatient study**. *Clin Ther* 2003, **25**(4):1123–41.

319. Sahin N, Albayrak I, Durmus B, Ugurlu H: **Effectiveness of back school for treatment of pain and functional disability in patients with chronic low back pain: a randomized controlled trial**. *J Rehabil Med* 2011, **43**(3):224–9.

320. Salzmann WPGGM E; Pforringe: **Treatment of chronic low-back syndrome with tetrazepam in a placebo controlled double-blind trial.** *J Drug Dev* 1992, **4**.

321. Sanders GE, Reinert O, Tepe R, Maloney P: **Chiropractic adjustive manipulation on subjects with acute low back pain: visual analog pain scores and plasma beta-endorphin levels.** *J Manipulative Physiol Ther* 1990, **13**(7):391–395.

322. Santaella Da Fonseca Lopes De Sousa K, Garcia Orfale A, Mara Meireles S, Roberto Leite J, Natour J: **Assessment of a biofeedback program to treat chronic low back pain**. *Journal of Musculoskeletal Pain* 2009, **17**(4):369–377.

323. Sator Katzenschlager SM, Scharbert G, Kozek Langenecker SA, Szeles JC, Finster G, Schiesser AW, Heinze G, Kress HG: **The short- and long-term benefit in chronic low back pain through adjuvant electrical versus manual auricular acupuncture**. *Anesthesia and analgesia.* 2004, **98**(5):1359–64, table of contents.

324. Schattenkirchner M, Milachowski KA: **A double-blind, multicentre, randomised clinical trial comparing the efficacy and tolerability of aceclofenac with diclofenac resinate in patients with acute low back pain**. *Clinical rheumatology.* 2003, **22**(2):127–35.

325. Schiltenwolf M, Buchner M, Heindl B, Reumont J, Mller A, Eich W: **Comparison of a biopsychosocial therapy (BT) with a conventional biomedical therapy (MT) of subacute low back pain in the first episode of sick leave: a randomized controlled trial**. *Eur Spine J.* 2006, **15**(7):1083–92.

326. Schnitzer TJ, Gray WL, Paster RZ, Kamin M: **Efficacy of tramadol in treatment of chronic low back pain**. *J Rheumatol* 2000, **27**(3):772–8.

327. Senna MK, Machaly SA: **Does maintained spinal manipulation therapy for chronic nonspecific low back pain result in better long-term outcome?** *Spine (Phila Pa 1976)* 2011, **36**(18):1427–37.

328. Shabat S, Gefen T, Nyska M, Folman Y, Gepstein R: **The effect of insoles on the incidence and severity of low back pain among workers whose job involves long-distance walking**. *European spine journal : official publication of the European Spine Society, the European Spinal Deformity Society, and the European Section of the Cervical Spine Research Society.* 2005, **14**(6):546–50.

329. Shankar N, Thakur M, Tandon OP, Saxena AK, Arora S, Bhattacharya N: **Autonomic status and pain profile in patients of chronic low back pain and following electro acupuncture therapy: a randomized control trial**. *Indian J Physiol Pharmacol* 2011, **55**:25–36.

330. Sherman KJ, Cherkin DC, Erro J, Miglioretti DL, Deyo RA: **Comparing yoga, exercise, and a self-care book for chronic low back pain: a randomized, controlled trial**. *Annals of internal medicine.* 2005, **143**(12):849–56.

331. Sherman KJ, Cherkin DC, Wellman RD, Cook AJ, Hawkes RJ, Delaney K, Deyo RA: **A randomized trial comparing yoga, stretching, and a self-care book for chronic low back pain**. *Arch Intern Med* 2011, **171**(22):2019–26.

332. Shirado O, Doi T, Akai M, Hoshino Y, Fujino K, Hayashi K, Marui E, Iwaya T: **Multicenter randomized controlled trial to evaluate the effect of home-based exercise on patients with chronic low back pain: the Japan low back pain exercise therapy study**. *Spine (Phila Pa 1976)* 2010, **35**(17):E811–9.

333. da Silva AG, de Sousa CP, Koehler J, Fontana J, Christo AG, Guedes-Bruni RR: **Evaluation of an extract of Brazilian arnica (Solidago chilensis Meyen, Asteraceae) in treating lumbago**. *Phytother Res* 2010, **24**(2):283–7.

334. Skljarevski V, Ossanna M, Liu-Seifert H, Zhang Q, Chappell A, Iyengar S, Detke M, Backonja M: **A double-blind, randomized trial of duloxetine versus placebo in the management of chronic low back pain**. *Eur J Neurol* 2009, **16**(9):1041–8.

335. Skljarevski V, Zhang S, Chappell AS, Detke MJ, Murray I, Backonja M: **Maintenance of effect of duloxetine in patients with chronic low back pain**. *European Journal of Pain* 2009, **13**:S195.

336. Skljarevski V, Zhang S, Desaiah D, Alaka KJ, Palacios S, Miazgowski T, Patrick K: **Duloxetine versus placebo in patients with chronic low back pain: a 12-week, fixed-dose, randomized, double-blind trial**. *J Pain* 2010, **11**(12):1282–90.

337. Skljarevski V, Zhang S, Desaiah D, Palacios S, Miazgowski T, Patrick K: **Efficacy and safety of duloxetine 60 mg once-daily in patients with chronic low back pain**. *Journal of Pain* 2010, **11**(4):S38.

338. Sokunbi O, Watt P, Moore A: **Changes in plasma concentration of serotonin in response to spinal stabilisation exercises in chronic low back pain patient**. *Nig Q J Hosp Med* 2007, **17**(3):108–11.

339. Soukup MG, Glomsrod B, Lonn JH, Bo K, Larsen S: **The effect of a Mensendieck exercise program as secondary prophylaxis for recurrent low back pain. A randomized, controlled trial with 12-month follow-up**. *Spine* 1999, **24**(15):1585–91; discussion 1592.

340. Sonne M, Christensen K, Hansen SE, Jensen EM: **Injection of steroids and local anaesthetics as therapy for low-back pain.** *Scand J Rheumatol* 1985, **14**(4):343–345.

341. Sorensen PH, Bendix T, Manniche C, Korsholm L, Lemvigh D, Indahl A: **An educational approach based on a non-injury model compared with individual symptom-based physical training in chronic LBP. A pragmatic, randomised trial with a one-year follow-up**. *BMC Musculoskelet Disord* 2010, **11**:212.

342. Soriano R F; Rios: **Gallium Arsenide Laser Treatment of chronic low back pain: a prospective randomised and double blind study.** *Laser Therapy* 1998, **10**.

343. Spinhoven P, Ter Kuile M, Kole-Snijders AM, Hutten Mansfeld M, Den Ouden DJ, Vlaeyen JW: **Catastrophizing and internal pain control as mediators of outcome in the multidisciplinary treatment of chronic low back pain**. *Eur J Pain* 2004, **8**(3):211–9.

344. Staal JB, Hlobil H, Twisk JW, Smid T, Koke AJ, van MW: **Graded activity for low back pain in occupational health care: a randomized, controlled trial**. *Ann.Intern.Med* 2004, **140**(2):77–84.

345. Stankovic R, Johnell O: **Conservative treatment of acute low-back pain. A prospective randomized trial: McKenzie method of treatment versus patient education in "mini back school"**. *Spine* 1990, **15**(2):120–3.

346. Steenstra IA, Anema JR, van Tulder MW, Bongers PM, de Vet HC, van Mechelen W: **Economic evaluation of a multi-stage return to work program for workers on sick-leave due to low back pain**. *J Occup Rehabil* 2006, **16**(4):557–78.

347. Storheim K, Brox JI, Holm I, Koller AK, Bo K: **Intensive group training versus cognitive intervention in sub-acute low back pain: short-term results of a single-blind randomized controlled trial**. *J Rehabil Med* 2003, **35**(3):132–40.

348. Strong Ja: **Incorporating Cognitive-Behavioral Therapy with Occupational Therapy: A Comparative Study with Patients with Low Back Pain**. *Journal of Occupational Rehabilitation* 1998, **V8**:61–71.

349. Suen LK, Wong TK, Chung JW, Yip VY: **Auriculotherapy on low back pain in the elderly**. *Complement Ther Clin Pract* 2007, **13**:63–9.

350. Suni J, Rinne M, Natri A, Statistisian MP, Parkkari J, Alaranta H: **Control of the lumbar neutral zone decreases low back pain and improves self-evaluated work ability: a 12-month randomized controlled study**. *Spine* 2006, **31**(18):E611–20.

351. Sweetman BJ, Baig A, Parsons DL: **Mefenamic acid, chlormezanone-paracetamol, ethoheptazine-aspirin-meprobamate: a comparative study in acute low back pain**. *Br J Clin Pract* 1987, **41**(2):619–24.

352. Szczurko O, Cooley K, Busse JW, Seely D, Bernhardt B, Guyatt GH, Zhou Q, Mills EJ: **Naturopathic care for chronic low back pain: a randomized trial**. *PLoS One* 2007, **2**(9):e919.

353. Tao XG, Bernacki EJ: **A randomized clinical trial of continuous low-level heat therapy for acute muscular low back pain in the workplace**. *J Occup Environ Med* 2005, **47**(12):1298–306.

354. Tefner IK, Nemeth A, Laszlofi A, Kis T, Gyetvai G, Bender T: **The effect of spa therapy in chronic low back pain: a randomized controlled, single-blind, follow-up study**. *Rheumatol Int* 2011.

355. Thomas KJ, MacPherson H, Ratcliffe J, Thorpe L, Brazier J, Campbell M, Fitter M, Roman M, Walters S, Nicholl JP: **Longer term clinical and economic benefits of offering acupuncture care to patients with chronic low back pain**. *Health technology assessment (Winchester, England)* 2005, **9**(32):iii–iv, ix–x, 1–109.

356. Thomas KJ, MacPherson H, Thorpe L, Brazier J, Fitter M, Campbell MJ, Roman M, Walters SJ, Nicholl J: **Randomised controlled trial of a short course of traditional acupuncture compared with usual care for persistent non-specific low back pain**. *BMJ: British Medical Journal* 2006, **333**(7569):1–6.

357. Toda Y: **Impact of waist/hip ratio on the therapeutic efficacy of lumbosacral corsets for chronic muscular low back pain**. *Journal of orthopaedic science : official journal of the Japanese Orthopaedic Association.* 2002, **7**(6):644–9.

358. Triano JJ, McGregor M, Hondras MA, Brennan PC: **Manipulative therapy versus education programs in chronic low back pain**. *Spine* 1995, **20**(8):948–55.

359. Tritilanunt T, Wajanavisit W: **The efficacy of an aerobic exercise and health education program for treatment of chronic low back pain**. *J Med Assoc.Thai.* 2001, **84 Suppl 2**:S528–S533.

360. Tsao H, Hodges PW: **Immediate changes in feedforward postural adjustments following voluntary motor training**. *Exp Brain Res* 2007, **181**(4):537–46.

361. Turner JA: **Comparison of group progressive-relaxation training and cognitive-behavioral group therapy for chronic low back pain**. *J Consult Clin Psychol* 1982, **50**(5):757–65.

362. Turner JA, Clancy S: **Comparison of operant behavioral and cognitive-behavioral group treatment for chronic low back pain**. *J Consult Clin Psychol* 1988, **56**(2):261–6.

363. Turner JA, Clancy S, McQuade KJ, Cardenas DD: **Effectiveness of behavioral therapy for chronic low back pain: a component analysis**. *J Consult Clin.Psychol.* 1990, **58**(5):573–579.

364. Turner JA, Jensen MP: **Efficacy of cognitive therapy for chronic low back pain**. *Pain* 1993, **52**(2):169–77.

365. Tzn F, Unalan H, Oner N, Ozgzel H, Kirazli Y, I asio, lu A, Kuran B, Tzn S, Ba, ar G: **Multicenter, randomized, double-blinded, placebo-controlled trial of thiocolchicoside in acute low back pain**. *Joint, bone, spine : revue du rhumatisme.* 2003, **70**(5):356–61.

366. Underwood Mea: **United Kingdom back pain exercise and manipulation (UK BEAM) randomised trial: Effectiveness of physical treatments for back pain in primary care**. *British Medical Journal* 2004, **329**(7479):1377–1381.

367. Underwood MR, Morgan J: **The use of a back class teaching extension exercises in the treatment of acute low back pain in primary care**. *Fam Pract* 1998, **15**:9–15.

368. Unsgaard-Tondel M, Fladmark AM, Salvesen O, Vasseljen O: **Motor control exercises, sling exercises, and general exercises for patients with chronic low back pain: a randomized controlled trial with 1-year follow-up**. *Phys Ther* 2010, **90**(10):1426–40.

369. Valle-Jones JC, Walsh H, O'Hara J, O'Hara H, Davey NB, Hopkin-Richards H: **Controlled trial of a back support ('Lumbotrain') in patients with non-specific low back pain**. *Curr.Med Res.Opin.* 1992, **12**(9):604–613.

370. Vasseljen O, Fladmark AM: **Abdominal muscle contraction thickness and function after specific and general exercises: a randomized controlled trial in chronic low back pain patients**. *Man Ther.* 2010, **15**(5):482–9.

371. Vasseljen O, Unsgaard-Tondel M, Westad C, Mork PJ: **Effect of Core Stability Exercises on Feedforward Activation of Deep Abdominal Muscles in Chronic Low Back Pain: A Randomized Controlled Trial**. *Spine (Phila Pa 1976)* 2011.

372. Verbeek Jea: **Early Occupational Health Management of Patients with Back Pain**. *Spine* 2002, **27**(17).

373. Videman T, Heikkila J, Partanen T: **Double-blind parallel study of meptazinol versus diflunisal in the treatment of lumbago**. *Curr Med Res Opin* 1984, **9**(4):246–52.

374. Vlaeyen JW, Haazen IW, Schuerman JA, Kole-Snijders AM, van Eek H: **Behavioural rehabilitation of chronic low back pain: comparison of an operant treatment, an operant-cognitive treatment and an operant-respondent treatment**. *Br J Clin Psychol* 1995, **34 (Pt 1)**:95–118.

375. Vollenbroek Hutten MM, Hermens HJ, Wever D, Gorter M, Rinket J, Ijzerman MJ: **Differences in outcome of a multidisciplinary treatment between subgroups of chronic low back pain patients defined using two multiaxial assessment instruments: the multidimensional pain inventory and lumbar dynamometry**. *Clinical rehabilitation.* 2004, **18**(5):566–79.

376. Von Korff M, Balderson BH, Saunders K, Miglioretti DL, Lin EH, Berry S, Moore JE, Turner JA: **A trial of an activating intervention for chronic back pain in primary care and physical therapy settings**. *Pain.* 2005, **113**(3):323–30.

377. Von Korff M, Moore JE, Lorig K, Cherkin DC, Saunders K, Gonzalez VM, Laurent D, Rutter C, Comite F: **A randomized trial of a lay person-led self-management group intervention for back pain patients in primary care**. *Spine* 1998, **23**(23):2608–15.

378. Vong SK, Cheing GL, Chan F, So EM, Chan CC: **Motivational enhancement therapy in addition to physical therapy improves motivational factors and treatment outcomes in people with low back pain: a randomized controlled trial**. *Arch Phys Med Rehabil* 2011, **92**(2):176–83.

379. Waagen Gea: **Short term trial of chiropractic adjustments for the relief of chronic low back pain**. *Manual Medicine* 1986, **2**.

380. Wand BM, Bird C, McAuley JH, Dore CJ, MacDowell M, De Souza LH: **Early intervention for the management of acute low back pain: a single-blind randomized controlled trial of biopsychosocial education, manual therapy, and exercise**. *Spine* 2004, **29**(21):2350–6.

381. Weiner DK, Perera S, Rudy TE, Glick RM, Shenoy S, Delitto A: **Efficacy of percutaneous electrical nerve stimulation and therapeutic exercise for older adults with chronic low back pain: a randomized controlled trial**. *Pain* 2008, **140**(2):344–57.

382. Weiner DK, Rudy TE, Glick RM, Boston JR, Lieber SJ, Morrow LA, Taylor S: **Efficacy of Percutaneous Electrical Nerve Stimulation for the Treatment of Chronic Low Back Pain in Older Adults**. *Journal of the American Geriatrics Society* 2003, **51**(5):599–608.

383. Wetherell JL, Afari N, Rutledge T, Sorrell JT, Stoddard JA, Petkus AJ, Solomon BC, Lehman DH, Liu L, Lang AJ, Hampton Atkinson J: **A randomized, controlled trial of acceptance and commitment therapy and cognitive-behavioral therapy for chronic pain**. *Pain* 2011, **152**(9):2098–2107.

384. White PF, Ghoname EA, Ahmed HE, Hamza MA, Craig WF, Vakharia AS: **The effect of montage on the analgesic response to percutaneous neuromodulation therapy**. *Anesth Analg* 2001, **92**(2):483–7.

385. Whitfill T, Haggard R, Bierner SM, Pransky G, Hassett RG, Gatchel RJ: **Early intervention options for acute low back pain patients: a randomized clinical trial with one-year follow-up outcomes**. *J Occup Rehabil* 2010, **20**(2):256–63.

386. Wiesel SW, Cuckler JM, Deluca F, Jones F, Zeide MS, Rothman RH: **Acute low-back pain. An objective analysis of conservative therapy**. *Spine* 1980, **5**(4):324–30.

387. van Wijk RM, Geurts JW, Wynne HJ, Hammink E, Buskens E, Lousberg R, Knape JT, Groen GJ: **Radiofrequency denervation of lumbar facet joints in the treatment of chronic low back pain: a randomized, double-blind, sham lesion-controlled trial**. *The Clinical journal of pain.* 2005, **21**(4):335–44.

388. Williams K, Abildso C, Steinberg L, Doyle E, Epstein B, Smith D, Hobbs G, Gross R, Kelley G, Cooper L: **Evaluation of the effectiveness and efficacy of Iyengar yoga therapy on chronic low back pain**. *Spine (Phila Pa 1976)* 2009, **34**(19):2066–76.

389. Williams NH, Wilkinson C, Russell I, Edwards RT, Hibbs R, Linck P, Muntz R: **Randomized osteopathic manipulation study (ROMANS): Pragmatic trial for spinal pain in primary care**. *Family Practice* 2003, **20**(6):662–669.

390. Witt CM, Jena S, Selim D, Brinkhaus B, Reinhold T, Wruck K, Liecker B, Linde K, Wegscheider K, Willich SN: **Pragmatic randomized trial evaluating the clinical and economic effectiveness of acupuncture for chronic low back pain**. *American Journal of Epidemiology* 2006, **164**(5):487–496.

391. Worth SGA, Henry SM, Bunn JY: **Real-time ultrasound feedback and abdominal hollowing exercises for people with low back pain**. *NZ Journal of Physiotherapy* 2007, **35**:4–11.

392. Wright A, Lloyd Davies A, Williams S, Ellis R, Strike P: **Individual active treatment combined with group exercise for acute and subacute low back pain**. *Spine.* 2005, **30**(11):1235–41.

393. Wu JX, Wang B: **Early intervention of aerobic exercise to rehabilitation of non-specific low back pain**. *Zhongguo Linchuang Kangfu* 2004, **8**(26):5718–20.

394. Yelland MJ, Glasziou PP, Bogduk N, Schluter PJ, McKernon M: **Prolotherapy Injections, Saline Injections, and Exercises for Chronic Low-Back Pain: A Randomized Trial**. *Spine* 2004, **29**:9–16.

395. Yeung CK, Leung MC, Chow DH: **The use of electro-acupuncture in conjunction with exercise for the treatment of chronic low-back pain**. *Journal of alternative and complementary medicine (New York, N.Y.)* 2003, **9**(4):479–90.

396. Yildirim Y, Soyunov S: **Relationship between learning strategies of patients and proper perception of the home exercise program with non-specific low back pain**. *J Back Musculoskelet Rehabil* 2010, **23**(3):137–42.

397. Yip YB, Tse SH: **The effectiveness of relaxation acupoint stimulation and acupressure with aromatic lavender essential oil for non-specific low back pain in Hong Kong: a randomised controlled trial**. *Complementary therapies in medicine.* 2004, **12**:28–37.

398. Yokoyama Mea: **Comparison of Percutaneous Electrical Nerve Stimulation with Transcutaneous Electrical Nerve Stimulation for Long- Term Pain Relief in Patients with Chronic Low Back Pain**. *Anesth Analg* 2004, **98**.

399. Yozbatiran N, Yildirim Y, Parlak B: **Effects of fitness and aquafitness exercises on physical fitness in patients with chronic low back pain**. *The Pain Clinic* 2004, **16**:35–42.

400. Zaringhalam J, Manaheji H, Rastqar A, Zaringhalam M: **Reduction of chronic non-specific low back pain: a randomised controlled clinical trial on acupuncture and baclofen**. *Chin Med* 2010, **5**:15.

401. Zerbini C, Ozturk ZE, Grifka J, Maini M, Nilganuwong S, Morales R, Hupli M, Shivaprakash M, Giezek H: **Efficacy of etoricoxib 60 mg/day and diclofenac 150 mg/day in reduction of pain and disability in patients with chronic low back pain: results of a 4-week, multinational, randomized, double-blind study**. *Curr Med Res Opin* 2005, **21**(12):2037–49.
