# Supplementary material for: A Systematic Review of Outcome Measures Use, Analytical Approaches, Reporting Methods, and Publication Volume by Year in Low Back Pain Trials Published between 1980 and 2012: Respice, adspice, et prospice
Source: PLoS One. 2016 Oct 24;11(10):e0164573. doi: 10.1371/journal.pone.0164573 (PMC5077121; doi:10.1371/journal.pone.0164573)
Supplement: S2 Table — A table showing the characteristics of excluded trials and their references. (PDF) [file pone.0164573.s003.pdf]

Excluded trial characteristics and references - see manuscript for rejection codes (in Table 1)

Lead author	Year	Title	Rejection code
Ansari N. N.	2006	A randomized, single blind placebo controlled clinical trial on the effect of continuous ultrasound on low back pain	1
Jans M. P	2006	Intermittent follow-up treatment with Cesar exercise therapy in patients with subacute or chronic aspecific low back pain: results of a randomized, controlled trial with a 1.5-year follow-up	1
Natour J	2002	Loxoprofen in the treatment of low back pain - clinical efficacy and safety in comparison to diclofenac	1
Ruth M	2010	Laser acupuncture for chronic back pain. A double-blind clinical study	1
Blazek M	1986	Comparative study of biarison and voltaren in acute lumbar pain and lumbo-ischialia	2
Brown K	1991	Cost-effectiveness of a back school intervention for municipal employees	2
Buchbinder R	2001	Population based intervention to change back pain beliefs and disability: three part evaluation	2
Buenaver L	2006	Cognitive-behavioral self-help for chronic pain	2
Buynak	2009	Dose Stability Of Tapentadol ER For The Relief Of Chronic Low Back Pain: Results Of A Randomized, Active And PlaceboControlled Study	2
Buynak R	2010	Erratum: Efficacy and safety of tapentadol extended release for the management of chronic low back pain: results of a prospective, randomized, double-blind, placebo- and active-controlled Phase III study (Expert Opin. Pharmacother. (2010) 11 (1787-1804))	2
Celestini M	2005	A randomized controlled trial on the efficacy of physical exercise in patients braced for instability of the lumbar spine	2
Clark D	2010	Tolerance and opioid-induced hyperalgesia in clinical populations	2
Codding C	2009	Efficacy and Safety Evaluation of 12 Weeks Extended-Release Hydrocodone/Acetaminophen Treatment in Patients with Chronic Low Back Pain (CLBP) by Prior Opioid Use	2
Cohen S	2008	Lumbar zygapophysial (facet) joint radiofrequency denervation success as a function of pain relief during diagnostic medial branch blocks: a multicenter analysis	2
Corey D	1996	A limited functional restoration program for injured workers: a randomized trial	2
Cox J M	2009	Letter to the Editor: A randomized controlled trial comparing 2 types of spinal manipulation and minimal conservative medal care for adults 5s years and older with sub-acute or chronic low back pain	2
Dagenais S	2007	Prolotherapy injections for chronic low-back pain (Review)	2
Demoulin C	2006	Benefits of a Physical Training Program After Back School for Chronic Low Back Pain Patients	2
Deshpande A	2007	Opioids for chronic low-back pain	2
Engers A	2008	Individual patient education for low back pain	2
Etropolski M	2009	A randomized, double-blind, placebo- and active-con- trolled phase III study of tapentadol ER for chronic low back pain: analysis of efficacy endpoint sensitivity	2
Evans D D	2010	Characteristics and Predictors of Short-Term Outcomes in Individuals Self-selecting Yoga or Physical Therapy for Treatment of Chronic Low Back Pain	2
Flynn T	2006	The audible pop for high-velocity thrust manipulation and outcome in individuals with low back pain	2
George S Z	2009	Psychosocial education improves low back pain beliefs: results from a cluster randomized clinical trial (NCT00373009) in a primary prevention setting	2
George S Z	2010	Comparison of Graded Exercise and Graded Exposure Clinical Outcomes for Patients With Chronic Low Back Pain	2
George S Z	2011	Brief psychosocial education, not core stabilization, reduced incidence of low back pain: results from the Prevention of Low Back Pain in the Military (POLM) cluster randomized trial	2
Gerner P	2003	Topical amitriptyline in healthy volunteers	2
Hagg O	2003	The clinical importance of changes in outcome scores after treatment for chronic low back pain	2
Hancock M.J	2010	Letter	2
Hasegawa TM	2009	Acupuncture For Acute NonSpecific Low Back Pain: A Randomized, Controlled, Placebo Trial	2
Helmhout P	2008	Isolated lumbar extensor strengthening versus regular physical therapy in an army working population with nonacute low back pain: a randomized controlled trial	2
Helmhout P	2010	Prognostic factors for perceived recovery or functional improvement in non-specific low back pain: secondary analyses of three randomized clinical trials	2
Henchos Y	2010	Cost-utility analysis of a three-month exercise programme vs usual care following multidisciplinary rehabilitation for chronic low back pain	2
Herzog W	1991	Effects of different treatment modalities on gait symmetry and clinical measures for sacroiliac joint patients	2
Hides J A	2011	Effect of Motor Control Training on Muscle Size and Football Games Missed from Injury	2
Holm I	2002	Fusion surgery is slightly better than non-surgical treatment in patients with severe chronic low back pain	2
Hsieh C	1992	Functional outcomes of low back pain: comparison of four treatment groups in a randomised controlled trial	2
Hubley-kozey C	2002	Muscle activation during exercises to improve truck stability in men with low back pain	2

Hush J	2006	TENS of unknown value in the treatment of chronic low back pain	2
Ijzelenberg H	2007	Effectiveness of a back pain prevention program: A cluster randomized controlled trial in an occupational setting	2
Katz N	2009	Tanezumab, an Anti-Nerve Growth Factor (NGF) antibody, for the treatment of chronic low back pain (CLBP) – a ran- domized, controlled, double-blind, phase 2 trial	2
Kavanagh S	2009	Tapentadol extended release (ER) for chronic low back pain: results of Euroqol-5 dimension (EQ-5D) and Short Form-36 (SF-36) health status questionnaires	2
Keijsers J	1989	A back school in the Netherlands: evaluating the results	2
Kinalski R	1989	The comparison of the results of manual therapy versus physiotherapy methods used in treatment of patients with low back pain syndromes	2
Lee JW	2010	Therapeutic Trial of Fluoroscopic Interlaminar Epidural Steroid Injection for Axial Low Back Pain: Effectiveness and Outcome Predictors	2
Lee TJ	2011	Pharmacologic treatment for low back pain: One component of pain care	2
Leichtfried	2010	Can bright light therapy ameliorate symptoms associated with low back pain (LBP)? A randomized controlled trial	2
Liddle D	2007	Advice for the management of low back pain: A systematic review of randomised controlled trials	2
Lierz P	2004	Comparison between bupivacaine 0.125% and ropivacaine 0.2% for epidural administration to outpatients with chronic low back pain	2
Loeser J	2004	Prolotherapy Injections, Saline Injections, and Exercises for Chronic Low-Back Pain: A Randomized Trial - Point of View	2
Loisel P	2002	Discriminative and predictive validity assessment of the Quebeck task force classification	2
Long A	2008	The comparative prognostic value of directional preference and centralization: A useful tool for front-line clinicians?	2
Macdonald A	1983	Superficial acupuncture in the relief of chronic low back pain	2
Macfarlane G	2008	Changing patient perceptions of their illness: Can they contribute to an improved outcome for episodes of musculoskeletal pain?	2
Machado L	2005	The McKenzie method for the management of acute non-specific low back pain: design of a randomized controlled trial	2
Magnusson	2008	Motor Control Learning in Chronic Low Back Pain	2
Mandara A	2008	A randomised controlled trial on the effectiveness of osteopathic manipulative treatment of chronic low back pain	2
Mehling W	2006	Breath therapy for chronic low back pain	2
Mooney V	2003	RE Aure, 2003	2
Najm W	2008	German acupuncture trials (GERAC) for chronic low back pain	2
Ney J	2006	Treatment of chronic low back pain with successive injections of botulinum toxin A over 6 Months	2
O`Donnel JB	2009	The Effectiveness of a Weak Opioid Medication versus a Cyclo-oxygenase-2 (COX-2) Selective Non-steroidal Anti-inflammatory Drug in Treating Flare-up of Chronic Low-back Pain: Results from Two Randomized, Double-blind, 6-week Studies	2
O'Brien N	2006	Randomised, controlled trial comparing physiotherapy and Pilates in the treatment of ordinary low back pain	2
Padua R	2009	Re (letter): A random- ized study of back school in women with chronic low back pain. Quality of life at three, six, and twelve months follow-up.	2
Peniston JH	2009	Oxymorphone Extended Release for the Treatment of Chronic Low Back Pain: A Retrospective Pooled Analysis of Enriched-Enrollment Clinical Trial Data Stratified According to Age, Sex, and Prior Opioid Use	2
Perrot S	2006	Efficacy and Tolerability of Paracetamol/Tramadol (325 mg/37.5 mg) Combination Treatment Compared with Tramadol (50 mg) Monotherapy in Patients with Subacute Low Back Pain: A Multicenter, Randomized, Double-Blind, Parallel-Group, 10-Day Treatment Study	2
Petrofsky J	2008	Improving the outcomes after back injury by a core muscle strengthening program	2
Podichetty V	2008	Re: Oleske D M, Lavender S A, Andersson G B, et al. Are back supports plus education more effective than education alone in promoting recovery from low back pain? Results from a randomized clinical trial	2
Ralph L	2009	Improvement in Functional Status with Carisoprodol 250-mg Tablets in Patients with Acute Lower Back Spasm: A Randomized, Double-blind, Placebo-controlled Trial	2
Rivero-Arias O	2006	Cost-Utility Analysis of Physiotherapy Treatment Compared With Physiotherapy Advice in Low Back Pain	2
Schmidt-Wilcke T	2006	Affective components and intensity of pain correlate with structural differences in gray matter in chronic back pain patients	2
Schwarz I	2007	Relative responsiveness of 3 different types of clinical outcome measures on chiropractic patients with low back pain	2
Seroussi R	2003	Effectiveness of percutaneous neuromodulation therapy for patients with chronic and severe low back pain	2
Shakoor A	2010	Evaluation of the effects of selective rehabilitation on the patients with chronic low back pain	2
Shum G	2006	Movement coordination of the lumbar spine and hip during a picking up activity in low back pain subjects	2
Skljarevski V	2010	Effect of Duloxetine 60mg Once Daily Versus Placebo in Patients with Chronic Low Back Pain: A 12-Week, Randomized, Double-Blind Trial	2

Smeets R	2008	Treatment expectancy and credibility are associated with the outcome of both physical and cognitive-behavioral treatment in chronic low back pain	2
Smeets R	2008	Chronic low back pain: Physical training, graded activity with problem solving training, or both? The one-year post-treatment results of a randomized controlled trial	2
Smeets R	2009	Do lumbar stabilising exercises reduce pain and disability in patients with recurrent low back pain?	2
Tobis J	1983	Musculoskeletal manipulation in the treatment of low back pain	2
Underwood M	2011	Predicting Response to a Cognitive–Behavioral Approach to Treating Low Back Pain: Secondary Analysis of the BeST Data Set	2
Vickers A	2004	Statistical reanalysis of four recent randomized trials of acupuncture for pain using analysis of covariance	2
Walter D	2004	RE Yelland M, Prolotherapy injections, saline injections and exercises for chronic low-back pain: a randomized trial	2
Warming S	2008	Little effect of transfer technique instruction and physical fitness training in reducing low back pain among nurses: a cluster randomised intervention study	2
Wheeler WJ	2010	Functional status of patients with acute low back pain following treatment with carisoprodol 250-mg tablets assessed by the roland-morris disability questionnaire (RMDQ)	2
Whitman J	2004	The influence and specialty certifications on clinical outcomes for patients with low back pain treated within a standardized physical therapy management program	2
Zaina F	2010	Clinical and kinematic evaluation of osteopathy vs specific exercises in obese non-specific chronic low back pain females patients: a randomized controlled trial	2
Brinkhaus B	2003	Acupuncture randomized trials (ART) in patients with chronic low back pain and osteoarthritis of the knee - design and protocols	3
Carson J	2005	Loving-kindness meditation for chronic low back pain	3
Cevik R	2007	Effect of new traction technique of prone position on distraction of lumbar vertebrae and its relation with different application of heating therapy in low back pain	3
Cleland J	2006	Comparison of the effectiveness of three manual physical therapy techniques in a subgroup of patients with low back pain who satisfy a clinical prediction rule: study protocol of a randomized controlled trial	3
Galantino M	2004	The impact of modified hatha yoga on chronic low back pain: a pilot study	3
Haas M	2004	Dose-response for chiropractic care of chronic low back pain	3
Hall T	2006	Mulligan bent leg raise technique - a preliminary randomized trial of immediate effects after a single intervention	3
Hurley D	2009	A walking programme and a supervised exercise class versus usual physiotherapy for chronic low back pain: a single-blinded randomised controlled trial. (The Supervised Walking In comparison to Fitness Training for Back Pain (SWIFT) Trial)	3
Mehling W	2005	Randomized controlled trial of breath therapy for patients with chronic low back pain	3
Menzel M	2006	Interventions to reduce back pain in rehabilitation hospital nursing staff	3
Menzel N	2006	Back pain in direct patient care providers: Early interventions with cognitive behavioral Therapy	3
Pozo-Cruz B	2011	Effects of whole body vibration therapy on main outcome measures for chronic non-specific low back pain: a single blind Randomized controlled trial	3
Shaughnessy M	2004	A pilot study to investigate the effect of lumbar stabilisation exercise training on function ability and quality of life in patients with chronic low back pain	3
Smith A	2007	The effect of the Feldenkrais method on pain and anxiety in people experiencing chronic low back pain	3
Tsukayama H	2002	Randomised controlled trial comparing the effectiveness of electroacupuncture and TENS for low back pain: a preliminary study for a pragmatic trial	3
Westrom K	2010	Individualized chiropractic and integrative care for low back pain: the design of a randomized clinical trial using a mixed-methods approach	3
Williams A	2005	Effect of Iyengar yoga therapy for chromic low back pain	3
Wilson E	2003	Muscle energy technique in patients with acute low back pain: a pilot clinical trial	3
Etropolski ME	2010	Dose Conversion Between Tapentadol Immediate and Extended Release for Low Back Pain	4
Hamza M	1999	Effect of the duration of electrical stimulation on the analgesic response in pain	4
Itoh K	2006	Effects of trigger point acupuncture on chronic low back pain in elderly patients	4
Katz J	2005	A randomized, placebo-controlled trial of bupropion sustained release in chronic low back pain	4
Lewis C	2010	A randomised controlled study examining the short-term effects of StraineCounterstrain treatment on quantitative sensory measures at digitally tender points in the low back	4
Meng C	2003	Acupuncture for chronic low back pain in older patients: a randomized, controlled trial	4
Rashiq S	2003	The effect of opioid analgesia on exercise test performance in chronic low back pain	4
Vlaeyen J	2002	The treatment of fear of movement/(Re)injury in chronic low back pain: evidence on the effectiveness of exposure in vivo	4
Adamczyk A	2009	Effectiveness of holistic Physiotherapyfor low back pain	5
Alcoff J	1982	Controlled trial of imipramine for chronic low back pain	5

Arbus L	1990	Activity of tetrazepam in low back pain	5
Arden N	2005	A multicentre randomized controlled trial of epidural corticosteroid injections for sciatica: the WEST study	5
Arkuszewski Z	1986	The efficacy of manual treatment in low back pain: a clinical trial	5
Arts M	2006	Cost-effectiveness of microendoscopy discectomy versus conventional open discectomy in the treatment of lumbar disk herniation: a prospective randomised controlled trial	5
Atkinson J	1998	A placebo-controlled randomized clinical trial of nortriptyline for chronic low back pain	5
Atkinson J	1999	Effects of noradrenergic and serotonergic antidepressants on chronic low back pain intensity	5
Basler HD	2006	TTM-based counselling in physiotherapy does not contribute to an increase of adherence to activity recommendations in older adults with chronic low back pain--a randomised controlled trial	5
Basmajian J	1988	Acute back pain and spasm a controlled multicenter trial of combined analgesic and antispasm agents	5
Belavy D L	2010	Countermeasures against lumbar spine deconditioning in prolonged bed rest: resistive exercise with and without whole body vibration	5
Bendix A	1996	Multidisciplinary intensive treatment for chronic low back pain: a randomized, prospective study	5
Bendix A	1998	A prospective, randomized 5-year follow-up study of functional restoration in chronic low back pain patients	5
Bendix T	2000	Functional restoration versus outpatient physical training in chronic low back pain	5
Bergholdt K	2008	Better Backs by Better Beds?	5
Berry H	1988	A multicenter placebo-controlled study in general practice to evaluate the efficacy and safety of tizanidine in acute low-back pain	5
Blomberg S	1994	A randomized study of manual therapy with steroid injections in low-back pain	5
Bogefeldt J	2007	Sick leave reductions from a comprehensive manual therapy programme for low back pain:the Gotland Low Back Pain Study	5
Bonetti M	2005	Interforaminal O2-O3 versus periradicular steroidal infiltrations in lower back pain: randomized controlled study	5
Borenstein D	1990	Cyclobenzaprine and naproxen versus naproxen alone in the treatment of acute low back pain and muscle spasm	5
Bronfort G	1989	Chiropractic versus general medical treatment of low back pain: A small scale controlled clinical trial	5
Bronfort G	2011	Supervised exercise, spinal manipulation, and home exercise for chronic low back pain: a randomized clinical trial	5
Browder A	2007	Effectiveness of an Extension-Oriented Treatment Approach in a Subgroup of Subjects With Low Back Pain:A Randomized Clinical Trial	5
Bru E	1994	Effects of different psychological interventions of neck, shoulder and low back pain in female hospital staff	5
Buhrman M	2003	Controlled trial of internet-based treatment with telephone support for chronic back pain	5
Buttagat,V	2011	The immediate effects of traditional Thai massage on heart rate variability and stress-related parameters in patients with back pain associated with myofascial trigger points	5
Carette S	1991	A controlled trial of corticosteroid injections into facet joints for chronic low back pain	5
Carlsson C	2001	Acupuncture for chronic low back pain: A randomized placebo-controlled study with long-term follow-up	5
Carter J	2006	The effects of stability ball training on spinal stability in sedentary individuals	5
Chair S	2003	Effect of positioning on back pain after coronary angiography	5
Christensen F	2002	Circumferential lumbar spinal fusion with brantigan cage versus posterolateral fusion with titanium cotrel-dubousset instrumentation	5
Christiansen S	2010	A short goal-pursuit intervention to improve physical capacity: A randomized clinical trial in chronic back pain patients	5
Coan R	1980	The acupuncture treatment of low back pain: A randomized controlled study	5
Corcoll J	2006	Implementation of neuroreflexotherapy for subacute and chronic neck and back pain within the spanish public health system: audit results after one year	5
Costantino C	2011	Mesotherapy versus Systemic Therapy in the Treatment of Acute Low Back Pain: A Randomized Trial	5
Critchley D	2007	Effectiveness and Cost-Effectiveness of Three Types of Physiotherapy Used to Reduce Chronic Low Back Pain Disability	5
Curnow D	2009	Altered motor control, posture and the Pilates method of exercise prescription	5
Day I	2008	Can Topical Anesthetic Reduce the Pain Associated with Diagnostic Blocks of the Lumbosacral Spine?	5
Desmoulin G	2007	Initial results using Khan Kinetic Treatment(trademark) as a low back pain treatment option	5
Dickens C	2000	The relationship between pain and depression in a trial using paroxetine in sufferers of chronic low back pain	5
Donaldson S	1994	Randomized study of the application of single motor unit biofeedback training to chronic low back pain	5

Eisenberg D	2007	Addition of choice of complementary therapies to usual care for acute low back pain: a randomized controlled trial	5
Enthoven P	2004	Clinical course in patients seeking primary care for back or neck pain: a prospective 5-year course follow-up of outcome and health care consumption with subgroup analysis	5
Erhard R	1994	Relative effectiveness of an extension program and a combined program of manipulation and flexion and extension exercises in patients with acute low back syndrome	5
Evans C	1987	A randomized controlled trial of flexion exercises, education and bed rest for patients with acute low back pain	5
Evans D	1980	Medicines of choice in low back pain	5
Faas A	1993	A randomized, placebo-controlled trial of exercise therapy in patients with acute low back pain	5
Facci LM	2011	Effects of transcutaneous electrical nerve stimulation (TENS) and interferential currents (IFC) in patients with nonspecific chronic low back pain: randomized clinical trial	5
Fanello S	2002	Evaluation of a training program for the prevention of lower back pain among hospital employees	5
Farrell J	1982	Acute low back pain. Comparison of two conservative treatment approaches	5
Foster L	2001	Botulinum toxin A and chronic low back pain: a randomized, double-blind study	5
Friedrich M	1998	Combined exercise and motivation program: effect on the compliance and level of disability of patients with chronic low back pain: a randomized controlled trial	5
Fritz J	2003	Comparison of classification-based physical therapy with therapy based on clinical practice guidelines for patients with acute low back pain	5
Fritz J	2008	Is There a Subgroup of Patients With Low Back Pain Likely to Benefit From Mechanical Traction?	5
Fritzell P	2002	Chronic low back pain and fusion: a comparison of three surgical techniques	5
Gale G	2006	Infrared therapy for chronic low back pain: a randomized, controlled trial	5
Gatti R	2011	Efficacy of trunk balance exercises for individuals with chronic low back pain: a randomized clinical trial	5
Gemignani G	1991	Transcutanious electrical nerve stimulation in ankylosing spondylitis: a double-blind study	5
George S	2008	A Randomized Trial of Behavioral Physical Therapy Interventions for Acute and Sub-Acute Low Back Pain	5
Giles L	2003	Chronic spinal pain	5
Giles L	2006	Chronic spinal pain syndromes: a clinical pilot trial comparing acupuncture, a nonsteroidal anti-inflammatory drug and spinal manipulation	5
Glaser J	2001	Electrical muscle stimulation as an adjunct to exercise therapy in the treatment of nonacute low back pain: a randomized trial	5
Godfrey C	1984	A randomized trial of manipulation for low-back pain in a medical setting	5
Goodkin K	1990	A randomized, double-blind, placebo-controlled trial of trazodone hydrochloride in chronic low back pain syndrome	5
Grunnesjo M	2003	A randomized controlled clinical trial of stay-active care versus manual therapy in addition to stay-active care: functional variables and pain	5
Hale M	2005	Efficacy and safety of oxymorphone extended release in chronic low back pain: Results of a randomized, double-blind, placebo- and active-controlled phase III study	5
Hale M	2007	Efficacy and Safety of OPANA ER (Oxymorphone Extended Release) for Relief of Moderate to Severe Chronic Low Back Pain in Opioid-Experienced Patients: A 12-Week, Randomized, Double-blind, placebo-controlled Study	5
Hale M	2010	Once-daily OROS hydromorphone ER compared with placebo in opioid-tolerant patients with chronic low back pain	5
Helmhout P	2004	Comparison of a high-intensity and a low intensity lumbar extensor training program as minimal intervention treatment in low back pain: a randomized trial	5
Hemmila H	1997	Does folk medicine work? A randomized clinical trial on patients with prolonged back pain	5
Heymans M	2006	Does flexion-distraction help treat chronic low back pain?	5
Hurley D	2001	Interferential therapy electrode placement technique in acute low back pain: a preliminary investigation	5
Ikegami S	2010	Anti-Nociceptive Effects of Elcatonin Injection for Postmenopausal Women with Back Pain: A Randomized Controlled Trial	5
Inoue M	2010	Comparison of the effectiveness of acupuncture treatment and local anaesthetic injection for low back pain: a randomized controlled trial.	5
Jamison R	1998	Opioid therapy for chronic noncancer back pain. A randomized prospective study	5
Jensen I	2005	A 3-year follow-up of a multidisciplinary rehabilitation programme for back and neck pain	5
Kaapa E	2006	Multidisciplinary group rehabilitation versus individual physiotherapy for chronic nonspecific low back pain. A randomized trial.	5
Kasis AG	2009	Significantly Improved Outcomes With a Less Invasive Posterior Lumbar Interbody Fusion Incorporating Total Facetectomy	5
Katz N	2007	A 12-week, randomized, placebo-controlled trial assessing the safety and efficacy of oxymorphone extended release for opioid naive patients with chronic low back pain	5
Kettenmann B	2007	Impact of Continuous Low Level Heatwrap Therapy in Acute Low Back Pain Patients: Subjective and Objective Measurements	5

Kleinbohl D	2006	Amantadine sulphate reduces experimental sensitization and pain in chronic back pain patients	5
Koes B	1992	Randomised clinical trial of manipulative therapy and physiotherapy for persistent back and neck complaints: results of a one year follow-up	5
Koes B	1992	A blinded randomized clinical trial of manual therapy and physiotherapy for chronic back and neck complaints: physical outcome measures	5
Koes B	1992	The effectiveness of manual therapy, physiotherapy, and treatment by general practitioner for nonspecific back and neck complaints	5
Kominski F	2005	Economic evaluation of four treatments for low back pain	5
Konrad K	1992	Controlled trial of balneotherapy in treatment of low back pain	5
Kovacs F	2002	Effectiveness and cost-effectiveness analysis of neuroreflexotherapy for sub acute and chronic low back pain in routine general practice	5
Kovacs F	2007	A Comparison of Two Short Education Programs for Improving Low Back Pain-Related Disability in the Elderly	5
Kucera M	2005	Topical symphytum herb concentrate cream against myalgia: a randomized controlled double-blind clinical study	5
Kullich W	2006	Additional outcome improvement in the rehabilitation of chronic low back pain after nuclear resonance therapy	5
Kullich W	2006	The effect of MBST-Nuclear Resonance Therapy with a complex 3-dimensional electromagnetic nuclear resonance field on patients with low back pain	5
Lang E	2003	Multidisciplinary rehabilitation versus usual care for chronic low back pain in the community: effects on quality of life	5
Lee P	2006	Efficacy of pulsed electromagnetic therapy for chronic lower back pain: a randomized, double-blind, placebo-controlled study	5
Lehmann T	1986	Efficacy of electro acupuncture and TENS in the rehabilitation of chronic low back pain patients	5
Lilius G	1989	lumbar facet joint syndrome	5
Limke J	2008	Randomized trial comparing the effects of one set vs two sets of resistance exercises for outpatients with chronic low back pain and leg pain	5
Lin M	2010	A comparison between pulsed radiofrequency and electro-acupuncture for relieving pain in patients with chronic low back pain	5
Lindstrom I	1992	Mobility, strength and fitness after a graded activity program for patients with sub acute low back pain	5
Loisel P	1997	A population based, randomized clinical trial on back pain management	5
Magnussen L	2005	Effect of a brief cognitive training programme in patients with long-lasting back pain evaluated as unfit for surgery	5
Malmivaara A	2006	The treatment of acute low back pain - bed rest, exercises or ordinary activity?	5
Manniche C	1991	Intensive dynamic back exercises for chronic low back pain: a clinical trial	5
Matsumo S	1991	Clinical evaluation of ketoprofen (orudis) in lumbago- a double-blind comparison with diclofenac sodium	5
Mattila R	2007	The Effects of Lifestyle Intervention for Hypertension on Low Back Pain	5
Mayer J	2006	Continuous low-level heat wrap therapy for the prevention and early phase treatment of delayed-onset muscle soreness of the low back: A randomized controlled trial	5
McIlveen B	1998	A randomised controlled study of the outcome of hydrotherapy for subjects with low back or back and leg pain	5
Mehta S	2009	Evaluation of eperisone hydrochloride in the treatment of acute musculoskeletal spasm associated with low back pain: A randomized, double-blind, placebo-controlled trial	5
Milgrom C	2005	A controlled randomized study of the effect of training with orthoses on the incidence of weight bearing induced back pain among infantry recruits	5
Mirovsky Y	2006	The effect of ambulatory lumbar traction combined with treadmill on patients with chronic low back pain	5
Miyazaki S	2009	Applicability of Press Needles to a Double-blind Trial<br>A Randomized, Double-blind, Placebo-controlled Trial	5
Modic M	2005	Acute low back pain and radiculopathy: MR Imaging findings and their prognostic role and effect on outcome	5
Moffett JK	2006	Randomized trial of two physiotherapy interventions for primary care neck and back pain patients: 'McKenzie' vs brief physiotherapy pain management	5
Mullican W	2001	Tramadol/acetaminophen combination tablets and codeine/acetaminophen combination capsules for the management of chronic pain: a comparative trial	5
Murtezani A	2011	A comparison of high intensity aerobic exercise and passive modalities for the treatment of workers with chronic low back pain: a randomized, controlled trial	5
Nath S	2008	Percutaneous Lumbar Zygapophysial (Facet) Joint<br><br>Neurotomy Using Radiofrequency Current, in the Management of Chronic Low Back Pain	5
Nelson-Wong E	2010	Changes in muscle activation patterns and subjective low back pain ratings during prolonged standing in response to an exercise intervention	5
North R	2002	Spinal cord stimulation electrode design: prospective, randomized, controlled trial comparing percutaneous and laminectomy electrodes - part 1: technical outcomes	5
North R	2007	Spinal Cord Stimulation With Interleaved Pulses: A Randomized, Controlled Trial	5
Nouwen A	2006	Effects of focusing and distraction on cold pressure induced pain on chronic back pain and control subjects	5

Nuhr M	2004	Active warming during emergency transport relieves acute low back pain	5
Paatelma M	2008	Orthopaedic manual therapy, McKenzie method or advice only for low back pain in working adults: a randomized controlled trial with one year follow-up	5
Pal B	1986	A controlled trial of continuous lumbar traction in the treatment of back pain and sciatica	5
Pareek A	2009	Aceclofenac–tizanidine in the treatment of acute low back pain: a double-blind, double-dummy, randomized, multicentric, comparative study against aceclofenac alone	5
Pengel L	2007	Physiotherapist-directed exercise, advice, or both for sub acute low back pain: a randomized trial	5
Petersen T	2007	One-year follow-up comparison of the effectiveness of McKenzie treatment and strengthening training for patients with chronic low back pain: outcome and prognostic factors	5
Petersen T	2011	The McKenzie Method Compared With Manipulation When Used Adjunctive to Information and Advice in Low Back Pain Patients Presenting With Centralization or Peripheralization	5
Pneumaticos S	2006	Low back pain: Prediction of short-term outcome of facet joint injection with bone scintigraphy	5
Popovic D	2009	Lumbar Stimulation Belt for Therapy of Low-Back Pain	5
Portenoy R	2007	Fentanyl buccal tablet (FBT) for relief of breakthrough pain in opioid-treated patients with chronic low back pain: a randomized, placebo-controlled study	5
Postacchini F	1988	Efficacy of various forms of conservative treatment in low back pain	5
Quartana P	2007	Attentional strategy moderates effects of pain catastrophizing on symptom-specific physiological responses in chronic low back pain patients	5
Ralph L	2008	Double-blind, placebo-controlled trial of carisoprodol 250-mg tablets in the treatment of acute lower-back spasm	5
Rasmussen-Barr E	2003	Stabilizing training compared with manual treatment in sub-acute and chronic low-back pain	5
Rauck RL	2006	A randomized, open-label study of once-a-day AVINZA (morphine sulphate extended-release capsules) versus twice-a-day OxyContin (oxycodone hydrochloride controlled release tablets) for chronic low back pain: the extension phase of the ACTION trial	5
Risch S	1993	Lumbar strengthening in chronic low back pain patients	5
Romano C	2009	Pregabalin, celecoxib, and their combination for treatment of chronic low-back pain	5
Rusinyol F	2009	Effect of two different doses of eperisone in the treatment of acute low back pain	5
Rydeard R	2006	Pilates-based therapeutic exercise: effect on subjects with nonspecific chronic low back pain and functional disability: A randomized controlled trial	5
Sasso R	2004	A prospective, randomized controlled clinical trial of anterior lumbar interbody fusion using a titanium cylindrical threaded fusion device	5
Scheel I	2002	Blind faith? The effects of promoting active sick leave for back pain patients	5
Schimmel J	2009	No effect of traction in patients with low back pain: a single centre, single blind, randomized controlled trial of Intervertebral Differential Dynamics Therapy.	5
Serfer GT	2010	Randomized, double-blind trial of carisoprodol 250 mg compared with placebo and carisoprodol 350 mg for the treatment of low back spasm	5
Serferlis T	1998	Conservative treatment in patients sick-listed for acute low-back pain: a prospective randomized study with 12 months follow-up	5
Sertpoyraz F	2009	Comparison of isokinetic exercise versus standard exercise training in patients with chronic low back pain: a randomized controlled study	5
Sherman KJ	2010	Treatment Expectations and Preferences as Predictors of Outcome of Acupuncture for Chronic Back Pain	5
Sherry E	2001	A prospective randomized controlled study of VAX-D and TENS for the treatment of chronic low back pain	5
Shimoji K	2007	Pain relief by transcutaneous electric nerve stimulation with bidirectional modulated sine waves in patients with chronic back pain: a randomized, double-blind, sham-controlled study	5
Skargren E	1997	Cost and effectiveness analysis of chiropractic and physiotherapy treatment for low back pain and neck pain: Six month follow-up	5
Skargren E	1998	One-year follow-up comparison of the cost and effectiveness of chiropractic and physiotherapy as primary management for back pain: subgroup analysis, recurrence, and additional health care utilization	5
Skouen J	2002	Relative cost-effectiveness of extensive and light multidisciplinary treatment programs versus treatment as usual for patients with chronic low back pain on long term sick leave	5
Slater M	2009	Preventing Progression to Chronicity in First Onset, Sub acute Low Back Pain: An Exploratory Study	5
Smeets R	2006	Reduction of Pain Catastrophizing Mediates the Outcome of Both Physical and Cognitive-Behavioral Treatment in Chronic Low Back Pain	5
Smeets R	2006	Active rehabilitation for chronic low back pain: Cognitive-behavioral, physical, or both? First direct post-treatment results from a randomized controlled trial [ISRCTN22714229]	5
Soonawalla D	2008	Efficacy of thiocolchicoside in Indian patients suffering from low back pain associated with muscle spasm	5

Spratt K	1993	Efficacy of flexion and extension treatments incorporating braces for low-back pain patients with retrodisplacement, spondylolisthesis or normal sagittal translation	5
Standaert C	2002	Bed rest or continuation of activity for low back pain	5
Steenstra IA	2006	The effectiveness of graded activity for low back pain in occupational healthcare	5
Stein D	1996	The efficacy of amitriptyline and acetaminophen in the management of acute low back pain	5
Steiner D	2009	The efficacy and safety of buprenorphine transdermal system (BTDS) in subjects with moderate to severe low back pain: A double-blind study	5
Steiner D	2011	Efficacy and Safety of Buprenorphine Transdermal System (BTDS) for Chronic Moderate to Severe Low Back Pain: A Randomized, Double-Blind Study	5
Steiner D	2011	Efficacy and Safety of the Seven-Day Buprenorphine Transdermal System in Opioid-Naive Patients with Moderate to Severe Chronic Low Back Pain: An Enriched, Randomized, Double-Blind, Placebo-Controlled Study	5
Stuckey S	1986	EMG biofeedback training, relaxation training, and placebo for the relief of chronic back pain	5
Subin B	2003	Treatment of chronic low back pain by local injection of botulinum toxin-A	5
Sutlive T	2009	Comparison of short-term response to two spinal manipulation techniques for patients with low back pain in a military beneficiary population.	5
Tasleem R	2003	Chronic low back pain - comparative analysis of treatment response to drugs and different physical modalities	5
Tavafian S	2007	Low back pain education and short term quality of life: a randomized trial	5
Tavafian S	2008	A Randomized Study of Back School in Women With Chronic Low Back Pain	5
Tavafian S	2011	Treatment of Chronic Low Back Pain A Randomized Clinical Trial Comparing Multidisciplinary Group-based Rehabilitation Program and Oral Drug Treatment With Oral Drug Treatment Alone	5
Tekur P	2008	Effect of Short-Term Intensive Yoga Program on Pain, Functional Disability, and Spinal Flexibility in Chronic Low Back Pain: A Randomized Control Study	5
Tekur P	2010	Effect of yoga on quality of life of CLBP patients: A randomized control study	5
Thomas M	1994	Importance of modes of acupuncture in the treatment of chronic nociceptive low back pain	5
Tilbrook H	2011	Yoga for Chronic Low Back Pain	5
Torstensen T	1998	Efficacy and costs of medical therapy, conventional physiotherapy, and self-exercise in patients with chronic low back pain. A pragmatic, randomized, single-blinded, controlled trial with 1-year follow-up	5
Toya S	1994	Report on a computer randomized double blind clinical trial to determine the effectiveness of the GaAIAs (830nm) diode laser for pain attenuation in selected groups	5
Tsui M	2004	The effectiveness of electro acupuncture versus electrical heat acupuncture in the management of chronic low-back pain	5
Waterworth R	1985	An open study of diflunisal, conservative and manipulative therapy in the management of low back pain	5
Weber H	1984	Traction therapy in patients with herniated lumbar intervertebral discs	5
Werners R	1999	Randomized trial comparing interferential therapy with motorized lumbar traction and massage in the management of low back pain in a primary care setting	5
Wilkey A	2008	A Comparison Between Chiropractic Management and Pain Clinic Management for Chronic Low-Back Pain in a National Health Service Outpatient Clinic	5
Barker K	2008	Treatment of chronic back pain by sensory discrimination training. A Phase I RCT of a novel device (FairMed) vs. TENS	6
Cabitza P	2008	Efficacy and safety of eperisone in patients with low back pain: a double blind randomized study	6
Li C	2008	Analgesic efficacy and tolerability of flupirtine vs. tramadol in patients with sub acute low back pain: a double-blind multicentre trial	6
Yakhno N	2006	Analgesic Efficacy and Safety of Lornoxicam Quick-Release Formulation Compared with Diclofenac Potassium Randomised, Double-Blind Trial in Acute Low Back Pain	6
Cambron J	2006	One-year follow-up of a randomized clinical trial comparing flexion with an exercise program for chronic low-back pain	7
Casserley-Feeney S	2007	The ACCESS trial -- randomised controlled trial of public hospital-based versus private clinic-based physiotherapy for low back pain: clinical outcomes	7
Clauw D	2006	Is acupuncture more effective than sham acupuncture in relieving pain in patients with low back pain?	7
Cole C	2001	Does acupuncture or massage work in people with persistent back pain	7
Fritzell P	2004	Cost-effectiveness of lumbar fusion and nonsurgical treatment for chronic low back pain in the Swedish lumbar spine study a multicenter, randomized controlled trial from the Swedish lumbar spine study group	7
Frost H	1998	A fitness programme for patients with chronic low back pain: 2-year follow-up of a randomised controlled trial	7
Gilbert F	2004	Does early imaging influence management and improve outcome in patients with low back pain? A pragmatic randomised controlled trial	7
Goossens M	1998	Health economic assessment of behavioral rehabilitation in chronic low back pain: A randomised clinical trial	7
Goossens M	2004	Treatment expectancy affects the outcome of cognitive-behavioral interventions in chronic pain	7

Gould E	2009	The Pain Quality Response Profile of Oxymorphone Extended Release in the Treatment of Low Back Pain	7
Hlobil H	2005	The effects of a graded activity intervention for low back pain in occupational health on sick leave, functional status and pain: 12 month results of a randomized controlled trial	7
Hlobil H	2007	Substantial sick-leave costs savings due to a graded activity intervention for workers with non-specific sub-acute low back pain	7
Hollinghurst S	2008	Randomised controlled trial of Alexander technique lessons, exercise, and massage (ATEAM) for chronic and recurrent back pain: economic evaluation	7
Hurwitz E	2002	The effectiveness of physical modalities among patients with low back pain randomized to chiropractic care: Findings from the UCLA low back pain study	7
Hurwitz E	2005	Effects of recreational physical activity and back exercises on low back pain and psychological distress: findings from the UCLA low back pain study	7
Hurwitz E	2006	A randomized trial of Chiropractic and medical care for patients with low back pain	7
Karjalainen K	2003	Mini intervention for sub acute low back pain	7
Karjalainen K	2004	Mini-intervention for sub acute low back pain. Two-year follow-up and modifiers of effectiveness	7
Katz N	2004	Onset of pain relief with rofecoxib in chronic low back pain: results of two four-week, randomized, placebo-controlled trials	7
Kendrick D	2001	The role of radiography in primary care patients with low back pain of at least 6 weeks duration: a randomised (unblinded) controlled trial	7
Kerry S	2002	Radiography for low back pain: a randomised controlled trial and observational study in primary care	7
Lamb S	2010	A multicentred randomised controlled trial of a primary care-based cognitive behavioural programme for low back pain. The Back Skills Training (BeST) trial	7
Linton S	2006	A 5-year follow-up evaluation of the health and economic consequences of an early cognitive behavioral intervention for back pain: a randomized controlled trial	7
Mannion A	2001	Active therapy for chronic low back pain. part 1 effects on back muscle activation, fatigueability and strength	7
Meade T	1995	Randomised comparison of chiropractic and hospital outpatient management for low back pain: results from extended follow up	7
Mellin G	1989	A controlled study on the outcome of inpatient and outpatient treatment of low back pain. Part II. Effects on physical measurements three months after treatment	7
Mellin G	1990	A controlled study on the outcome of inpatient and outpatient treatment of low back pain	7
Muller R	2005	Long-term follow-up of a randomized clinical trial assessing the efficacy of medication, acupuncture and spinal manipulation for chronic mechanical spinal pain syndromes	7
Ratcliffe J	2006	A randomized controlled trial of acupuncture care for persistent low back pain: cost effectiveness analysis	7
Rauck R	2006	The ACTION study: a randomized, open-label, multicenter trial comparing once-a-day extended-release morphine sulphate capsules (AVINZA) to twice-a-day controlled release oxycodone hydrochloride tablets (OxyContin) for the treatment of chronic, moderate to severe low back pain	7
Rivero-Arias O	2005	Surgical stabilisation of the spine compared with a programme of intensive rehabilitation for the management of patients with chronic low back pain: cost utility analysis based on a randomized controlled trial	7
Rivero-Arias O	2006	Cost-utility Analysis of Physiotherapy Treatment Compared With Physiotherapy Advise in Low Back Pain	7
Sherman K	2009	Characteristics of patients with chronic back pain who benefit from acupuncture	7
Skargren E	1998	Predictive factors for 1-year outcome of low-back and neck pain in patients treated in primary care: comparison between the treatment strategies chiropractic and physiotherapy	7
Snook S	2002	The reduction of chronic, nonspecific low back pain through the control of early morning lumbar flexion: 3-year follow-up	7
Strong L	2006	Cost-effectiveness of two self-care interventions to reduce disability associated with back pain	7
Videbaek T	2006	Circumferential fusion improves outcome in comparison with instrumented posteriolateral fusion: Long-term results of a randomized clinical trial	7
Whitehurst D	2007	A Brief Pain Management Program Compared With Physical Therapy for Low Back Pain: Results From an Economic Analysis Alongside a Randomized Clinical Trial	7
Wilson-MacDonald J	2008	The MRC spine stabilization trial: surgical methods, outcomes, costs, and complications of surgical stabilization	7
Yu F	2003	Use of a Markov transition model to analyse longitudinal low-back pain data	7

[1–336]

References

1. North RB, Kidd DH, Olin JC, Sieracki JM: **Spinal cord stimulation electrode design: prospective, randomized, controlled trial comparing percutaneous and laminectomy electrodes-part I: technical outcomes.** *Neurosurgery* 2002, **51**(2):381–9; discussion 389–90.
2. Karjalainen K: **Mini-Intervention for Subacute Low Back Pain Two-Year Follow-up and Modifiers of Effectiveness**. *Spine* 2004, **29**(10).
3. Adamczyk A, Kiebzak W, Wilk-Franczuk M, Sliwinski Z: **Effectiveness of holistic physiotherapy for low back pain**. *Ortop Traumatol Rehabil* 2009, **11**(6):562–76.
4. Alcoff J, Jones E, Rust P, Newman R: **Controlled trial of imipramine for chronic low back pain**. *J Fam Pract* 1982, **14**(5):841–6.
5. Ansari NN, Ebadi S, Talebian S, Naghdi S, Mazaheri H, Olyaei G, Jalaie S: **A randomized, single blind placebo controlled clinical trial on the effect of continuous ultrasound on low back pain**. *Electromyogr Clin Neurophysiol* 2006, **46**(6):329–336.
6. L A, B F, D A, et al: **Activity of tetrazepam in low back pain.** *Clinical Trials Journal* 1990, (27):258?67.
7. Arden NK, Price C, Reading I, Stubbing J, Hazelgrove J, Dunne C, Michel M, Rogers P, Cooper C: **A multicentre randomized controlled trial of epidural corticosteroid injections for sciatica: the WEST study**. *Rheumatology (Oxford)* 2005, **44**(11):1399–406.
8. Arkuszewski Z: **The efficacy of manual treatment in low-back pain: a clinical trial.** *Manual Medicine* 1986, **2**:68–71.
9. Arts MP, Peul WC, Brand R, Koes BW, Thomeer RT: **Cost-effectiveness of microendoscopic discectomy versus conventional open discectomy in the treatment of lumbar disc herniation: a prospective randomised controlled trial [ISRCTN51857546]**. *BMC Musculoskelet Disord* 2006, **7**:42.
10. Atkinson JH, Slater MA, Wahlgren DR, Williams RA, Zisook S, Pruitt SD, Epping-Jordan JE, Patterson TL, Grant I, Abramson I, Garfin SR: **Effects of noradrenergic and serotonergic antidepressants on chronic low back pain intensity**. *Pain* 1999, **83**(2):137–45.
11. Atkinson JH, Slater MA, Williams RA, Zisook S, Patterson TL, Grant I, Wahlgren DR, Abramson I, Garfin SR: **A placebo-controlled randomized clinical trial of nortriptyline for chronic low back pain**. *Pain* 1998, **76**(3):287–96.
12. Barker KL, Elliott CJ, Sackley CM, Fairbank JC: **Treatment of chronic back pain by sensory discrimination training. A Phase I RCT of a novel device (FairMed) vs. TENS**. *BMC Musculoskelet Disord* 2008, **9**:97.
13. Basler HD, Bertalanffy H, Quint S, Wilke A, Wolf U: **TTM-based counselling in physiotherapy does not contribute to an increase of adherence to activity recommendations in older adults with chronic low back pain–a randomised controlled trial**. *Eur J Pain* 2007, **11**:31–7.
14. Basmajian JV: **Acute back pain and spasm. A controlled multicenter trial of combined analgesic and antispasm agents**. *Spine* 1989, **14**(4):438–9.
15. Belavy DL, Armbrecht G, Gast U, Richardson CA, Hides JA, Felsenberg D: **Countermeasures against lumbar spine deconditioning in prolonged bed rest: resistive exercise with and without whole body vibration**. *J Appl Physiol* 2010, **109**(6):1801–11.
16. Bendix AE, Bendix T, Haestrup C, Busch E: **A prospective, randomized 5-year follow-up study of functional restoration in chronic low back pain patients**. *Eur.Spine J* 1998, **7**(2):111–119.
17. Bendix AF, Bendix T, Vaegter K, Lund C, Frolund L, Holm L: **Multidisciplinary intensive treatment for chronic low back pain: a randomized, prospective study**. *Cleve Clin J Med* 1996, **63**:62–9.
18. Bendix T, Bendix A, Labriola M, Haestrup C, Ebbehoj N: **Functional restoration versus outpatient physical training in chronic low back pain: a randomized comparative study**. *Spine* 2000, **25**(19):2494–2500.

19. Bergholdt K, Fabricius RN, Bendix T: **Better backs by better beds?** *Spine (Phila Pa 1976)* 2008, **33**(7):703–8.

20. Berry H, Hutchinson DR: **A multicentre placebo-controlled study in general practice to evaluate the efficacy and safety of tizanidine in acute low-back pain**. *J Int Med Res* 1988, **16**(2):75–82.

21. Blazek M, Keszthelyi B, Varhelyi M, Korosi O: **Comparative study of Biarison and Voltaren in acute lumbar pain and lumbo-ischialgia**. *Ther Hung* 1986, **34**(3):163–6.

22. Blomberg S, Svardsudd K, Tibblin G: **A randomized study of manual therapy with steroid injections in low-back pain. Telephone interview follow-up of pain, disability, recovery and drug consumption**. *Eur Spine J* 1994, **3**(5):246–54.

23. Bogefeldt J, Grunnesjo MI, Svardsudd K, Blomberg S: **Sick leave reductions from a comprehensive manual therapy programme for low back pain: the Gotland Low Back Pain Study**. *Clin Rehabil* 2008, **22**(6):529–41.

24. Bonetti M, Fontana A, Cotticelli B, Volta GD, Guindani M, Leonardi M: **Intraforaminal O(2)-O(3) versus periradicular steroidal infiltrations in lower back pain: randomized controlled study**. *AJNR Am J Neuroradiol* 2005, **26**(5):996–1000.

25. Borenstein DG, Lacks S, Wiesel SW: **Cyclobenzaprine and naproxen versus naproxen alone in the treatment of acute low back pain and muscle spasm.** *Clin Ther* 1990, **12**(2):125–131.

26. Brinkhaus B, Becker-Witt C, Jena S, Linde K, Streng A, Wagenpfeil S, Irnich D, Hummelsberger J, Melchart D, Willich SN: **Acupuncture Randomized Trials (ART) in patients with chronic low back pain and osteoarthritis of the knee - design and protocols**. *Forsch Komplementarmed Klass Naturheilkd* 2003, **10**(4):185–91.

27. Bronfort G: **Chiropractic versus general medical treatment of low back pain: a small scale controlled clinical trial.** *Am J Chin Med* 1989, **2**:145–50.

28. Bronfort G, Maiers MJ, Evans RL, Schulz CA, Bracha Y, Svendsen KH, Grimm Jr RH, Owens Jr EF, Garvey TA, Transfeldt EE: **Supervised exercise, spinal manipulation, and home exercise for chronic low back pain: A randomized clinical trial**. *Spine Journal* 2011, **11**(7):585–598.

29. Browder DA, Childs JD, Cleland JA, Fritz JM: **Effectiveness of an extension-oriented treatment approach in a subgroup of subjects with low back pain: a randomized clinical trial**. *Phys Ther* 2007, **87**(12):1608–18; discussion 1577–9.

30. Brown KC, Sirles AT, Hilyer JC, Thomas MJ: **Cost-effectiveness of a back school intervention for municipal employees**. *Spine* 1992, **17**(10):1224–8.

31. Bru RBwea E; Mykletun: **Effects of different psychological interventions on neck, shoulder and low back pain in female hospital staff.** *Psychol Health* 1994, **9**:371–82.

32. Buchbinder R: **Population based intervention to change back pain beliefs and disability: three part evaluation**. *Brit med J* 2001, **322**:1516–1520.

33. Buenaver LF, McGuire L, Haythornthwaite JA: **Cognitive-Behavioral Self-Help for Chronic Pain**. *Journal of Clinical Psychology* 2006, **62**(11):1389–1396.

34. Buhrman M, Faltenhag S, Strom L, Andersson G: **Controlled trial of Internet-based treatment with telephone support for chronic back pain**. *Pain* 2004, **111**(3):368–77.

35. Buttagat V, Eungpinichpong W, Chatchawan U, Kharmwan S: **The immediate effects of traditional Thai massage on heart rate variability and stress-related parameters in patients with back pain associated with myofascial trigger points**. *J Bodyw Mov Ther* 2011, :15–23.

36. R B, D S, A O, I VH, C R, A S, B L, C L, M E: **Erratum: Efficacy and safety of tapentadol extended release for the management of chronic low back pain: results of a prospective, randomized, double-blind, placebo- and active-controlled Phase III study (Expert Opin. Pharmacother. (2010) 11 (1787-1804))**. *Expert Opin. Pharmacother.* 2010, (16):2773.

37. Buynak R, Etropolski M, Lange B, Shapiro DY, Okamoto A, Steup A, Van Hove I: **Dose stability of tapentadol er for the relief of chronic low back pain: Results of a randomized, active- and placebo-controlled study**. *Arthritis and Rheumatism* 2009, **60**:1494.

38. Cabitza P, Randelli P: **Efficacy and safety of eperisone in patients with low back pain: a double blind randomized study**. *Eur Rev Med Pharmacol Sci* 2008, **12**(4):229–35.

39. Cambron JA, Gudavalli MR, Hedeker D, McGregor M, Jedlicka J, Keenum M, Ghanayem AJ, Patwardhan AG, Furner SE: **One-year follow-up of a randomized clinical trial comparing flexion distraction with an exercise program for chronic low-back pain**. *J Altern Complement Med* 2006, **12**(7):659–68.

40. Carette S, Marcoux S, Truchon R, Grondin C, Gagnon J, Allard Y, Latulippe M: **A controlled trial of corticosteroid injections into facet joints for chronic low back pain**. *N Engl J Med* 1991, **325**(14):1002–7.

41. Carlsson CP, Sjolund BH: **Acupuncture for chronic low back pain: a randomized placebo-controlled study with long-term follow-up**. *Clin J Pain* 2001, **17**(4):296–305.

42. Carson JW, Keefe FJ, Lynch TR, Carson KM, Goli V, Fras AM, Thorp SR: **Loving-kindness meditation for chronic low back pain: results from a pilot trial**. *J Holist Nurs* 2005, **23**(3):287–304.

43. Carter JM, Beam WC, McMahan SG, Barr ML, Brown LE: **The effects of stability ball training on spinal stability in sedentary individuals**. *J Strength Cond Res* 2006, **20**(2):429–35.

44. Casserley-Feeney S HODA: **The ACCESS trial – randomised controlled trial of public hospital-based versus private clinic-based physiotherapy for low back pain: clinical outcomes**. *Spine* 2007, **37**(2):72–73.

45. Celestini M, Marchese A, Serenelli A, Graziani G: **A randomized controlled trial on the efficacy of physical exercise in patients braced for instability of the lumbar spine**. *Eura Medicophys* 2005, **41**(3):223–31.

46. Cevik R, Bilici A, Gur A, Sarac AJ, Yildiz H, Nas K, Ceviz A, Bukte Y: **Effect of new traction technique of prone position on distraction of lumbar vertebrae and its relation with different application of heating therapy in low back pain**. *Journal of Back and Musculoskeletal Rehabilitation* 2007, **20**(2-3):71–77.

47. Chair SY, Taylor-Piliae RE, Lam G, Chan S: **Effect of positioning on back pain after coronary angiography**. *J Adv Nurs* 2003, **42**(5):470–8.

48. Christensen FB, Hansen ES, Eiskjaer SP, Hoy K, Helmig P, Neumann P, Niedermann B, Bunger CE: **Circumferential lumbar spinal fusion with Brantigan cage versus posterolateral fusion with titanium Cotrel-Dubousset instrumentation: a prospective, randomized clinical study of 146 patients**. *Spine* 2002, **27**(23):2674–83.

49. Christiansen S, Oettingen G, Dahme B, Klinger R: **A short goal-pursuit intervention to improve physical capacity: A randomized clinical trial in chronic back pain patients**. *Pain* 2010, **149**(3):444–452.

50. Clark D, Chu L: **Tolerance and opioid-induced hyperalgesia in clinical populations**. *European Journal of Pain Supplements* 2010, **4**:29.

51. Clauw DJ, Harris RE: **Is acupuncture more effective than sham acupuncture in relieving pain in patients with low back pain?** *Nat Clin Pract Rheumatol* 2006, **2**(7):362–3.

52. Cleland JA, Fritz JM, Childs JD, Kulig K: **Comparison of the effectiveness of three manual physical therapy techniques in a subgroup of patients with low back pain who satisfy a clinical prediction rule: study protocol of a randomized clinical trial [NCT00257998]**. *BMC Musculoskelet Disord* 2006, **7**:11.

53. Coan RM, Wong G, Ku SL, Chan YC, Wang L, Ozer FT, Coan PL: **The acupuncture treatment of low back pain: a randomized controlled study**. *Am J Chin Med* 1980, **8**(1-2):181–9.

54. Codding C, Levinsky D, Hale ME, Thomas JW, Lockhart E, Best A, Jain R: **Efficacy and safety evaluation of 12 weeks extended-release hydrocodone/acetaminophen treatment in patients with chronic low back pain (CLBP) by prior opioid use**. *Pain Medicine* 2009, **10**:260.

55. Cohen SP, Stojanovic MP, Crooks M, Kim P, Schmidt RK, Shields CH, Croll S, Hurley RW: **Lumbar zygapophysial (facet) joint radiofrequency denervation success as a function of pain relief during diagnostic medial branch blocks: a multicenter analysis**. *Spine Journal* 2008, **8**(3):498–504.

56. Cole C: **Does acupuncture or massage work in people with persistent back pain?** *The Journal of family practice.* 2001, **50**(9):799.

57. Corcoll J, Orfila J, Tobajas P, Alegre L: **Implementation of neuroreflexotherapy for subacute and chronic neck and back pain within the Spanish public health system: Audit results after one year**. *Health Policy* 2006, **79**(2-3):345–357.

58. Corey D, Koepfler L, Etlin D, Day H: **A limited functional restoration program for injured workers: A randomized trial.** *Journal of Occupational Rehabilitation.* 1996, **6**(4):239–250.

59. Costantino C, Marangio E, Coruzzi G: **Mesotherapy versus Systemic Therapy in the Treatment of Acute Low Back Pain: A Randomized Trial**. *Evid Based Complement Alternat Med* 2011, **2011**.

60. Cox JM: **Letter to the Editor: A randomized controlled trial comparing 2 types of spinal manipulation and minimal conservative medical care for adults 55 years and older with subacute or chronic low back pain**. *J Manipulative Physiol Ther* 2009, **32**(7):601.

61. Critchley DJ, Ratcliffe J, Noonan S, Jones RH, Hurley MV: **Effectiveness and cost-effectiveness of three types of physiotherapy used to reduce chronic low back pain disability: a pragmatic randomized trial with economic evaluation**. *Spine (Phila Pa 1976)* 2007, **32**(14):1474–81.

62. Curnow D, Cobbin D, Wyndham J, Boris Choy ST: **Altered motor control, posture and the Pilates method of exercise prescription**. *J Bodyw Mov Ther* 2009, **13**:104–111.

63. Dagenais S, Yelland MJ, Del Mar C, Schoene ML: **Prolotherapy injections for chronic low-back pain**. *Cochrane Database Syst Rev* 2007, (2):CD004059.

64. Day IJ, Kent CF, Burnham RS: **Can topical anesthetic reduce the pain associated with diagnostic blocks of the lumbosacral spine?** *Pain Med* 2008, **9**(6):675–9.

65. Demoulin: **Benefits of a Physical Training Program After Back School for Chronic Low Back Pain Patients**. *Journal of Musculoskeletal Pain* 2006, **14**(2):21–31.

66. Deshpande A, Furlan A, Mailis-Gagnon A, Atlas S, Turk D: **Opioids for chronic low-back pain**. *Cochrane Database Syst Rev* 2007, (3):CD004959.

67. Desmoulin GT, Yasin NI, Chen DW: **Initial results using Khan Kinetic Treatment(trademark) as a low back pain treatment option**. *Journal of Musculoskeletal Pain* 2007, **15**(3):91–102.

68. Dianne Liddle S, Gracey JH, David Baxter G: **Advice for the management of low back pain: A systematic review of randomised controlled trials**. *Manual Therapy* 2007, **12**(4):310–327.

69. Dickens C, Jayson M, Sutton C, Creed F: **The relationship between pain and depression in a trial using paroxetine in sufferers of chronic low back pain**. *Psychosomatics* 2000, **41**(6):490–9.

70. Donaldson DDMea S; Romney: **Randomized study of the application of single motor unit biofeedback training to chronic low back pain.** *J Occup Rehabil* 1994, **4**.

71. Eisenberg DM, Post DE, Davis RB, Connelly MT, Legedza AT, Hrbek AL, Prosser LA, Buring JE, Inui TS, Cherkin DC: **Addition of choice of complementary therapies to usual care for acute low back pain: a randomized controlled trial**. *Spine (Phila Pa 1976)* 2007, **32**(2):151–8.

72. Engers A, Jellema P, Wensing M, van der Windt DA, Grol R, van Tulder MW: **Individual patient education for low back pain**. *Cochrane Database Syst Rev* 2008, :CD004057.

73. Enthoven P, Skargren E, Oberg B: **Clinical course in patients seeking primary care for back or neck pain: a prospective 5-year follow-up of outcome and health care consumption with subgroup analysis**. *Spine.* 2004, **29**(21):2458–65.

74. Erhard RE, Delitto A, Cibulka MT: **Relative effectiveness of an extension program and a combined program of manipulation and flexion and extension exercises in patients with acute low back syndrome.** *Phys Ther* 1994, **74**(12):1093–1100.

75. Etropolski M, Rauschkolb-Loffler C, Shapiro D, Okamoto A, Lange C: **A randomized, double-blind, placebo- and active-controlled phase III study of tapentadol ER for chronic low back pain: Analysis of efficacy endpoint sensitivity**. *Journal of Pain* 2009, **10**(4):S51.

76. Etropolski MS, Okamoto A, Shapiro DY, Rauschkolb C: **Dose conversion between tapentadol immediate and extended release for low back pain**. *Pain Physician* 2010, **13**:61–70.

77. Evans Cea: **A randomized controlled trial of flexion exercises, education, and bed rest for patients with acute low back pain.** *Physiotherapy Canada* 1987, **39**:96–101.

78. Evans DD, Carter M, Panico R, Kimble L, Morlock JT, Spears MJ: **Characteristics and Predictors of Short-Term Outcomes in Individuals Self-selecting Yoga or Physical Therapy for Treatment of Chronic Low Back Pain**. *PM and R* 2010, **2**(11):1006–1015.

79. Evans DP, Burke MS, Newcombe RG: **Medicines of choice in low back pain**. *Curr Med Res Opin* 1980, **6**(8):540–7.

80. Faas Aea: **A randomized, placebo-controlled trial of exercise therapy in patients with acute low back pain.** *Spine* 1993, **18**:1388–1395.

81. Facci LM, Nowotny JP, Tormem F, Trevisani VF: **Effects of transcutaneous electrical nerve stimulation (TENS) and interferential currents (IFC) in patients with nonspecific chronic low back pain: randomized clinical trial**. *Sao Paulo Med J* 2011, **129**(4):206–16.

82. Fanello S: **Evaluation of a training program for the prevention of lower back pain among hospital employees**. *Nursing and health sciences* 2002, **4**.

83. Farrell JP, Twomey LT: **Acute low back pain. Comparison of two conservative treatment approaches.** *Med J Aust* 1982, **1**(4):160–164.

84. Flynn TW, Childs JD, Fritz JM: **The audible pop from high-velocity thrust manipulation and outcome in individuals with low back pain**. *J Manipulative Physiol Ther* 2006, **29**:40–5.

85. Foster L, Clapp L, Erickson M, Jabbari B: **Botulinum toxin A and chronic low back pain: a randomized, double-blind study.** *Neurology* 2001, **56**(10):1290–1293.

86. Friedrich M, Gittler G, Halberstadt Y, Cermak T, Heiller I: **Combined exercise and motivation program: effect on the compliance and level of disability of patients with chronic low back pain: a randomized controlled trial**. *Arch.Phys.Med Rehabil* 1998, **79**(5):475–487.

87. Fritz JM, Delitto A, Erhard RE: **Comparison of classification-based physical therapy with therapy based on clinical practice guidelines for patients with acute low back pain: a randomized clinical trial**. *Spine.* 2003, **28**(13):1363–71; discussion 1372.

88. Fritz JM, Lindsay W, Matheson JW, Brennan GP, Hunter SJ, Moffit SD, Swalberg A, Rodriquez B: **Is there a subgroup of patients with low back pain likely to benefit from mechanical traction? Results of a randomized clinical trial and subgrouping analysis**. *Spine (Phila Pa 1976)* 2007, **32**(26):E793–800.

89. Fritzell P, Hagg O, Jonsson D, Nordwall A: **Cost-effectiveness of lumbar fusion and nonsurgical treatment for chronic low back pain in the Swedish Lumbar Spine Study: a multicenter, randomized, controlled trial from the Swedish Lumbar Spine Study Group**. *Spine* 2004, **29**(4):421–34; discussion Z3.

90. Fritzell P, Hagg O, Wessberg P, Nordwall A: **Chronic low back pain and fusion: a comparison of three surgical techniques: a prospective multicenter randomized study from the Swedish lumbar spine study group**. *Spine* 2002, **27**(11):1131–41.

91. Frost H, Lamb SE, Klaber Moffett JA, Fairbank JC, Moser JS: **A fitness programme for patients with chronic low back pain: 2-year follow-up of a randomised controlled trial**. *Pain* 1998, **75**(2-3):273–279.

92. Galantino ML, Bzdewka TM, Eissler-Russo JL, Holbrook ML, Mogck EP, Geigle P, Farrar JT: **The impact of modified Hatha yoga on chronic low back pain: a pilot study**. *Altern Ther Health Med* 2004, **10**(2):56–9.

93. Gale GD, Rothbart PJ, Li Y: **Infrared therapy for chronic low back pain: a randomized, controlled trial**. *Pain Res Manag* 2006, **11**(3):193–6.

94. Gatti R, Faccendini S, Tettamanti A, Barbero M, Balestri A, Calori G: **Efficacy of trunk balance exercises for individuals with chronic low back pain: a randomized clinical trial**. *J Orthop Sports Phys Ther* 2011, **41**(8):542–52.

95. Gemignani G, Olivieri I, Ruju G, Pasero G: **Transcutaneous electrical nerve stimulation in ankylosing spondylitis: a double-blind study**. *Arthritis Rheum* 1991, **34**(6):788–9.

96. George SZ, Childs JD, Teyhen DS, Wu SS, Wright AC, Dugan JL, Robinson ME: **Brief psychosocial education, not core stabilization, reduced incidence of low back pain: results from the Prevention of Low Back Pain in the Military (POLM) cluster randomized trial**. *BMC Med* 2011, **9**:128.

97. George SZ, Teyhen DS, Wu SS, Wright AC, Dugan JL, Yang G, Robinson ME, Childs JD: **Psychosocial education improves low back pain beliefs: results from a cluster randomized clinical trial (NCT00373009) in a primary prevention setting**. *Eur Spine J* 2009, **18**(7):1050–8.

98. George SZ, Wittmer VT, Fillingim RB, Robinson ME: **Comparison of graded exercise and graded exposure clinical outcomes for patients with chronic low back pain**. *J Orthop Sports Phys Ther* 2010, **40**(11):694–704.

99. George SZ, Zeppieri J G, Cere AL, Cere MR, Borut MS, Hodges MJ, Reed DM, Valencia C, Robinson ME: **A randomized trial of behavioral physical therapy interventions for acute and sub-acute low back pain (NCT00373867)**. *Pain* 2008, **140**:145–57.

100. Gerner P, Kao G, Srinivasa V, Narang S, Wang GK: **Topical amitriptyline in healthy volunteers**. *Regional anesthesia and pain medicine.* 2003, **28**(4):289–93.

101. Gilbert FJ, Grant AM, Gillan MG, Vale L, Scott NW, Campbell MK, Wardlaw D, Knight D, McIntosh E, Porter RW: **Does early imaging influence management and improve outcome in patients with low back pain? A pragmatic randomised controlled trial**. *Health technology assessment (Winchester, England)* 2004, **8**(17):iii, 1–131.

102. Giles LG, Muller R: **Chronic spinal pain: a randomized clinical trial comparing medication, acupuncture, and spinal manipulation**. *Spine.* 2003, **28**(14):1490–502; discussion 1502–3.

103. Giles LG, Muller R: **Chronic spinal pain syndromes: a clinical pilot trial comparing acupuncture, a nonsteroidal anti-inflammatory drug, and spinal manipulation**. *J Manipulative Physiol Ther* 1999, **22**(6):376–81.

104. Glaser JA, Baltz MA, Nietert PJ, Bensen CV: **Electrical muscle stimulation as an adjunct to exercise therapy in the treatment of nonacute low back pain: A randomized trial**. *Journal of Pain* 2001, **2**(5):295–300.

105. Godfrey CM, Morgan PP, Schatzker J: **A randomized trial of manipulation for low-back pain in a medical setting**. *Spine* 1984, **9**(3):301–4.

106. Goodkin K, Gullion CM, Agras WS: **A randomized, double-blind, placebo-controlled trial of trazodone hydrochloride in chronic low back pain syndrome.** *J Clin Psychopharmacol* 1990, **10**(4):269–278.

107. Goossens ME, Rutten-Van Mölken MP, Kole-Snijders AM, Vlaeyen JW, Van Breukelen G, Leidl R: **Health economic assessment of behavioural rehabilitation in chronic low back pain: a randomised clinical trial.** *Health Econ* 1998, **7**:39–51.

108. Goossens ME, Vlaeyen JW, Hidding A, Kole-Snijders A, Evers SM: **Treatment expectancy affects the outcome of cognitive-behavioral interventions in chronic pain**. *Clin J Pain* 2005, **21**:18–26; discussion 69–72.

109. Gould EM, Jensen MP, Victor TW, Gammaitoni AR, White RE, Galer BS: **The pain quality response profile of oxymorphone extended release in the treatment of low back pain**. *Clin J Pain* 2009, **25**(2):116–22.

110. Grunnesjo MI, Bogefeldt JP, Svardsudd KF, Blomberg SI: **A randomized controlled clinical trial of stay-active care versus manual therapy in addition to stay-active care: functional variables and pain**. *J Manipulative Physiol Ther* 2004, **27**(7):431–41.

111. Haas M, Groupp E, Kraemer DF: **Dose-response for chiropractic care of chronic low back pain**. *The spine journal : official journal of the North American Spine Society.* 2004, **4**(5):574–83.

112. Hagg O, Fritzell P, Nordwall A: **The clinical importance of changes in outcome scores after treatment for chronic low back pain**. *Eur Spine J* 2003, **12**:12–20.

113. Hale M, Khan A, Kutch M, Li S: **Once-daily OROS hydromorphone ER compared with placebo in opioid-tolerant patients with chronic low back pain**. *Curr Med Res Opin* 2010, **26**(6):1505–18.

114. Hale ME, Ahdieh H, Ma T, Rauck R: **Efficacy and safety of OPANA ER (oxymorphone extended release) for relief of moderate to severe chronic low back pain in opioid-experienced patients: a 12-week, randomized, double-blind, placebo-controlled study**. *J Pain* 2007, **8**(2):175–84.

115. Hale ME, Dvergsten C, Gimbel J: **Efficacy and Safety of Oxymorphone Extended Release in Chronic Low Back Pain: Results of a Randomized, Double-Blind, Placebo- and Active- Controlled Phase III Study**. *Journal of Pain* 2005, **6**:21–28.

116. Hall T, Hardt S, Schafer A, Wallin L: **Mulligan bent leg raise technique–a preliminary randomized trial of immediate effects after a single intervention**. *Man Ther* 2006, **11**(2):130–5.

117. Hamza MA, Ghoname EA, White PF, Craig WF, Ahmed HE, Gajraj NM, Vakharia AS, Noe CE: **Effect of the duration of electrical stimulation on the analgesic response in patients with low back pain**. *Anesthesiology* 1999, **91**(6):1622–7.

118. Hancock: **letter** 2010.

119. Hasegawa TM, Baptista AS, De Souza MC, Yoshizumi AM, Natour J: **Acupuncture for acute non-specific low back pain: A randomized, controlled, placebo trial**. *Arthritis and Rheumatism* 2009, **60**:1497.

120. Helmhout PH, Harts CC, Staal JB, Candel MJ, de Bie RA: **Comparison of a high-intensity and a low-intensity lumbar extensor training program as minimal intervention treatment in low back pain: a randomized trial**. *European spine journal : official publication of the European Spine Society, the European Spinal Deformity Society, and the European Section of the Cervical Spine Research Society.* 2004, **13**(6):537–47.

121. Helmhout PH, Harts CC, Viechtbauer W, Staal JB, de Bie RA: **Isolated lumbar extensor strengthening versus regular physical therapy in an army working population with nonacute low back pain: a randomized controlled trial**. *Arch Phys Med Rehabil* 2008, **89**(9):1675–85.

122. Helmhout PH, Staal JB, Heymans MW, Harts CC, Hendriks EJ, de Bie RA: **Prognostic factors for perceived recovery or functional improvement in non-specific low back pain: secondary analyses of three randomized clinical trials**. *Eur Spine J* 2010, **19**(4):650–9.

123. Hemmila HM, Keinanen-Kiukaanniemi SM, Levoska S, Puska P: **Does folk medicine work? A randomized clinical trial on patients with prolonged back pain**. *Arch.Phys.Med Rehabil* 1997, **78**(6):571–577.

124. Henchoz Y, Pinget C, Wasserfallen JB, Paillex R, de Goumoens P, Norberg M, Kai-Lik So A: **Cost-utility analysis of a three-month exercise programme vs usual care following multidisciplinary rehabilitation for chronic low back pain**. *J Rehabil Med* 2010, **42**(9):846–52.

125. Herzog W, Conway PJ, Willcox BJ: **Effects of different treatment modalities on gait symmetry and clinical measures for sacroiliac joint patients**. *J Manipulative Physiol Ther* 1991, **14**(2):104–9.

126. Heymans MW, Anema JR, Vet HC, Mechelen W: **Does flexion-distraction help treat chronic low back pain?** *Nat Clin Pract Rheumatol* 2006, (7):360–1.

127. Hides JA, Stanton WR, Mendis MD, Gildea J, Sexton MJ: **Effect of Motor Control Training On Muscle Size and Football Games Missed From Injury**. *Med Sci Sports Exerc* 2011.

128. Hlobil H, Staal JB, Twisk J, Koke A, Ariens G, Smid T, van Mechelen W: **The effects of a graded activity intervention for low back pain in occupational health on sick leave, functional status and pain: 12-month results of a randomized controlled trial**. *J Occup Rehabil* 2005, **15**(4):569–80.

129. Hlobil H, Uegaki K, Staal JB, de Bruyne MC, Smid T, van Mechelen W: **Substantial sick-leave costs savings due to a graded activity intervention for workers with non-specific sub-acute low back pain**. *Eur Spine J* 2007, **16**(7):919–24.

130. Hollinghurst S, Sharp D, Ballard K, Barnett J, Beattie A, Evans M, Lewith G, Middleton K, Oxford F, Webley F, Little P: **Randomised controlled trial of Alexander technique lessons, exercise, and massage (ATEAM) for chronic and recurrent back pain: economic evaluation**. *BMJ* 2008, **337**:a2656.

131. Holm I: **Fusion surgery is slightly better than non-surgical treatment in patients with severe chronic non-specific low back pain**. *The Australian journal of physiotherapy.* 2002, **48**(2):133.

132. Hsieh CYea: **Functional outcomes of low back pain: comparison of four treatment groups in a randomized controlled trial.** *J Manipulative Physiol Ther* 1992, **15**.

133. Hubley-Kozey CL, Vezina MJ: **Muscle activation during exercises to improve trunk stability in men with low back pain**. *Arch Phys Med Rehabil* 2002, **83**(8):1100–8.

134. Hurley DA, Minder PM, McDonough SM, Walsh DM, Moore AP, Baxter DG: **Interferential therapy electrode placement technique in acute low back pain: a preliminary investigation**. *Arch.Phys.Med Rehabil* 2001, **82**(4):485–493.

135. Hurley DA, O'Donoghue G, Tully MA, Moffett JK, van Mechelen W, Daly L, Boreham CA, McDonough SM: **A walking programme and a supervised exercise class versus usual physiotherapy for chronic low back pain: a single-blinded randomised controlled trial. (The Supervised Walking In comparison to Fitness Training for Back Pain (SWIFT) Trial)**. *BMC Musculoskelet Disord* 2009, **10**:79.

136. Hurwitz EL, Morgenstern H, Chiao C: **Effects of Recreational Physical Activity and Back Exercises on Low Back Pain and Psychological Distress: Findings From the UCLA Low Back Pain Study**. *American Journal of Public Health* 2005, **95**(10):817–1824.

137. Hurwitz EL, Morgenstern H, Harber P, Kominsky GF, Belin TR, Yu F, Adams AH: **The effectiveness of physical modalities among patients with low back pain randomized to chiropractic care: Findings from the UCLA low back pain study**. *Journal of Manipulative & Physiological Therapeutics* 2002, **25**:10–20.

138. Hurwitz EL, Morgenstern H, Kominski GF, Yu F, Chiang LM: **A randomized trial of chiropractic and medical care for patients with low back pain: Eighteen-month follow-up outcomes from the UCLA low back pain study**. *Spine* 2006, **31**(6):611–621.

139. Hush J: **TENS of unknown value in the treatment of chronic low back pain**. *Aust J Physiother* 2006, **52**:64.

140. Ijzelenberg H, Meerding WJ, Burdorf A: **Effectiveness of a back pain prevention program: A cluster randomized controlled trial in an occupational setting**. *Spine* 2007, **32**(7):711–719.

141. Ikegami S, Kamimura M, Uchiyama S, Nakagawa H, Hashidate H, Takahara K, Takahashi J, Kato H: **ANTI-nociceptive effects of elcatonin injection for postmenopausal women with back pain: A randomized controlled trial**. *Osteoporosis International* 2010, **21**:S197–S198.

142. Inoue M, Hojo T, Nakajima M, Kitakoji H, Itoi M: **Comparison of the effectiveness of acupuncture treatment and local anaesthetic injection for low back pain: a randomised controlled clinical trial**. *Acupunct Med* 2009, **27**(4):174–7.

143. Itoh K, Katsumi Y, Hirota S, Kitakoji H: **Effects of trigger point acupuncture on chronic low back pain in elderly patients–a sham-controlled randomised trial**. *Acupunct Med* 2006, **24**:5–12.

144. Jamison RN, Raymond SA, Slawsby EA, Nedeljkovic SS, Katz NP: **Opioid therapy for chronic noncancer back pain. A randomized prospective study.** *Spine (Phila Pa 1976)* 1998, **23**(23):2591–2600.

145. Jans MP, Korte d EM, Heinrich J, Hildebrandt VH: **Intermittent follow-up treatment with Cesar exercise therapy in patients with subacute or chronic aspecific low back pain: results of a randomized, controlled trial with a 1.5-year follow-up**. *Ned Tijdschr Fysiother* 2006, (5):111–116.

146. Jensen IB, Bergstrom G, Ljungquist T, Bodin L: **A 3-year follow-up of a multidisciplinary rehabilitation programme for back and neck pain**. *Pain* 2005, **115**(3):273–83.

147. Kaapa EH, Frantsi K, Sarna S, Malmivaara A: **Multidisciplinary group rehabilitation versus individual physiotherapy for chronic nonspecific low back pain: a randomized trial**. *Spine* 2006, **31**(4):371–6.

148. Karjalainen K, Malmivaara A, Mutanen P, Roine R, Hurri H, Pohjolainen T: **Mini-intervention for subacute low back pain: two-year follow-up and modifiers of effectiveness.** *Spine (Phila Pa 1976)* 2004, **29**(10):1069–1076.

149. Karjalainen K, Malmivaara A, Pohjolainen T, Hurri H, Mutanen P, Rissanen P, Pahkajarvi H, Levon H, Karpoff H, Roine R: **Mini-intervention for subacute low back pain: a randomized controlled trial**. *Spine* 2003, **28**(6):533–40; discussion 540–1.

150. Kasis AG, Marshman LAG, Krishna M, Bhatia CK: **Significantly improved outcomes with a less invasive posterior lumbar interbody fusion incorporating total facetectomy**. *Spine* 2009, **34**(6):572–577.

151. Katz J, Pennella-Vaughan J, Hetzel RD, Kanazi GE, Dworkin RH: **A randomized, placebo-controlled trial of bupropion sustained release in chronic low back pain**. *J Pain* 2005, **6**(10):656–61.

152. Katz N, Borenstein D, Birbara C, Bramson C, Nemeth M, Smith M, Brown M: **Tanezumab, an Anti-Nerve Growth Factor (NGF) antibody, for the treatment of chronic low back pain (CLBP) - A randomized, controlled, double-blind, phase 2 trial**. *Journal of Pain* 2009, **10**(4):S42.

153. Katz N, Rauck R, Ahdieh H, Ma T, Van Der Hoop RG, Kerwin R, Podolsky G: **A 12-week, randomized, placebo-controlled trial assessing the safety and efficacy of oxymorphone extended release for opioid-naive patients with chronic low back pain**. *Current Medical Research and Opinion* 2007, **23**:117–128.

154. Katz N, Rodgers DB, Krupa D, Reicin A: **Onset of pain relief with rofecoxib in chronic low back pain: results of two four-week, randomized, placebo-controlled trials**. *Curr Med Res Opin* 2004, **20**(5):651–8.

155. Kavanagh S, Lange B, Ashworth J, Etropolski MS, McNeill M, Rauschkolb C: **Tapentadol extended release (ER) for chronic low back pain: Results of euroqol-5 dimension (EQ-5D) and short form-36 (SF-36) health status questionnaires**. *Value in Health* 2009, **12**(7):A376.

156. Keijsers JF, Groenman NH, Gerards FM, van Oudheusden E, Steenbakkers M: **A back school in The Netherlands: evaluating the results**. *Patient Educ Couns* 1989, **14**:31–44.

157. Kendrick D: **The role of radiography in primary care patients with low back pain of at least 6 weeks duration: a randomised (unblinded) controlled trial**. *Health Technology Assessment* 2001, **5**(30).

158. Kerry Sea: **Radiography for low back pain: a randomised controlled trial and observational study in primary care**. *British Journal of General Practice,* 2002, **52**.

159. Kettenmann B, Wille C, Lurie-Luke E, Walter D, Kobal G: **Impact of continuous low level heatwrap therapy in acute low back pain patients: subjective and objective measurements**. *Clin J Pain* 2007, **23**(8):663–8.

160. Kinalski WPD R; Kuwik: **The comparison of the results of manual therapy versus physiotherapy methods used in treatment of patients with low back pain syndromes**. *Journal of manual medicine* 1989, **4**.

161. Kleinbohl D, Gortelmeyer R, Bender HJ, Holzl R: **Amantadine sulfate reduces experimental sensitization and pain in chronic back pain patients**. *Anesth Analg* 2006, **102**(3):840–7.

162. Koes BW, Bouter LM, van Mameren H, Essers AH, Verstegen GM, Hofhuizen DM, Houben JP, Knipschild PG: **The effectiveness of manual therapy, physiotherapy, and treatment by the general practitioner for nonspecific back and neck complaints. A randomized clinical trial**. *Spine* 1992, **17**:28–35.

163. Koes BW, Bouter LM, van Mameren H, Essers AH, Verstegen GM, Hofhuizen DM, Houben JP, Knipschild PG: **A blinded randomized clinical trial of manual therapy and physiotherapy for chronic back and neck complaints: physical outcome measures**. *J Manipulative Physiol Ther* 1992, **15**:16–23.

164. Koes BW, Bouter LM, van Mameren H, Essers AH, Verstegen GM, Hofhuizen DM, Houben JP, Knipschild PG: **Randomised clinical trial of manipulative therapy and physiotherapy for persistent back and neck complaints: results of one year follow up**. *Bmj* 1992, **304**(6827):601–5.

165. Kominski GF, Heslin KC, Morgenstern H, Hurwitz EL, Harber PI: **Economic evaluation of four treatments for low-back pain: results from a randomized controlled trial**. *Medical care.* 2005, **43**(5):428–35.

166. Konrad K, Tatrai T, Hunka A, Vereckei E, Korondi I: **Controlled trial of balneotherapy in treatment of low back pain**. *Ann Rheum Dis* 1992, **51**(6):820–2.

167. Kovacs F, Abraira V, Santos S, Diaz E, Gestoso M, Muriel A, Gil del Real MT, Mufraggi N, Noguera J, Zamora J: **A comparison of two short education programs for improving low back pain-related disability in the elderly: a cluster randomized controlled trial**. *Spine (Phila Pa 1976)* 2007, **32**(10):1053–9.

168. Kovacs FM, Llobera J, Abraira V, Lazaro P, Pozo F, Kleinbaum D: **Effectiveness and cost-effectiveness analysis of neuroreflexotherapy for subacute and chronic low back pain in routine general practice: a cluster randomized, controlled trial**. *Spine* 2002, **27**(11):1149–59.

169. Kucera M, Barna M, Hork O, Kl J, Kucera A, Hladva M: **Topical symphytum herb concentrate cream against myalgia: a randomized controlled double-blind clinical study**. *Advances in therapy.* 2005, **22**(6):681–92.

170. Kullich W, Schwann H, Machreich K, Ausserwinkler M: **Additional outcome improvement in the rehabilitation of chronic low back pain after nuclear resonance therapy**. *Rheumatologia* 2006, **20**:7–12.

171. Kullich W, Schwann H, Walcher J, Machreich K: **The effect of MBST-NuclearResonanceTherapy with a complex 3-dimensional electromagnetic nuclear resonance field on patients with low back pain 23**. *Journal of Back and Musculoskeletal Rehabilitation* 2006, (2-3):79–87.

172. Lamb SE, Lall R, Hansen Z, Castelnuovo E, Withers EJ, Nichols V, Griffiths F, Potter R, Szczepura A, Underwood M: **A multicentred randomised controlled trial of a primary care-based cognitive behavioural programme for low back pain. The Back Skills Training (BeST) trial**. *Health Technol Assess* 2010, **14**(41):1–253, iii–iv.

173. Lang E, Liebig K, Kastner S, Neundorfer B, Heuschmann P: **Multidisciplinary rehabilitation versus usual care for chronic low back pain in the community: effects on quality of life**. *Spine J.* 2003, **3**(4):270–276.

174. Lee JW, Shin HI, Park SY, Lee GY, Kang HS: **Therapeutic trial of fluoroscopic interlaminar epidural steroid injection for axial low back pain: effectiveness and outcome predictors**. *AJNR Am J Neuroradiol* 2010, **31**(10):1817–23.

175. Lee PB, Kim YC, Lim YJ, Lee CJ, Choi SS, Park SH, Lee JG, Lee SC: **Efficacy of pulsed electromagnetic therapy for chronic lower back pain: a randomized, double-blind, placebo-controlled study**. *J Int Med Res* 2006, **34**(2):160–7.

176. Lee TJ: **Pharmacologic treatment for low back pain: one component of pain care**. *Phys Med Rehabil Clin N Am* 2010, **21**(4):793–800.

177. Lehmann TR, Russell DW, Spratt KF, Colby H, Liu YK, Fairchild ML, Christensen S: **Efficacy of electroacupuncture and TENS in the rehabilitation of chronic low back pain patients**. *Pain* 1986, **26**(3):277–90.

178. Leichtfried V, Kantner-Rumplmair W, Raggautz M, Bartenbach C, Aigner M, Winkler D, Jonas L, Gehmacher D, Schobersberger W: **Can bright light therapy ameliorate symptoms associated with low back pain (LBP)? A randomized controlled trial**. *Journal of Psychosomatic Research* 2010, **68**(6):642.

179. Lewis C, Khan A, Souvlis T, Sterling M: **A randomised controlled study examining the short-term effects of Strain-Counterstrain treatment on quantitative sensory measures at digitally tender points in the low back**. *Man Ther* 2010, **15**(6):536–41.

180. Li C, Ni J, Wang Z, Li M, Gasparic M, Terhaag B, Uberall MA: **Analgesic efficacy and tolerability of flupirtine vs. tramadol in patients with subacute low back pain: a double-blind multicentre trial***. *Curr Med Res Opin* 2008, **24**(12):3523–30.

181. Lierz P, Gustorff B, Markow G, Felleiter P: **Comparison between bupivacaine 0.125outpatients with chronic low back pain**. *Eur J Anaesthesiol* 2004, **21**:32–7.

182. Lilius G, Laasonen EM, Myllynen P, Harilainen A, Gronlund G: **Lumbar facet joint syndrome. A randomised clinical trial**. *J Bone Joint Surg Br* 1989, **71**(4):681–4.

183. Limke JC, Rainville J, Pena E, Childs L: **Randomized trial comparing the effects of one set vs two sets of resistance exercises for outpatients with chronic low back pain and leg pain**. *Eur J Phys Rehabil Med* 2008, **44**(4):399–405.

184. Lin ML, Lin MH, Fen JJ, Lin WT, Lin CW, Chen PQ: **A comparison between pulsed radiofrequency and electro-acupuncture for relieving pain in patients with chronic low back pain**. *Acupunct Electrother Res* 2010, **35**(3-4):133–46.

185. Lindstrom I, Ohlund C, Eek C, Wallin L, Peterson LE, Nachemson A: **Mobility, strength, and fitness after a graded activity program for patients with subacute low back pain. A randomized prospective clinical study with a behavioral therapy approach**. *Spine* 1992, **17**(6):641–652.

186. Linton SJ, Nordin E: **A 5-year follow-up evaluation of the health and economic consequences of an early cognitive behavioral intervention for back pain: a randomized, controlled trial**. *Spine* 2006, **31**(8):853–8.

187. Loeser JD: **Prolotherapy Injections, Saline Injections, and Exercises for Chronic Low-Back Pain: A Randomized Trial - Point of View**. *Spine* 2004, **29**:16.

188. Loisel P, Abenhaim L, Durand P, Esdaile JM, Suissa S, Gosselin L, Simard R, Turcotte J, Lemaire J: **A population-based, randomized clinical trial on back pain management**. *Spine* 1997, **22**(24):2911–8.

189. Loisel P, Vachon B, Lemaire J, Durand MJ, Poitras S, Stock S, Tremblay C: **Discriminative and predictive validity assessment of the quebec task force classification**. *Spine.* 2002, **27**(8):851–7.

190. Long A, May S, Fung T: **The comparative prognostic value of directional preference and centralization: A useful tool for front-line clinicians?** *Journal of Manual and Manipulative Therapy* 2008, **16**(4):248–254.

191. Macdonald AJ, Macrae KD, Master BR, Rubin AP: **Superficial acupuncture in the relief of chronic low back pain.** *Ann R Coll Surg Engl* 1983, **65**:44–46.

192. Macfarlane GJ: **Changing patient perceptions of their illness: Can they contribute to an improved outcome for episodes of musculoskeletal pain?** *Pain* 2008, **136**(1-2):1–2.

193. Machado LA, Maher CG, Herbert RD, Clare H, McAuley J: **The McKenzie Method for the management of acute non-specific low back pain: design of a randomised controlled trial [ACTRN012605000032651]**. *BMC Musculoskelet Disord* 2005, **6**:50.

194. Magnussen L, Rognsvag T, Tveito TH, Eriksen HR: **Effect of a brief cognitive training programme in patients with long-lasting back pain evaluated as unfit for surgery**. *J Health Psychol* 2005, **10**(2):233–43.

195. Magnusson ML, Chow DH, Diamandopoulos Z, Pope MH: **Motor control learning in chronic low back pain**. *Spine (Phila Pa 1976)* 2008, **33**(16):E532–8.

196. Malmivaara A, Hakkinen U, Aro T, Heinrichs ML, Koskenniemi L, Kuosma E, Lappi S, Paloheimo R, Servo C, Vaaranen V: **The treatment of acute low back pain–bed rest, exercises, or ordinary activity?** *N.Engl.J.Med* 1995, **332**(6):351–355.

197. Mandara A, Fusaro A, Musicco M, Bado F: **A randomised controlled trial on the effectiveness of osteopathic manipulative treatment of chronic low back pain**. *International Journal of Osteopathic Medicine* 2008, **11**(4):156.

198. Manniche C, Lundberg E, Christensen I, Bentzen L, Hesselsoe G: **Intensive dynamic back exercises for chronic low back pain: a clinical trial**. *Pain* 1991, **47**:53–63.

199. Mannion AF, Taimela S, Muntener M, Dvorak J: **Active therapy for chronic low back pain part 1. Effects on back muscle activation, fatigability, and strength**. *Spine* 2001, **26**(8):897–908.

200. Matsumo S: **Clinical evaluation of ketoprofen (Orudis) in lumbage: a double blind comparison with diclofenac sodium**. *Br j clin practice* 1991, **35**:266.

201. Mattila R, Malmivaara A, Kastarinen M, Kivela SL, Nissinen A: **The effects of lifestyle intervention for hypertension on low back pain: a randomized controlled trial**. *Spine (Phila Pa 1976)* 2007, **32**(26):2943–7.

202. Mayer JM, Mooney V, Matheson LN, Erasala GN, Verna JL, Udermann BE, Leggett S: **Continuous low-level heat wrap therapy for the prevention and early phase treatment of delayed-onset muscle soreness of the low back: a randomized controlled trial**. *Arch Phys Med Rehabil* 2006, **87**(10):1310–7.

203. McIlveen B, Robertson VJ: **A Randomised Controlled Study of the Outcome of Hydrotherapy for Subjects with Low Back or Back and Leg Pain**. *Physiotherapy* 1998, **84**:17–26.

204. Meade TWea: **Randomised comparison of chiropractic and hospital outpatient management for low back pain: results from extended follow up**. *BMJ* 1995, **311**.

205. Mehling WE: **Breath therapy for chronic low back pain**. *Altern Ther Health Med* 2006, **11**(2):96–8.

206. Mehling WE, Hamel KA, Acree M, Byl N, Hecht FM: **Randomized, controlled trial of breath therapy for patients with chronic low-back pain**. *Altern Ther Health Med* 2005, **11**(4):44–52.

207. Mehta S, Chopra A, Goregaonkar A, Chandanwale A, Medhi B, Shah V, Langade D, Maroli S, Gaikwad S, Pawar D: **Evaluation of efficacy and safety of eperisone hydrochloride in treatment of acute musculoskeletal spasm associated with low back pain: A randomized, doubleblind, placebo-controlled trial**. *Pain Practice* 2009, **9**:123.

208. Mellin G, Hurri H, Härkäpää K, Järvikoski A: **A controlled study on the outcome of inpatient and outpatient treatment of low back pain. Part II. Effects on physical measurements three months after treatment.** *Scand J Rehabil Med* 1989, **21**(2):91–95.

209. Mellin G, Hurri H, Harkapaa K, Jarvikoski A: **A controlled study on the outcome of inpatient and outpatient treatment of low back pain. Part II. Effects on physical measurements three months after treatment**. *Scand J Rehabil Med* 1989, **21**(2):91–5.

210. Meng CF, Wang D, Ngeow J, Lao L, Peterson M, Paget S: **Acupuncture for chronic low back pain in older patients: a randomized, controlled trial**. *Rheumatology (Oxford, England)* 2003, **42**(12):1508–17.

211. Menzel NN, Lilley S, Robinson ME: **Interventions to reduce back pain in rehabilitation hospital nursing staff**. *Rehabil Nurs* 2006, **31**(4):138–47; discussion 148.

212. Menzel NN, Robinson ME: **Back pain in direct patient care providers: early intervention with cognitive behavioral therapy**. *Pain Manag Nurs* 2006, **7**(2):53–63.

213. Milgrom C, Finestone A, Lubovsky O, Zin D, Lahad A: **A controlled randomized study of the effect of training with orthoses on the incidence of weight bearing induced back pain among infantry recruits**. *Spine.* 2005, **30**(3):272–5.

214. Mirovsky Y, Grober A, Blankstein A, Stabholz L: **The effect of ambulatory lumbar traction combined with treadmill on patients with chronic low back pain**. *Journal of Back and Musculoskeletal Rehabilitation* 2006, **19**(2-3):73–78.

215. Miyazaki S, Hagihara A, Kanda R, Mukaino Y, Nobutomo K: **Applicability of press needles to a double-blind trial: a randomized, double-blind, placebo-controlled trial**. *Clin J Pain* 2009, **25**(5):438–44.

216. Modic MT, Obuchowski NA, Ross JS, Brant-Zawadzki MN, Grooff PN, Mazanec DJ, Benzel EC: **Acute low back pain and radiculopathy: MR imaging findings and their prognostic role and effect on outcome**. *Radiology* 2005, **237**(2):597–604.

217. Moffett JK, Jackson DA, Gardiner ED, Torgerson DJ, Coulton S, Eaton S, Mooney MP, Pickering C, Green AJ, Walker LG, May S, Young S: **Randomized trial of two physiotherapy interventions for primary care neck and back pain patients: 'McKenzie' vs brief physiotherapy pain management**. *Rheumatology* 2006, **45**(12):1514–1521.

218. Mooney V: **Manual therapy and exercise therapy in patients with chronic low back pain: a randomized, controlled trial with 1-year follow-up**. *Spine* 2004, **29**:107; author reply 107–8.

219. Muller R, Giles LG: **Long-term follow-up of a randomized clinical trial assessing the efficacy of medication, acupuncture, and spinal manipulation for chronic mechanical spinal pain syndromes**. *J Manipulative Physiol Ther* 2005, **28**:3–11.

220. Mullican WS, Lacy JR: **Tramadol/acetaminophen combination tablets and codeine/acetaminophen combination capsules for the management of chronic pain: a comparative trial**. *Clin Ther* 2001, **23**(9):1429–45.

221. Murtezani A, Hundozi H, Orovcanec N, Sllamniku S, Osmani T: **A comparison of high intensity aerobic exercise and passive modalities for the treatment of workers with chronic low back pain: a randomized, controlled trial**. *Eur J Phys Rehabil Med* 2011, **47**(3):359–66.

222. Najm WI: **German acupuncture trials (GERAC) for chronic low back pain**. *Medical Acupuncture* 2008, **20**(2):131–132.

223. Nath S, Nath CA, Pettersson K: **Percutaneous lumbar zygapophysial (Facet) joint neurotomy using radiofrequency current, in the management of chronic low back pain: a randomized double-blind trial**. *Spine (Phila Pa 1976)* 2008, **33**(12):1291–7; discussion 1298.

224. Natour J, Puertas EB, Radu AS, Freire M, Bonfiglioli R, Schincariol NRB, Da Mota Falca o D, Prado F: **Loxoprofen in the treatment of low back pain - Clinical efficacy and safety in comparison to diclofenac**. *Revista Brasileira de Medicina* 2002, **59**(3):161–170.

225. Nelson-Wong E, Callaghan JP: **Changes in muscle activation patterns and subjective low back pain ratings during prolonged standing in response to an exercise intervention**. *J Electromyogr Kinesiol.* 2010, **20**(6):1125–33.

226. Ney JP, Difazio M, Sichani A, Monacci W, Foster L, Jabbari B: **Treatment of Chronic Low Back Pain With Successive Injections of Botulinum Toxin A Over 6 Months: A Prospective Trial of 60 Patients**. *Clinical Journal of Pain* 2006, **22**(4):363–369.

227. North RB, Kidd DH, Olin J, Sieracki JM, Boulay M: **Spinal cord stimulation with interleaved pulses: A randomized, controlled trial**. *Neuromodulation* 2007, **10**(4):349–357.

228. Nouwen A, Cloutier C, Kappas A, Warbrick T, Sheffield D: **Effects of focusing and distraction on cold pressor-induced pain in chronic back pain patients and control subjects**. *J Pain* 2006, **7**:62–71.

229. Nuhr M, Hoerauf K, Bertalanffy A, Bertalanffy P, Frickey N, Gore C, Gustorff B, Kober A: **Active warming during emergency transport relieves acute low back pain**. *Spine.* 2004, **29**(14):1499–503.

230. O'Brien N, Hanlon M, Meldrum D: **Randomised, controlled trial comparing physiotherapy and Pilates in the treatment of ordinary low back pain**. *Physical Therapy Reviews* 2006, **11**(3):224–225.

231. O'Donnell JB, Ekman EF, Spalding WM, Bhadra P, McCabe D, Berger MF: **The effectiveness of a weak opioid medication versus a cyclo-oxygenase-2 (COX-2) selective non-steroidal anti-inflammatory drug in treating flare-up of chronic low-back pain: results from two randomized, double-blind, 6-week studies**. *J Int Med Res* 2009, **37**(6):1789–802.

232. Paatelma M, Kilpikoski S, Simonen R, Heinonen A, Alen M, Videman T: **Orthopaedic manual therapy, McKenzie method or advice only for low back pain in working adults: a randomized controlled trial with one year follow-up**. *J Rehabil Med* 2008, **40**(10):858–63.

233. Padua R, Bondi R, Ceccarelli E, Alviti F: **Re: A randomized study of back school in women with chronic low back pain. Quality of life at three, six, and twelve months follow-up**. *Spine (Phila Pa 1976)* 2009, **34**(12):1336.

234. Pal B, Mangion P, Hossain MA, Diffey BL: **A controlled trial of continuous lumbar traction in the treatment of back pain and sciatica**. *Br.J Rheumatol.* 1986, **25**(2):181–183.

235. Pareek A, Chandurkar N, Chandanwale AS, Ambade R, Gupta A, Bartakke G: **Aceclofenac-tizanidine in the treatment of acute low back pain: a double-blind, double-dummy, randomized, multicentric, comparative study against aceclofenac alone**. *Eur Spine J* 2009, **18**(12):1836–42.

236. Pengel LH, Refshauge KM, Maher CG, Nicholas MK, Herbert RD, McNair P: **Physiotherapist-directed exercise, advice, or both for subacute low back pain: a randomized trial**. *Ann Intern Med* 2007, **146**(11):787–96.

237. Peniston JH, Gould E: **Oxymorphone extended release for the treatment of chronic low back pain: a retrospective pooled analysis of enriched-enrollment clinical trial data stratified according to age, sex, and prior opioid use**. *Clin Ther* 2009, **31**(2):347–59.

238. Perrot S, Krause D, Crozes P, Naim C: **Efficacy and tolerability of paracetamol/tramadol (325 mg/37.5 mg) combination treatment compared with tramadol (50 mg) monotherapy in patients with subacute low back pain: a multicenter, randomized, double-blind, parallel-group, 10-day treatment study**. *Clin Ther* 2006, **28**(10):1592–606.

239. Petersen T, Larsen K, Jacobsen S: **One-year follow-up comparison of the effectiveness of McKenzie treatment and strengthening training for patients with chronic low back pain: outcome and prognostic factors**. *Spine (Phila Pa 1976)* 2007, **32**(26):2948–56.

240. Petersen T, Larsen K, Nordsteen J, Olsen S, Fournier G, Jacobsen S: **The McKenzie method compared with manipulation when used adjunctive to information and advice in low back pain patients presenting with centralization or peripheralization: a randomized controlled trial**. *Spine (Phila Pa 1976)* 2011, **36**(24):1999–2010.

241. Petrofsky JS, Batt J, Brown J, Stacey L, Bartelink T, Le Moine M, Charbonnet M, Leyva S, Lohman EB, Aiyar S, Christensen A, Weis D, Weis M, Jackson J, Rad-Bayani E, Prowse M, Sharma A, Rendon A: **Improving the outcomes after back injury by a core muscle strengthening program**. *The Journal of Applied Research* 2008, **8**:62–75.

242. Pneumaticos SG, Chatziioannou SN, Hipp JA, Moore WH, Esses SI: **Low back pain: prediction of short-term outcome of facet joint injection with bone scintigraphy**. *Radiology* 2006, **238**(2):693–8.

243. Podichetty VK, Varley ES: **Re: Oleske D M, Lavender S A, Andersson G B, et al. Are back supports plus education more effective than education alone in promoting recovery from low back pain? Results from a randomized clinical trial. Spine 2007;32:2050-7**. *Spine (Phila Pa 1976)* 2008, **33**(3):349–50.

244. Popovic DB, Bijelic G, Miler V, Dosen S, Popovic MB, Schwirtlich L: **Lumbar stimulation belt for therapy of low-back pain**. *Artif Organs* 2009, **33**:54–60.

245. Portenoy RK, Messina J, Xie F, Peppin J: **Fentanyl buccal tablet (FBT) for relief of breakthrough pain in opioid-treated patients with chronic low back pain: a randomized, placebo-controlled study**. *Curr Med Res Opin* 2007, **23**:223–33.

246. Postacchini MPP F; Facchini: **Efficacy of various forms of conservative treatment in low back pain: a comparative study.** *Neuro-Orthopedics* 1988, **6**.

247. del Pozo-Cruz B, Hernandez Mocholi MA, Adsuar JC, Parraca JA, Muro I, Gusi N: **Effects of whole body vibration therapy on main outcome measures for chronic non-specific low back pain: a single-blind randomized controlled trial**. *J Rehabil Med* 2011, **43**(8):689–94.

248. Quartana PJ, Burns JW, Lofland KR: **Attentional strategy moderates effects of pain catastrophizing on symptom-specific physiological responses in chronic low back pain patients**. *J Behav Med* 2007, **30**(3):221–31.

249. Ralph L, Look M, Wheeler W, Sacks H: **Double-blind, placebo-controlled trial of carisoprodol 250-mg tablets in the treatment of acute lower-back spasm**. *Curr Med Res Opin* 2008, **24**(2):551–8.

250. Ralph L, Wheeler B, Sacks H: **Improvement in functional status with carisoprodol 250-mg tablets in patients with acute lower back spasm: A randomized, double-blind, placebo-controlled trial**. *Pain Medicine* 2009, **10**:258.

251. Rashiq S, Koller M, Haykowsky M, Jamieson K: **The effect of opioid analgesia on exercise test performance in chronic low back pain**. *Pain* 2003, **106**(1-2):119–25.

252. Rasmussen-Barr E, Nilsson-Wikmar L, Arvidsson I: **Stabilizing training compared with manual treatment in sub-acute and chronic low-back pain.** *Man Ther* 2003, **8**(4):233–241.

253. Ratcliffe J, Thomas KJ, MacPherson H, Brazier J: **A randomised controlled trial of acupuncture care for persistent low back pain: Cost effectiveness analysis**. *BMJ: British Medical Journal* 2006, **333**(7569):1–5.

254. Rauck RL, Bookbinder SA, Bunker TR, Alftine CD, Ghalie R, Negro-Vilar A, de Jong E, Gershon S: **A randomized, open-label study of once-a-day AVINZA (morphine sulfate extended-release capsules) versus twice-a-day OxyContin (oxycodone hydrochloride controlled-release tablets) for chronic low back pain: the extension phase of the ACTION trial**. *J Opioid Manag* 2006, **2**(6):325–8, 331–3.

255. Rauck RL, Bookbinder SA, Bunker TR, Alftine CD, Ghalie R, Negro-Vilar A, de Jong E, Gershon S: **The ACTION study: a randomized, open-label, multicenter trial comparing once-a-day extended-release morphine sulfate capsules (AVINZA) to twice-a-day controlled-release oxycodone hydrochloride tablets (OxyContin) for the treatment of chronic, moderate to severe low back pain**. *J Opioid Manag* 2006, **2**(3):155–66.

256. Seroussi R, Gliner B, Steinitz E, Schmitt S, Gamburd R, Firlik A: **Effectiveness of percutaneous neuromodulation therapy for patients with chronic and severe low back pain**. *Pain practice* 2003, **3**:22–30.

257. Risch SV, Norvell NK, Pollock ML, Risch ED, Langer H, Fulton M, Graves JE, Leggett SH: **Lumbar strengthening in chronic low back pain patients. Physiologic and psychological benefits**. *Spine* 1993, **18**(2):232–238.

258. Rivero Arias O, Campbell H, Gray A, Fairbank J, Frost H, Wilson MacDonald J: **Surgical stabilisation of the spine compared with a programme of intensive rehabilitation for the management of patients with chronic low back pain: cost utility analysis based on a randomised controlled trial**. *BMJ (Clinical research ed.)* 2005, **330**(7502):1239.

259. Rivero Arias O, Gray A, Frost H, Lamb SE, Stewart Brown S: **Cost-utility analysis of physiotherapy treatment compared with physiotherapy advice in low back pain**. *Spine.* 2006, **31**(12):1381–7.

260. Romano CL, Romano D, Bonora C, Mineo G: **Pregabalin, celecoxib, and their combination for treatment of chronic low-back pain**. *J Orthop Traumatol* 2009, **10**(4):185–91.

261. Rusinyol FC, Perice RV, Boronat ER, Bosch FF: **Effects of two different doses of eperisone in the treatment of acute low back pain**. *Journal of Applied Research* 2009, **9**(1-2):23–29.

262. Ruth M, Weber M, Zenz M: **Laser acupuncture for chronic back pain. A double-blind clinical study**. *Schmerz* 2010, **24**(5):485–493.

263. Rydeard R, Leger A, Smith D: **Pilates-based therapeutic exercise: effect on subjects with nonspecific chronic low back pain and functional disability: a randomized controlled trial**. *J Orthop Sports Phys Ther* 2006, **36**(7):472–84.

264. Sasso RC, Kitchel SH, Dawson EG: **A prospective, randomized controlled clinical trial of anterior lumbar interbody fusion using a titanium cylindrical threaded fusion device**. *Spine* 2004, **29**(2):113–22; discussion 121–2.

265. Scheel IB, Hagen KB, Herrin J, Carling C, Oxman AD: **Blind faith? The effects of promoting active sick leave for back pain patients: a cluster-randomized controlled trial**. *Spine.* 2002, **27**(23):2734–40.

266. Schimmel JJ, de Kleuver M, Horsting PP, Spruit M, Jacobs WC, van Limbeek J: **No effect of traction in patients with low back pain: a single centre, single blind, randomized controlled trial of Intervertebral Differential Dynamics Therapy**. *Eur Spine J* 2009, **18**(12):1843–50.

267. Schmidt-Wilcke T: **Affective components and intensity of pain correlate with structural differences in gray matter in chronic back pain patients**. *Pain* 2006, **125**(1-2):89–97.

268. Schwarz I, Lawrence DJ: **Relative Responsiveness of 3 Different Types of Clinical Outcome Measures on Chiropractic Patients with Low Back Pain**. *Journal of Manipulative and Physiological Therapeutics* 2007, **30**:77–78.

269. Serfer GT, Wheeler WJ, Sacks HJ: **Randomized, double-blind trial of carisoprodol 250 mg compared with placebo and carisoprodol 350 mg for the treatment of low back spasm**. *Curr Med Res Opin* 2010, **26**:91–9.

270. Serferlis Tea: **Conservative treatment in patients sick-listed for acute low-back pain: a prospective randomised study with 12 months? follow-up**. *Eur Spine J* 1998, **7**.

271. Sertpoyraz F, Eyigor S, Karapolat H, Capaci K, Kirazli Y: **Comparison of isokinetic exercise versus standard exercise training in patients with chronic low back pain: a randomized controlled study**. *Clin Rehabil* 2009, **23**(3):238–47.

272. Shakoor MA, Salek AKM, Islam MT, Moyeenuzzaman M: **Evaluation of the effects of selective rehabilitation on the patients with chronic low back pain**. *International Journal of Rheumatic Diseases* 2010, **13**:221.

273. Shaughnessy M, Caulfield B: **A pilot study to investigate the effect of lumbar stabilisation exercise training on functional ability and quality of life in patients with chronic low back pain**. *Int J Rehabil Res* 2004, **27**(4):297–301.

274. Sherman KJ, Cherkin DC, Ichikawa L, Avins AL, Barlow WE, Khalsa PS, Deyo RA: **Characteristics of patients with chronic back pain who benefit from acupuncture**. *BMC Musculoskelet Disord* 2009, **10**:114.

275. Sherman KJ, Cherkin DC, Ichikawa L, Avins AL, Delaney K, Barlow WE, Khalsa PS, Deyo RA: **Treatment expectations and preferences as predictors of outcome of acupuncture for chronic back pain**. *Spine (Phila Pa 1976)* 2010, **35**(15):1471–7.

276. Sherry E, Kitchener P, Smart R: **A prospective randomized controlled study of VAX-D and TENS for the treatment of chronic low back pain**. *Neurol Res* 2001, **23**(7):780–4.

277. Shimoji K, Takahashi N, Nishio Y, Koyanagi M, Aida S: **Pain relief by transcutaneous electric nerve stimulation with bidirectional modulated sine waves in patients with chronic back pain: a randomized, double-blind, sham-controlled study**. *Neuromodulation* 2007, **10**:42–51.

278. Shum G: **Movement coordination of the lumbar spine and hip during a picking up activity in low back pain subjects**. *Eur Spine J.* 2006, **16**(6):749–58.

279. Skargren EI, Carlsson PG, Oberg BE: **One-year follow-up comparison of the cost and effectiveness of chiropractic and physiotherapy as primary management for back pain. Subgroup analysis, recurrence, and additional health care utilization.** *Spine (Phila Pa 1976)* 1998, **23**(17):1875–83; discussion 1884.

280. Skargren EI, Oberg BE: **Predictive factors for 1-year outcome of low-back and neck pain in patients treated in primary care: comparison between the treatment strategies chiropractic and physiotherapy.** *Pain* 1998, **77**(2):201–207.

281. Skargren EI, Oberg BE, Carlsson PG, Gade M: **Cost and effectiveness analysis of chiropractic and physiotherapy treatment for low back and neck pain. Six-month follow-up.** *Spine (Phila Pa 1976)* 1997, **22**(18):2167–2177.

282. Skljarevski V, Zhang S, Desaiah D, Palacios S, Miazgowski T, Patrickm K: **Effect of duloxetine 60 mg once daily versus placebo in patients with chronic low back pain: A 12-week, randomized, double-blind trial**. *Pain Medicine* 2010, **11**(2):322.

283. Skouen JS, Grasdal AL, Haldorsen EM, Ursin H: **Relative cost-effectiveness of extensive and light multidisciplinary treatment programs versus treatment as usual for patients with chronic low back pain on long-term sick leave: randomized controlled study**. *Spine.* 2002, **27**(9):901–9; discussion 909–10.

284. Slater MA, Weickgenant AL, Greenberg MA, Wahlgren DR, Williams RA, Carter C, Patterson TL, Grant I, Garfin SR, Webster JS, Atkinson JH: **Preventing progression to chronicity in first onset, subacute low back pain: an exploratory study**. *Arch Phys Med Rehabil* 2009, **90**(4):545–52.

285. Smeets RJ: **Do lumbar stabilising exercises reduce pain and disability in patients with recurrent low back pain?** *Aust J Physiother* 2009, **55**(2):138.

286. Smeets RJ, Vlaeyen JW, Hidding A, Kester AD, van der Heijden GJ, Knottnerus JA: **Chronic low back pain: physical training, graded activity with problem solving training, or both? The one-year post-treatment results of a randomized controlled trial**. *Pain* 2008, **134**(3):263–76.

287. Smeets RJEM, Beelen S, Goossens MEJB, Schouten EGW, Knottnerus JA, Vlaeyen JWS: **Treatment expectancy and credibility are associated with the outcome of both physical and cognitive-behavioral treatment in chronic low back pain**. *Clinical Journal of Pain* 2008, **24**(4):305–315.

288. Smeets RJEM, Vlaeyen JWS, Hidding A, Kester ADM, Van Der Heijden GJMG, Van Geel ACM, Knottnerus JA: **Active rehabilitation for chronic low back pain: Cognitive-behavioral, physical, or both? First direct post-treatment results from a randomized controlled trial [ISRCTN22714229]**. *BMC Musculoskelet Disord* 2006, **7**.

289. Smeets RJEM, Vlaeyen JWS, Kester ADM, Knottnerus JA: **Reduction of Pain Catastrophizing Mediates the Outcome of Both Physical and Cognitive-Behavioral Treatment in Chronic Low Back Pain**. *Journal of Pain* 2006, **7**(4):261–271.

290. Smith AL, Kolt GS, McConville JC: *The effect of the Felenkrais method on pain and anxiety in people experiencing chronic low back pain.* 1, NewZealand: New Zealand Society of Physiotherapists (Inc.) 2007.

291. Snook SH, Webster BS, McGorry RW: **The reduction of chronic, nonspecific low back pain through the control of early morning lumbar flexion: 3-year follow-up**. *J Occup.Rehabil* 2002, **12**:13–19.

292. Soonawalla DF, Joshi N: **Efficacy of thiocolchicoside in Indian patients suffering from low back pain associated with muscle spasm**. *J Indian Med Assoc* 2008, **106**(5):331–5.

293. Spratt KF, Weinstein JN, Lehmann TR, Woody J, Sayre H: **Efficacy of flexion and extension treatments incorporating braces for low-back pain patients with retrodisplacement, spondylolisthesis, or normal sagittal translation**. *Spine* 1993, **18**(13):1839–1849.

294. Standaert CJ: **Bed rest or continuation of activity for acute low back pain?** *Clinical journal of sport medicine : official journal of the Canadian Academy of Sport Medicine.* 2003, **13**(4):275.

295. Steenstra IA, Anema JR, Bongers PM, de Vet HC, Knol DL, van Mechelen W: **The effectiveness of graded activity for low back pain in occupational healthcare**. *Occup Environ Med* 2006, **63**(11):718–25.

296. Stein D, Peri T, Edelstein E, Elizur A, Floman Y: **The efficacy of amitriptyline and acetaminophen in the management of acute low back pain.** *Psychosomatics* 1996, **37**:63–70, [http://dx.doi.org/10.1016/S0033-3182(96)71600-6].

297. Steiner D, Munera C, Hale M, Ripa S, Landau C: **Efficacy and safety of buprenorphine transdermal system (BTDS) for chronic moderate to severe low back pain: a randomized, double-blind study**. *J Pain* 2011, **12**(11):1163–73.

298. Steiner D, Munera C, Hale M, Ripa S, Landau C: **The efficacy and safety of buprenorphine transdermal system (BTDS) in subjects with moderate to severe low back pain: A double-blind study**. *Journal of Pain* 2009, **10**(4):S51.

299. Steiner DJ, Sitar S, Wen W, Sawyerr G, Munera C, Ripa SR, Landau C: **Efficacy and safety of the seven-day buprenorphine transdermal system in opioid-naive patients with moderate to severe chronic low back pain: an enriched, randomized, double-blind, placebo-controlled study**. *J Pain Symptom Manage* 2011, **42**(6):903–17.

300. Strong LL, Von Korff M, Saunders K, Moore JE: **Cost-effectiveness of two self-care interventions to reduce disability associated with back pain**. *Spine.* 2006, **31**(15):1639–45.

301. Stuckey SJ, Jacobs A, Goldfarb J: **EMG biofeedback training, relaxation training, and placebo for the relief of chronic back pain.** *Percept Mot Skills* 1986, **63**(3):1023–1036, [http://dx.doi.org/10.2466/pms.1986.63.3.1023].

302. Subin B, Saleemi S, Morgan G, Zavisca F, Randall C: **Treatment of Chronic Low Back Pain by Local Injection of Botulinum Toxin-A**. *The Internet Journal of Anesthesiology* 2003, **6**(2):1–8.

303. Sutlive TG, Mabry LM, Easterling EJ, Durbin JD, Hanson SL, Wainner RS, Childs JD: **Comparison of short-term response to two spinal manipulation techniques for patients with low back pain in a military beneficiary population**. *Mil Med* 2009, **174**(7):750–6.

304. Tasleem RA, Buth BA, Koul PA, Kadri SM: **Chronic low back pain - Comparative analysis of treatment response to drugs and different physical modalities**. *Jk Practitioner* 2003, **10**(3):201–204.

305. Tavafian SS, Jamshidi A, Mohammad K, Montazeri A: **Low back pain education and short term quality of life: a randomized trial**. *BMC Musculoskelet Disord* 2007, **8**:21.

306. Tavafian SS, Jamshidi AR, Mohammad K: **Treatment of chronic low back pain: a randomized clinical trial comparing multidisciplinary group-based rehabilitation program and oral drug treatment with oral drug treatment alone**. *Clin J Pain* 2011, **27**(9):811–8.

307. Tavafian SS, Jamshidi AR, Montazeri A: **A randomized study of back school in women with chronic low back pain: quality of life at three, six, and twelve months follow-up**. *Spine (Phila Pa 1976)* 2008, **33**(15):1617–21.

308. Tekur P, Chametcha S, Hongasandra RN, Raghuram N: **Effect of yoga on quality of life of clbp patients: A randomized control study**. *Int J Yoga* 2010, **3**:10–7.

309. Tekur P, Singphow C, Nagendra HR, Raghuram N: **Effect of short-term intensive yoga program on pain, functional disability and spinal flexibility in chronic low back pain: a randomized control study**. *J Altern Complement Med* 2008, **14**(6):637–44.

310. Thomas T M; Lundberg: **Importance of modes of acupuncture in the treatment of chronic nociceptive low back pain**. *Acta Anaesthesiol.Scand.* 1994, **38.1**:63–69.

311. Tilbrook HE, Cox H, Hewitt CE, Kang'ombe AR, Chuang LH, Jayakody S, Aplin JD, Semlyen A, Trewhela A, Watt I, Torgerson DJ: **Yoga for chronic low back pain: a randomized trial.** *Ann Intern Med* 2011, **155**(9):569–78.

312. Tobis JS, Hoehler FK: **Musculoskeletal manipulation in the treatment of low back pain**. *Bull N Y Acad Med* 1983, **59**(7):660–8.

313. Torstensen TA, Ljunggren AE, Meen HD, Odland E, Mowinckel P, Geijerstam S: **Efficiency and costs of medical exercise therapy, conventional physiotherapy, and self-exercise in patients with chronic low back pain. A pragmatic, randomized, single-blinded, controlled trial with 1-year follow-up**. *Spine* 1998, **23**(23):2616–2624.

314. Toya Sea: **Report on a computer-randomized double-blind clinical trial to determine the effectiveness of the GaAIA (830 nm) diode laser for attenuation in selected pain groups.** *Laser Therapy* 1994, **6**.

315. Tsui ML, Cheing GL: **The effectiveness of electroacupuncture versus electrical heat acupuncture in the management of chronic low-back pain**. *J Altern Complement Med* 2004, **10**(5):803–9.

316. Tsukayama Hea: **Randomised Controlled Trial Comparing the Effectiveness of Electroacupuncture and TENS for Low Back Pain: A Preliminary Study for a Pragmatic Trial**. *Accupuncture in Medicine* 2002, **20**(4).

317. Underwood M, Mistry D, Lall R, Lamb S: **Predicting response to a cognitive-behavioral approach to treating low back pain: Secondary analysis of the BeST data set**. *Arthritis Care Res (Hoboken)* 2011, **63**(9):1271–9.

318. Vickers AJ: **Statistical reanalysis of four recent randomized trials of acupuncture for pain using analysis of covariance.** *Clin J Pain* 2004, **20**(5):319–323.

319. Videbaek TS, Christensen FB, Soegaard R, Hansen ES, Hoy K, Helmig P, Niedermann B, Eiskjoer SP, Bunger CE: **Circumferential fusion improves outcome in comparison with instrumented posterolateral fusion: long-term results of a randomized clinical trial**. *Spine* 2006, **31**(25):2875–80.

320. Vlaeyen JW, de Jong J, Geilen M, Heuts PH, van Breukelen G: **The treatment of fear of movement/(re)injury in chronic low back pain: further evidence on the effectiveness of exposure in vivo**. *Clin J Pain* 2002, **18**(4):251–61.

321. Walter D, Yelland M: **Re: Yelland M, Glasziou P, Bogduk N, et al. Prolotherapy injections, saline injections and exercises for chronic low-back pain: A randomized trial. Spine 2004; 29:9-16 (1) (multiple letters)**. *Spine* 2004, **29**(19):2195–2196.

322. Warming S, Ebbehoj NE, Wiese N, Larsen LH, Duckert J, Tonnesen H: **Little effect of transfer technique instruction and physical fitness training in reducing low back pain among nurses: a cluster randomised intervention study**. *Ergonomics* 2008, **51**(10):1530–48.

323. Waterworth RF, Hunter IA: **An open study of diflunisal, conservative and manipulative therapy in the management of acute mechanical low back pain**. *N Z Med J* 1985, **98**(779):372–5.

324. Weber Tea: **Traction therapy in patients with herniated lumbar intervertebral discs.** *J Oslo City Hosp* 1984, **34**:61–70.

325. Werners R, Pynsent PB, Bulstrode CJ: **Randomized trial comparing interferential therapy with motorized lumbar traction and massage in the management of low back pain in a primary care setting**. *Spine* 1999, **24**(15):1579–1584.

326. Westrom KK, Maiers MJ, Evans RL, Bronfort G: **Individualized chiropractic and integrative care for low back pain: the design of a randomized clinical trial using a mixed-methods approach**. *Trials* 2010, **11**:24.

327. Wheeler WJ, Gever LN: **Functional status of patients with acute low back pain following treatment with carisoprodol 250-mg tablets assessed by the roland-morris disability questionnaire (RMDQ)**. *Pain Medicine* 2010, **11**(2):305.

328. Whitehurst DG, Lewis M, Yao GL, Bryan S, Raftery JP, Mullis R, Hay EM: **A brief pain management program compared with physical therapy for low back pain: results from an economic analysis alongside a randomized clinical trial**. *Arthritis Rheum* 2007, **57**(3):466–73.

329. Whitman JM, Fritz JM, Childs JD: **The influence of experience and specialty certifications on clinical outcomes for patients with low back pain treated within a standardized physical therapy management program**. *Journal of Orthopaedic and Sports Physical Therapy* 2004, **34**(11):662–672.

330. Wilkey A, Gregory M, Byfield D, McCarthy PW: **A comparison between chiropractic management and pain clinic management for chronic low-back pain in a national health service outpatient clinic**. *J Altern Complement Med* 2008, **14**(5):465–73.

331. Williams KA, Petronis J, Smith D, Goodrich D, Wu J, Ravi N, Doyle EJ, Gregory Juckett R, Munoz Kolar M, Gross R, Steinberg L: **Effect of Iyengar yoga therapy for chronic low back pain**. *Pain.* 2005, **115**(1-2):107–17.

332. Wilson E, Payton O, Donegan-Shoaf L, Dec K: **Muscle energy technique in patients with acute low back pain: a pilot clinical trial**. *J Orthop Sports Phys Ther* 2003, **33**(9):502–12.

333. Wilson-MacDonald J, Fairbank J, Frost H, Yu LM, Barker K, Collins R, Campbell H: **The MRC spine stabilization trial: surgical methods, outcomes, costs, and complications of surgical stabilization**. *Spine (Phila Pa 1976)* 2008, **33**(21):2334–40.

334. Yakhno N, Guekht A, Skoromets A, Spirin N, Strachunskaya E, Ternavsky A, Olsen KJ, Moller PL: **Analgesic efficacy and safety of lornoxicam quick-release formulation compared with diclofenac potassium: randomised, double-blind trial in acute low back pain**. *Clin Drug Investig* 2006, **26**(5):267–77.

335. Yu F, Morgenstern H, Hurwitz E, Berlin TR: **Use of a Markov transition model to analyse longitudinal low-back pain data**. *Stat Methods Med Res* 2003, **12**(4):321–31.

336. Zaina F, Vismara L, Menegoni F, Galli M, Negrini S, Villa V: **Clinical and kinematic evaluation of osteopathy vs specific exercises in obese non-specific chronic low back pain females patients: A randomized controlled trial**. *Spine* 2010.
