# Supplementary figures and images for: A Systematic Review of Outcome Measures Use, Analytical Approaches, Reporting Methods, and Publication Volume by Year in Low Back Pain Trials Published between 1980 and 2012: Respice, adspice, et prospice
Source: PLoS One. 2016 Oct 24;11(10):e0164573. doi: 10.1371/journal.pone.0164573 (PMC5077121; doi:10.1371/journal.pone.0164573)

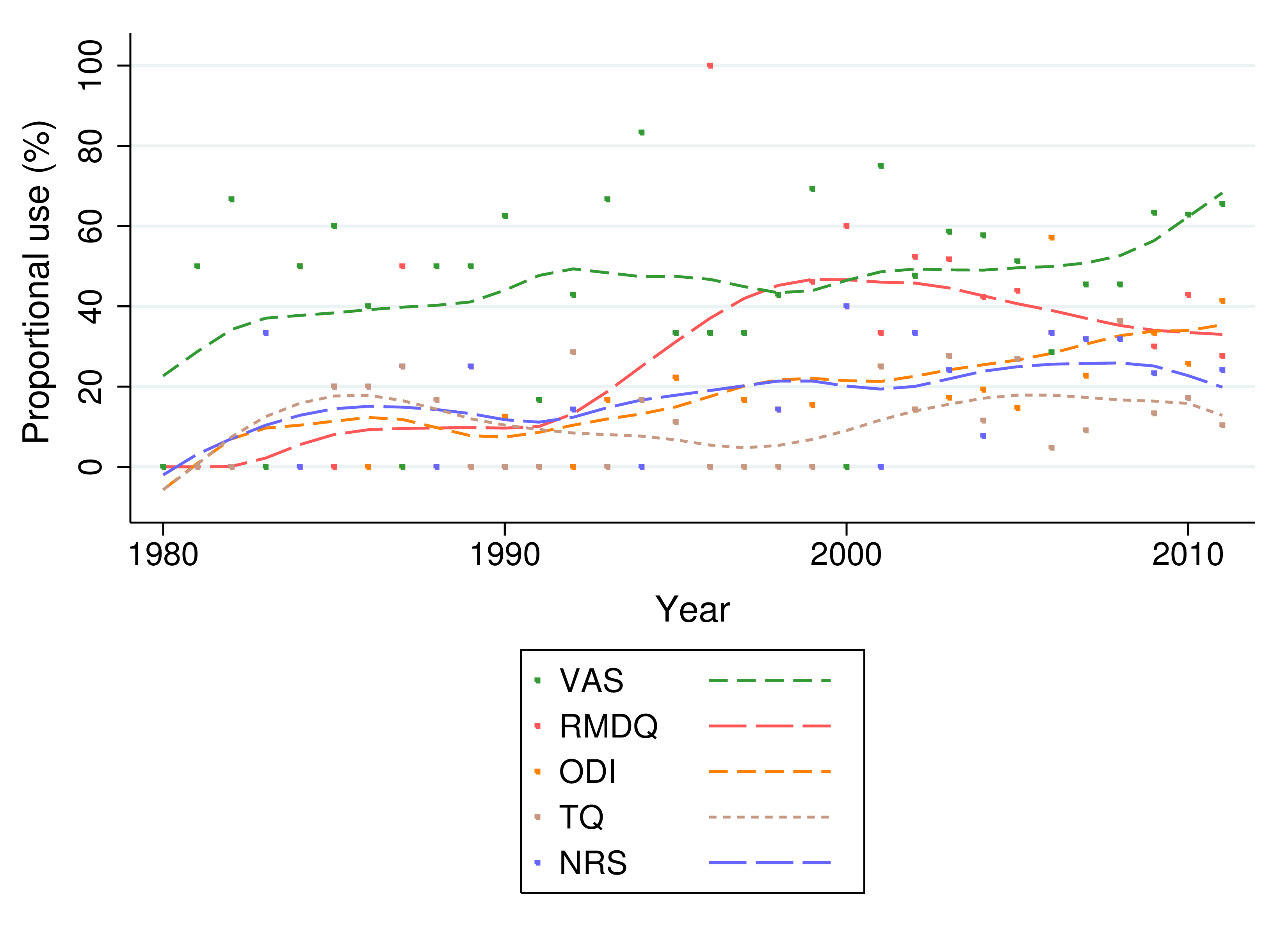

Supplement: S1 Fig — The figure shows the most commonly used measures over time, standardised by the number of annual publications, using a Lowess smoother of half the bandwidth of that shown in Fig 5, in case more sensitive trend-lines are preferred. (TIF) [file pone.0164573.s004.tif]
